# Data-Driven Assisted Decision Making for Surgical Procedure of Hepatocellular Carcinoma Resection and Prognostic Prediction: Development and Validation of Machine Learning Models

**DOI:** 10.3390/cancers15061784

**Published:** 2023-03-15

**Authors:** Liyang Wang, Danjun Song, Wentao Wang, Chengquan Li, Yiming Zhou, Jiaping Zheng, Shengxiang Rao, Xiaoying Wang, Guoliang Shao, Jiabin Cai, Shizhong Yang, Jiahong Dong

**Affiliations:** 1 School of Clinical Medicine, Tsinghua University, Beijing 100084, China; 2 Hepato-Pancreato-Biliary Center, Beijing Tsinghua Changgung Hospital, School of Clinical Medicine, Tsinghua University, Beijing 102218, China; 3 Department of Interventional Therapy, The Cancer Hospital of the University of Chinese Academy of Sciences (Zhejiang Cancer Hospital), Institute of Basic Medicine and Cancer (IBMC), Chinese Academy of Sciences, Hangzhou 310022, China; 4 Department of Liver Surgery, Key Laboratory of Carcinogenesis and Cancer Invasion of Ministry of Education, Liver Cancer Institute, Zhongshan Hospital, Fudan University, Shanghai 200032, China; 5 Department of Radiology, Zhongshan Hospital, Fudan University, Shanghai 200032, China; 6 Department of Hepatobiliary and Pancreatic Surgery, The Cancer Hospital of the University of Chinese Academy of Sciences (Zhejiang Cancer Hospital), Institute of Basic Medicine and Cancer (IBMC), Chinese Academy of Sciences, Hangzhou 310022, China; 7 Department of Radiology, The Cancer Hospital of the University of Chinese Academy of Sciences (Zhejiang Cancer Hospital), Institute of Basic Medicine and Cancer (IBMC), Chinese Academy of Sciences, Hangzhou 310022, China

**Keywords:** surgical procedure, decision-making, prognostic prediction, deep learning, ensemble learning

## Abstract

**Simple Summary:**

Clinically, surgical decisions for HCC resection are difficult and not sufficiently personalized. The aim of this study was to develop a surgical procedure decision-making and effectiveness prediction system for hepatectomy through extensive expert surgical decision-making experience and long-term follow-up, which can assist surgeons. The proposed machine learning models demonstrated their superior performance in surgical decision-making, OS, and RFS prediction tasks. Additionally, a web server that aids physician decision-making was deployed, which is expected to be an effective tool for auxiliary surgeons.

**Abstract:**

Background**:** Currently, surgical decisions for hepatocellular carcinoma (HCC) resection are difficult and not sufficiently personalized. We aimed to develop and validate data driven prediction models to assist surgeons in selecting the optimal surgical procedure for patients. Methods**:** Retrospective data from 361 HCC patients who underwent radical resection in two institutions were included. End-to-end deep learning models were built to automatically segment lesions from the arterial phase (AP) of preoperative dynamic contrast enhanced magnetic resonance imaging (DCE-MRI). Clinical baseline characteristics and radiomic features were rigorously screened. The effectiveness of radiomic features and radiomic-clinical features was also compared. Three ensemble learning models were proposed to perform the surgical procedure decision and the overall survival (OS) and recurrence-free survival (RFS) predictions after taking different solutions, respectively. Results**:** SegFormer performed best in terms of automatic segmentation, achieving a Mean Intersection over Union (mIoU) of 0.8860. The five-fold cross-validation results showed that inputting radiomic-clinical features outperformed using only radiomic features. The proposed models all outperformed the other mainstream ensemble models. On the external test set, the area under the receiver operating characteristic curve (AUC) of the proposed decision model was 0.7731, and the performance of the prognostic prediction models was also relatively excellent. The application web server based on automatic lesion segmentation was deployed and is available online. Conclusions**:** In this study, we developed and externally validated the surgical decision-making procedures and prognostic prediction models for HCC for the first time, and the results demonstrated relatively accurate predictions and strong generalizations, which are expected to help clinicians optimize surgical procedures.

## 1. Introduction

As the dominant form of primary liver cancer, hepatocellular carcinoma (HCC) ranks sixth in terms of incidence and is the fourth most common cause of cancer-related death globally [1,2,3]. Hepatectomy is currently one of the most effective curative treatments [4], while the long-term oncological outcome is still poor due to high recurrence rates and metastasis after surgery [5]. With the development of precision surgery [6], modern hepatectomy has shown relatively satisfactory clinical responses for the treatment of HCC [7].

Hepatectomy consists of an anatomical resection (AR), which is the complete removal of anatomically separate segments, subsegments, or combined segments, and non-anatomical resection (NAR), which focuses on reducing the distance between the cut edges and preserving as many functional remnants of liver parenchyma as possible [8]. However, the different procedures have their own advantages and disadvantages, and the best is still controversial [9,10]. AR may be at a relative disadvantage in terms of intraoperative bleeding, operative time, and postoperative complication rate, but many studies have demonstrated that AR may have advantages over NAR in terms of overall survival (OS) and recurrence-free survival (RFS) [11,12]. Recent reports in this field focus on comparing the two procedures. For example, Minagawa et al. [12] retrospectively analyzed HCC patients treated with AR or NAR at two institutions between 2004 and 2017. Comparison by propensity score matching (PSM) revealed that AR reduced recurrence after initial hepatectomy. Meng et al. [13] grouped HCC patients for microvascular invasion (MVI) and then compared early recurrence rates after AR or NAR.

Clinically, the choice of AR or NAR depends on the patients’ liver function reserve and the radical nature of the operation. However, individualized decision-making during the surgical procedure is still difficult and relies mainly on physician experience. This clinical decision usually requires consideration of the patient’s tumor borders, the presence of significant cysts, the presence of satellite lesions, or the width of the tumor resection margin. In particular, patients with a Child-Pugh of A grade, an Indocyanine Green Retention at 15 min (ICG R15) of less than 10%, and a ratio of essential to standard liver volume (RES) of greater than 40% have no safety restrictions on the extent of resection, i.e., both AR and NAR can be performed, making the decision for the procedure and the prediction of its effectiveness more clinically significant. Therefore, relying on expert experience to develop personalized surgical-assisted decision making and corresponding prognostic prediction methods can enhance the treatment prescribed by inexperienced physicians. With the rise of the artificial intelligence (AI) industry, machine learning techniques have played an outstanding role in the risk assessment [14], assisted diagnosis [15,16], and prognosis prediction of HCC [17,18]. However, there have been no reports employing machine learning to develop surgical-assisted decision making for the surgical procedure of HCC resection.

This work focused on surgical decision-making and effective prognostic prediction models in HCC patients with normal liver functional reserve. To the best of our knowledge, this is the first study reporting on surgical-procedure-assisted decision making using machine learning for HCC patients. We trained the models from a large number of experts’ decision records so that they learned valuable expert experience, which can provide a personalized surgical procedure plan for each patient. Additionally, the probability of OS and RFS after undergoing the recommended procedure can also be calculated by the proposed models, which facilitates the physician’s assessment of its effectiveness. The external validation results show that the proposed method has a relatively strong generalization ability and is expected to be a clinical decision aid for physicians. We also trained deep learning automatic segmentation models for end-to-end applications. The web server application based on the model deployment is already available online and is easy to operate and convenient for surgeons to use.

Our study has the following highlights:

1. A data-driven HCC surgical-assisted decision-making system was developed for the first time, and relatively satisfactory results were obtained from the external test set.
2. Three high-performing ensemble learning models were developed, all of which outperformed the mainstream ensemble models. Based on this, the associated web server was deployed.
3. After rigorous feature screening, efficient feature expression (radiomics-clinical) was explored.

## 2. Materials and Methods

**Figure 1:**
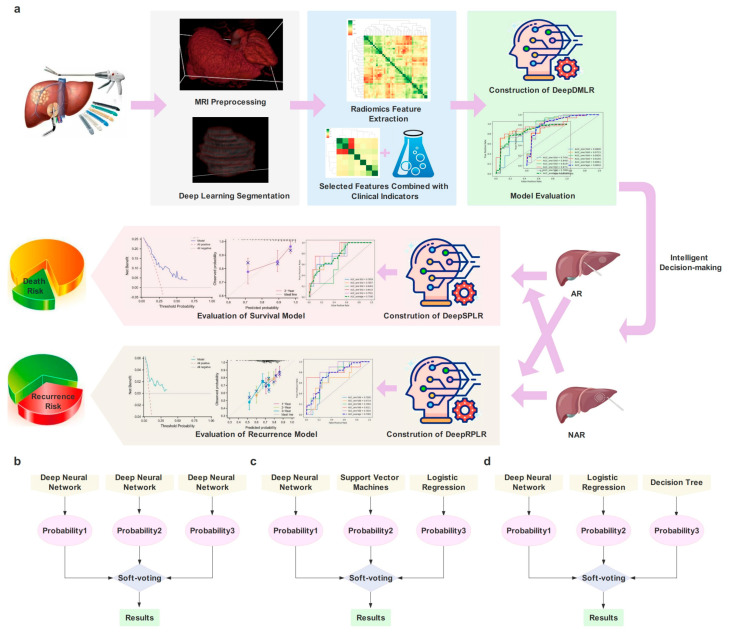
(**a**) is the workflow of this study; (**b**–**d**) represent the architecture of the proposed models, including the Deep Model of Decision-making in Liver Resection (DeepDMLR), the Deep Model of Survival Prediction about Liver Resection (DeepSPLR), and the Deep Model of Recurrence Prediction about Liver Resection (DeepRPLR), respectively.

The workflow of this study is shown in Figure 1a.

### 2.1. Patients Enrollment

**Figure 2:**
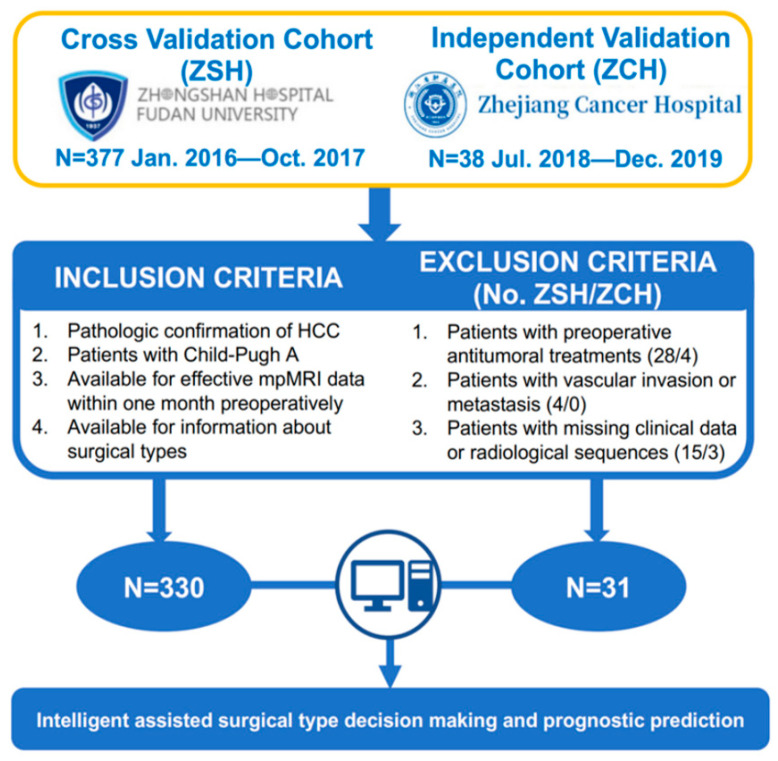
The inclusion and exclusion criteria and statistics of patients.

This retrospective study was conducted in accordance with the Declaration of Helsinki and was approved by the Institutional Review Boards of Zhongshan Hospital, Fudan University (ZSH, Shanghai, China), and Zhejiang Cancer Hospital (ZCH, Hangzhou, China), and written informed consent was waived by the Institutional Review Boards of both centers. Two independent cohorts of preoperative dynamic contrast enhanced magnetic resonance imaging (DCE-MRI) from 415 primary HCC patients who received radical hepatectomy were collected in this study: ZSH cohort (n = 377) and ZCH cohort (n = 38). The inclusion criteria of patients in this study were as follows: (1) patients with primary HCC confirmed by histology after surgical resection; (2) patients with available DCE-MRI and clinical data within one months before surgery; (3) patients that had detailed information about surgical types; (4) patients with Child-Pugh grading of A. The exclusion criteria of patients in this study were as follows: (1) patients who received preoperative anti-tumoral therapies or hepatectomy (ZSH, ZCH cohort with 28, 4 patients, respectively); (2) patients with macrovascular invasion and extrahepatic metastasis on the radiological assessment (ZSH, ZCH cohort with 4, 0 patients, respectively); (3) patients with missing clinical data or ineffective radiological sequences (ZSH, ZCH cohort with 15, 3 patients, respectively). Subsequently, a total of 361 patients, 330 patients from ZSH cohort and 31 from ZCH cohort, were enrolled in this study. The specific incorporation and exclusion process is shown in Figure 2.

### 2.2. Decision-Making of Surgical Procedure by the Experienced Surgeons

The criteria for radical hepatectomy are that no residual tumor is found at the resection margin, no tumor is found in the remaining liver, and tumor markers return to normal within two months postoperatively. The definition of AR is the systematic resection of a hepatic segment confined by tumor-bearing portal tributaries. All patients underwent preoperative liver function reserve assessment to ensure surgical safety before surgery, including liver function tests, coagulation tests, liver stiffness by FibroScan, and/or preoperative 3D reconstruction. For patients with a background of cirrhosis, preoperative ICG clearance tests as well as residual liver volume estimation were conducted. All of the above items are routine preoperative examinations for HCC patients in the included centers. Each patient’s surgical plan, especially the basis for the surgical procedure decision, was assessed and discussed preoperatively by the same experts according to uniform criteria. Clinically, the choice between AR and NAR depends on the balance between the liver function reserve and surgical radicality. AR is usually chosen for patients with unclear tumor borders and no obvious capsule, displayed satellite foci, or an estimated narrow tumor resection margin (<1 cm). Otherwise, NAR with preservation of liver parenchyma is preferred. Both AR and NAR were performed by the same experienced surgical specialists.

### 2.3. Follow-Up

All patients were recommended for follow-up visits for the first month after hepatic resection and every 2 or 3 months thereafter in both centers. Follow-up assessments included laboratory examinations, chest X-ray or computed tomography (CT), abdominal ultrasonography, and/or either CT or MRI, etc. Recurrence was defined as typical imaging appearance or pathological confirmation by the repeated hepatectomy. OS was defined as the time from the date of the liver resection to the date of death or the latest follow up, and RFS was defined as the periods from the date of hepatectomy to the date of recurrence or the latest follow-up.

### 2.4. Clinical Characteristics Selection

Baseline characteristics were collected from electronic medical records from the two centers, including demographic data (sex and age), etiology (hepatitis virus B or C carriers), Barcelona Clinic Liver Cancer (BCLC) grade, etc. Preoperative laboratory examinations were also recorded, including routine blood tests, blood biochemical tests, blood coagulation function tests, tumor markers (alpha-fetoprotein [AFP] and carcinoembryonic antigen [CEA]), etc. Although some postoperative indicators, such as MVI, are valuable for decision making and prognosis prediction, this work aimed to develop models to assist physicians in preoperative decision making, so relevant factors were not included. Additionally, tumor number and size were also collected but not fed into the model because of repeated extraction on radiomics features and BCLC grade. The baseline data collected were selected to ensure the validity of the input features. Cox regression was employed to screen features related to OS and RFS, respectively. Specifically, univariate analysis of all clinical variables was carried out, and their Hazard Ratio (HR) and p values were calculated. Subsequently, a multivariate analysis was performed on the filtered variables. Finally, prognostic (OS or RFS)-related features were all adopted as clinical baselines for decision-making models.

### 2.5. Image Acquisition, Annotation and Preprocessing

The MRI protocols in the ZSH cohort were performed on a 1.5-T (Avanto, Siemens, Erlangen, Germany and Aera, Siemens, Erlangen, Germany) or on 3.0 T scanners (Magnetom Verio, Siemens Medical Solution, Erlangen, Germany and Signa HDx, GE Healthcare, Milwaukee, WI, USA) with gadopentetate dimeglumine (Magnevist; Bayer Schering Pharma AG, Berlin, Germany). In addition, patients in the ZCH cohort underwent preoperative MRI with Gadodiamide injection (GE Healthcare, Dublin, Ireland) on a Siemens AG Magnetom Verio 3.0 T (Munich, Germany). Seven sequences were obtained for each patient [19], but only the arterial phase (AP) (about 20–30 s) was included in the study because arterial phase was found to have a better experimental performance compared to other sequences in our preliminary experiment.

The region of the largest diameter of the tumor in the images of each MR sequence was outlined by using ITK SNAP software (version 3.6.0; www.itk-snap.org (accessed on 1 March 2023)). All tumor regions were annotated by an interventional surgeon (S.D.J.) and an abdominal radiologist (W.W.T.) with 5 and 8 years of experience in reading abdominal MRIs, respectively. Images were then reviewed and corrected by a senior radiologist (S.G.L.) and a senior hepatic surgeon (C.J.B.), who both had more than 10 years of experience in reading abdominal MRIs. To evaluate the reproducibility of tumoral segmentations, the inter-reader and intrareader dice similarity coefficient (DSC) between the two radiologists were additionally calculated between segmentations from fifty randomly selected enrolled patients. It is worth noting that the segmentation of the external test data was performed by the proposed automatic segmentation model. Resampling of images by interpolation was necessary due to the multi-centered data involved. Additionally, cropping slices that did not involve tumors and data augmentation operations (including random rotation, flipping, etc.) were utilized.

### 2.6. Automatic Segmentation Models Construction

To automate surgical-procedure-assisted decision making, deep learning-based lesion segmentation models were proposed. In this study, a lightweight transformer architecture called Segformer [20] was adopted for automatic segmentation of liver tumors, which not only reduced the difficulty of training, but also verified better performance [21]. The architecture includes a novel hierarchical Transformer encoder and a lightweight all-multilayer perceptron (MLP) decoder. Compared with the previous Transformers, Segformer greatly reduces the number of parameters and calculations [20], which is conducive to its embedding into hospital terminal systems. In this study, a pre-trained model (mix_vision_transformer_b5) was employed for transfer learning in Segformer. MixVisionTransformer_B5 was selected as Backbone, and embedding dim was set to 768. To reflect the superiority of the proposed model, both U-Net [22] and U-Net++ [23] were performed under equal conditions as the comparison. In this paper, slices (size converted to 512 × 512) were fed into the models in bulk, and the output results were reconstructed into 3 dimensions. All proposed automatic segmentation models were trained with 50,000 iterations to ensure full convergence and no overfitting. The modeling process was completed in Python 3.7 with the help of the Paddle-Paddle 2.0 deep learning framework.

### 2.7. Radiomics Features Extraction and Selection

For segmented lesions, radiomic features were extracted as one of the model inputs. The Pyradiomics 3.0.1 library in Python 3.7 was used to extract a total of 980 radiomic features of HCC, including Shape, Firstorder, GLRLM, GLSZM, GLDM and NGTDM. A total of 14 transformations were included in each type of feature, namely Original, Wavelet-LLH, Wavelet-LHL, Wavelet-LHH, Wavelet-HLL, Wavelet-HLH, Wavelet-HHL, Wavelet-HHH, Wavelet-LLL, Square, Squareroot, Logarithm, Exponential, and Gradient. Considering that high-dimensional features may lead to model overfitting, this study screened the extracted features to obtain effective results. Firstly, Z-score normalization was executed, which converted data of different magnitudes into the same magnitude uniformly and measured them with the calculated Z-score values to ensure comparability among data. Then, the Least Absolute Shrinkage Selector Operator (LASSO) regression algorithm was employed to screen all features. The glmnet 4.1 package in R 4.1.3 was selected to train the model and perform 5-fold cross validation. Finally, effective features were determined by optimizing parameter tuning.

### 2.8. Decision Making and Prognosis Models Construction

In order to explore superior models, three novel ensemble learning architectures were constructed for decision-making, OS and RFS prediction tasks, namely, Deep Model of Decision-making of Liver Resection (DeepDMLR), Deep Model of Survival Prediction after Liver Resection (DeepSPLR) and Deep Model of Recurrence Prediction after Liver Resection (DeepRPLR), respectively. Three mainstream ensemble learning models were also adopted for comparison. The specific model architecture and core parameters of each model are shown in Table S1. In each model, radiomic features and radiomic features combined with clinical baseline data were inputted separately to explore better feature representation. Due to the imbalance in the obtained data, all partitioned training sets were subjected to the Synthetic Minority Over-Sampling Technique (SMOTE) to prevent overfitting. This work involved using the scikit-learn 1.1.3 library in Python 3.7.

#### 2.8.1. Deep Model of Decision-Making of Liver Resection (DeepDMLR)

For the characteristics of the assisted decision-making task, we constructed a classifier integrated with three different deep neural networks (DNNs) and selected Soft-voting to obtain the final probability (Figure 1b). Each neural network contained 3,4,3 hidden layers, respectively, and all of them adopted ReLU as the activation function and Adam as the optimizer. Additionally, the initial learning rate was set to 0.0001.

#### 2.8.2. Deep Model of Survival Prediction after Liver Resection (DeepSPLR)

DeepSPLR focuses on the probability prediction of OS within 3 years after undergoing different surgical procedures. After selecting each follow-up record, 89 (76 in the training cohort and 13 in the external test cohort) and 246 (228 in the training cohort and 18 in the external test cohort) patients were eligible in the AR and NAR groups, respectively. DeepSPLR was obtained by integrating three base classifiers, including DNN, Support Vector Machine (SVM) and Logistic Regression (LR) (Figure 1c). DNN included three hidden layers, the kernel function of SVM was RBF, and the penalty factor C of SVM and LR was set to 0.1.

#### 2.8.3. Deep Model of Recurrence Prediction after Liver Resection (DeepRPLR)

The proposed DeepRPLR was employed to predict the probability of RFS after different surgical decisions. In the AR group, 77 (training cohort: 66; test cohort: 11), 82 (training cohort: 71; test cohort: 11), and 85 (training cohort: 73; test cohort: 12) patients were eligible for predictions within 1, 2, and 3 years postoperatively, respectively. In the NAR group, 207 (training cohort: 192; test cohort: 15), 231 (training cohort: 214; test cohort: 17), and 241 (training cohort: 224; test cohort: 17) patients were eligible for predictions within 1, 2, and 3 years postoperatively, respectively. DeepRPLR contained DNN, LR, and Decision Tree (DT) models, which were integrated by Soft-voting (Figure 1d). Max_depth was set to 80 in DT.

#### 2.8.4. Model Comparison

In order to compare the superiority of the proposed models, we selected three mainstream ensemble learning models, namely Gradient Boosting Decision Tree (GBDT), Random Forest (RF), and extreme Gradient Boosting (XGBoost). Previous similar studies using the above algorithms are listed [24,25,26,27]. The parameters were optimized during the training of each model.

### 2.9. Experimental Setup

In this study, ZSH and ZCH cohorts were used as training and testing cohorts, respectively. Five-fold cross-validation was performed within the training cohort to explore the most superior models, and only the models that worked best were externally validated through the test cohort. This work included the training and validation of the assisted decision-making, OS prediction within 3-year and RFS prediction within 1, 2, and 3-year models, the latter two of which were performed in subgroups (AR and NAR groups). The grid search method was used for hyperparameter optimization during the models’ training. The computing device was equipped with CPU AMD Ryzen 7 5800H (16 GB memory) and GPU NVIDIA^®^ Tesla V100 (32 GB memory), supporting acceleration of Computing Unified Device Architecture (CUDA). All the work was completed in the Windows 10 operating system.

### 2.10. Statistics

Baseline data screening was performed by univariate and multivariate Cox regression, and the results were expressed as HR with its 95% confidence interval (CI). Meanwhile, the mean and standard deviation (SD) and median and interquartile range (IQR) denoted normal and non-normal baseline data distributions, respectively. The statistical methods involved were Student and Wilcoxon tests. For categorical variables, the proportion of the total (%) was counted, and statistical methods included Fisher and chi-square tests. The performance evaluation of automatic segmentation used Mean Intersection over Union (mIoU), which is a standard measure of semantic segmentation [28]. The area under the receiver operating characteristic curve (AUC) was selected as the evaluation indicator of the models and was expressed as the mean and its 95% CI during the 5-fold cross-validation. The calibration curve and the decision curve analysis (DCA) were also adopted to reflect the model’s performance. The criterion for the significant difference in results was *p* < 0.05.

## 3. Results

### 3.1. Patient’s Demographics and Baseline Indicators

For prognosis prediction, the screening results of the clinical baseline indicators are shown in Tables S2 and S3. After univariate regression analysis, 15 and 14 potentially associated clinical features were included in the OS group and RFS group, respectively, based on the results of the regression analysis and prior clinical experience. However, based on the results of the multivariate analysis, 9 and 11 features were finally included in the OS and RFS groups, respectively. The clinical baseline variables in the decision-making models included all the features obtained from the above screenings, which were 16 features in total. The baseline statistics between the AR and NAR groups are shown in Table 1. In the ZSH cohort, five indicators, including white blood cells (WBC), platelet count (PLT), aspartate aminotransferase (AST), γ-glutamyl transpeptadase (GGT), and cirrhosis, were statistically significantly different (*p* < 0.05) between the AR and NAR groups. In the ZCH cohort, only GGT showed a significant difference (*p* < 0.05).

### 3.2. Segmentation Results

Compared with other automatic segmentation algorithms, Segformer performed the best on the validation set, with mIoU, Accuracy (Acc), Kappa value, and Dice coefficient reaching 0.8860, 0.9946, 0.8720 and 0.9360, respectively. The mIoU, Acc, Kappa, and Dice were 0.8272, 0.9915, 0.7931, and 0.8965 for U-Net and 0.8151, 0.9910, 0.7754, and 0.8877 for U-Net++, which indicated that they were not as performant as Segformer. The effect of the radiologist’s annotation and automatic segmentation of the three models is shown in Figure S1 (the green area is the delineated lesion). It can be found that the lesions segmented by Segformer are the closest to the real situation. Based on this, we chose the Segformer model to automatically segment the lesions of patients in the external test cohort, which greatly improved the efficiency and facilitated end-to-end clinical applications.

### 3.3. Radiomics Features Included

After calculation, the extracted radiomic features specifically included 14 Shapes, 252 Firstorders, 224 GLRLMs, 224 GLSZMs, 196 GLDMs and 70 NGTDMs. All features were dimensionalized, employing normalization and the LASSO algorithm, and the 5-fold cross-validation results are shown in Figure S2, which demonstrate the different AUC values obtained with the variation of the parameter λ. During this process, all models converged completely, and Figure S3 plots the visualization process of each model training in detail. Ultimately, 6, 7, 13, 33, 41, 23, 9, 13, and 6 features were screened in the models for the assisted decision making of liver resection, OS prediction within 3 years in the AR group, OS prediction within 3 years in the NAR group, and RFS prediction within 1, 2, and 3 years in the AR and NAR groups, respectively (see Table S4 for specific feature names). Based on this, heat maps (Figure S4) were drawn to reflect the correlation between the features.

### 3.4. Results of Decision-Making and Prognosis Prediction Models

#### 3.4.1. DeepDMLR and Comparison Models

**Figure 3:**
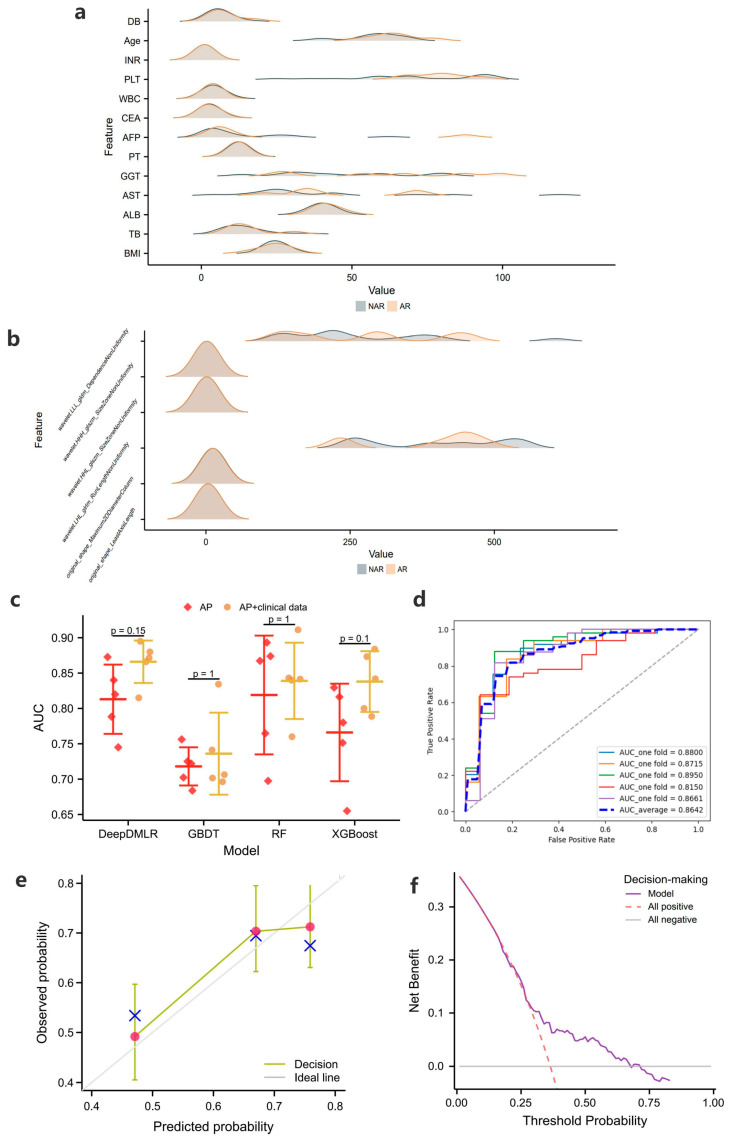
(**a**) and (**b**) show the distribution of clinical baseline and radiomic features incorporated into the decision-making models for the two procedures, respectively (note: clinical features are shown only for continuous data, and extreme values are excluded to show the effect); (**c**) shows the 5-fold cross-validation results of the decision-making models in the training cohort; (**d**–**f**) show the receiver operating characteristic curve (ROC), calibration curve and the decision curve analysis (DCA) of the proposed models when clinical + radiomic features are entered, respectively.

Patients who underwent different surgical procedures may have differences in the distribution of tumor radiomics and clinical features, and Figure 3a,b indicate the distribution of clinical baseline and radiomic features between the AR and NAR groups, respectively. A certain degree of association between patient characteristics and the procedure taken can be found, which supports the interpretability of the decision-making model. Among the relevant clinical features, age, ALT, AFP, GGT, and AST differed significantly between the AR and NAR groups. Among the relevant radiomic features, wavelet-LLL and wavelet-LHL differed significantly between the AR and NAR groups.

The results of the 5-fold cross validation are shown in Figure 3c, from which it can be concluded that the average performance of DeepDMLR (the highest AUC = 0.8642) was better than that of GBDT (the highest AUC = 0.7347), RF (the highest AUC = 0.8375) and XGBoost (the highest AUC = 0.8373). The model effects after inputting AP with the clinical baseline were generally better than those after inputting AP only, which provides a basis for exploring the superior feature representation. Figure 3d and Figure S5 show the receiver operating characteristic curves (ROCs) of DeepDMLR inputting AP + clinical features and other models, respectively. In addition, the calibration curve (Figure 3e) and DCA (Figure 3f) of the best-performing model were also displayed. A strong match between true and predicted probabilities was observed from the calibration curve, and a good net clinical benefit was found from the DCA.

#### 3.4.2. DeepSPLR and Comparison Models

**Figure 4:**
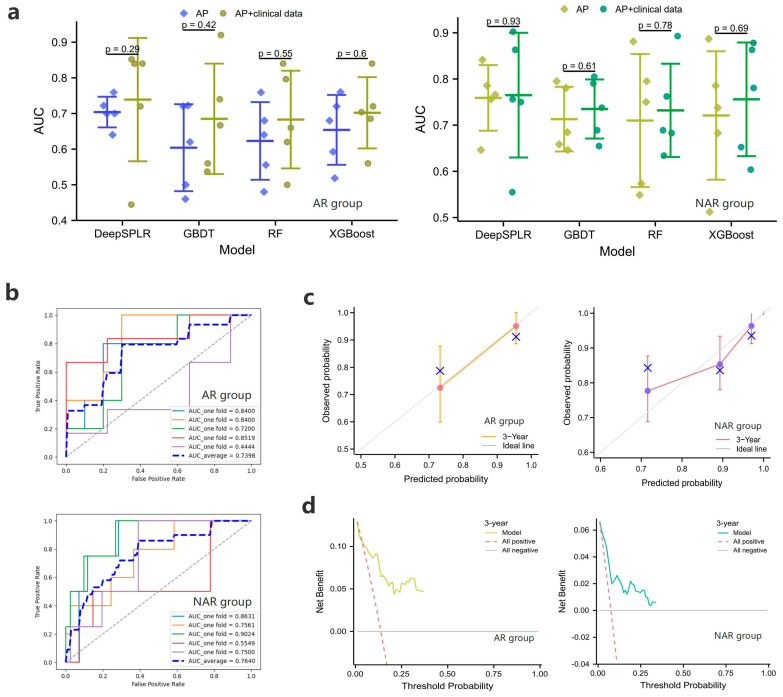
(**a**) represent the 5-fold cross-validation results of the overall survival (OS) prediction models in the training cohort within 3 years after anatomical resection (AR) or non-anatomical resection (NAR); (**b**–**d**) show the ROC curves, calibration curves and the DCAs of the proposed models when clinical + radiomic features are entered, respectively.

For the OS prediction within 3 years after surgery, Figure 4a shows the 5-fold cross-validation results of the four models, and it can be found that DeepSPLR shows the most superior performance in both the AR and NAR groups. The average AUCs of DeepSPLR inputting AP and AP + clinical features in the NAR group (0.7590 and 0.7640) were higher than those in the AR group (0.7048 and 0.7398), respectively. Based on this, we found that the model worked better when the input was AP with clinical data, compared to when the input was AP only. Figure 4b, Figures S6 and S7 represent the ROC curves of DeepSPLR inputting AP + clinical data and other models in the AR and NAR groups. To further demonstrate the performance of the proposed model, Figure 4c,d represents the calibration curves and DCAs of DeepSPLR after inputting the combined features.

#### 3.4.3. DeepRPLR and Comparison Models

**Figure 5:**
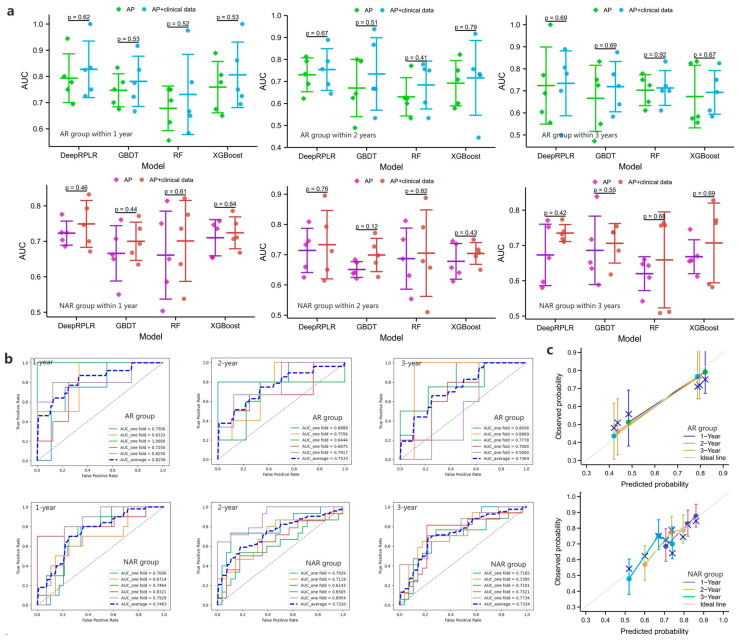

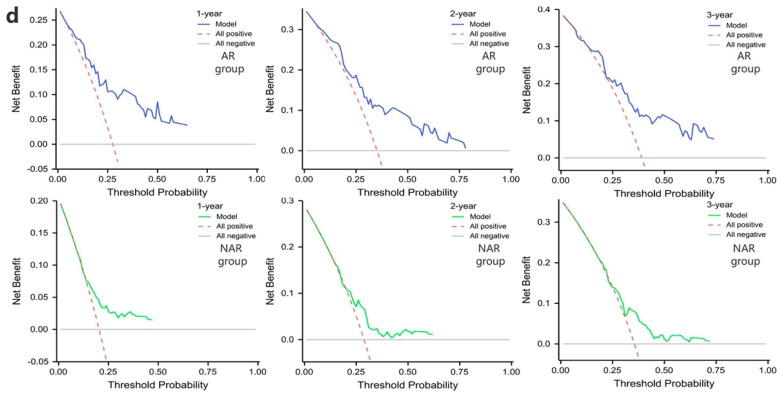
(**a**) represent the 5-fold cross-validation results of the recurrence-free survival (RFS) prediction models in the training cohort within 1, 2, and 3 years after AR or NAR, respectively; (**b**–**d**) show the ROC curves, calibration curves and the DCAs of the proposed models when clinical + radiomic features are entered, respectively.

The results of the RFS prediction are shown in Figure 5a, and it can be seen that the proposed DeepRPLR outperformed the other comparable models in both the AR and NAR groups. Among them, the best-performing DeepRPLR had AUCs of 0.8258, 0.7533, 0.7364 within 1, 2, 3 years after AR and 0.7463, 0.7320, 0.7334 after NAR, respectively. Similarly, the performance of input-combined features was superior to that of inputting radiomic features alone. The ROC curves for all the models are shown in Figure 5b and Figures S8–S13, where the best performing models (DeepRPLR inputting AP + clinical data) were selected and their calibration curves and DCAs were plotted (Figure 5c,d) to reflect the prediction accuracy of the models.

### 3.5. External Validation Results

**Figure 6:**
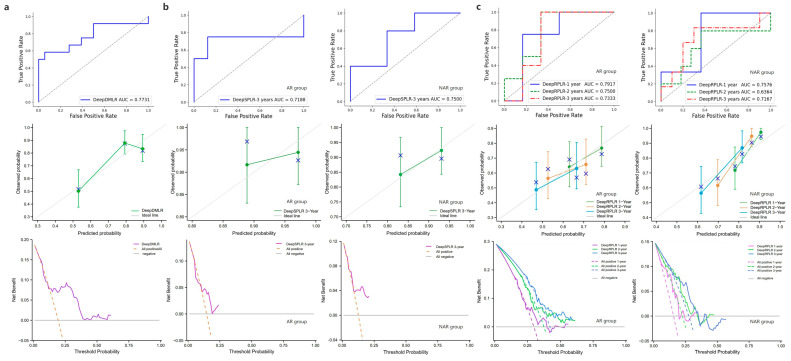
(**a**) denotes the performance of DeepDMLR in the external validation cohort, including its ROC curve, calibration curve and DCA; (**b**) indicates the performance of DeepSPLR for the AR and NAR groups in the external validation cohort, including their ROC curves, calibration curves and DCAs; (**c**) indicates the performance of DeepRPLR for the AR and NAR groups in the external validation cohort, including their ROC curves, calibration curves and DCAs.

In this study, the best-performing model of each task was selected from 5-fold cross-validation in the training cohort and used for testing in the external cohort. The test results of DeepDMLR are shown in Figure 6a. The model achieved an AUC of 0.7731, which indicated its strong generalizability. Additionally, a relatively good decision accuracy and net clinical benefit were reflected from the calibration curve and the DCA. The OS prediction results within 3 years after surgery of DeepSPLR are shown in Figure 6b. It had better prediction performance in the NAR group (AUC = 0.7500) than in the AR group (AUC = 0.7188). Based on this, calibration curves and corresponding DCAs are presented for each group. Similarly, the same evaluation methods were employed to represent the RFS prediction results. In both the AR and NAR test groups (Figure 6c), DeepRPLR had the highest AUC (0.7917 and 0.7576, respectively) within 1 year postoperatively, compared to 2 (0.7500 and 0.6364, respectively) and 3 years (0.7333 and 0.7167, respectively), indicating that it can predict recurrence more accurately in the short term. Moreover, the corresponding calibration curves and DCAs indicate the accuracy and clinical reliability of the models in each group.

## 4. Discussion

This study proposed a novel machine learning architecture to develop and validate the assisted decision-making model for HCC surgical procedures based on the surgical decision experience of a large number of experts. Data-driven development made it intelligent and able to recommend precise and personalized surgical solutions for physicians (the AUC reached 0.7731 in the external test cohort), which was a great first attempt. In addition to comparing the characteristics of AR and NAR in previous reports, this work mainly focused on developing decision-assisted and corresponding prognostic prediction models to address clinical needs. In addition to the recommended surgical procedure, the prediction of OS and RFS after adopting different strategies provided a powerful guide for physicians to make decisions. Moreover, the feature representation and model architecture with superior performance were also explored, which improved the accuracy of prediction.

The SegFormer employed in this work achieved the highest mIoU (0.8860) in the validation data, which demonstrated the accuracy of its segmentation. Therefore, the model was used for the automatic segmentation of patient lesions in the external test cohort. Compared with the traditional medical image segmentation models (U-Net and U-Net++), SegFormer is designed with a lightweight Transformer architecture, which improves segmentation accuracy while reducing model parameters, thus facilitating later deployment. Based on this, SegFormer is expected to be a novel tool for automatic lesion segmentation rather than for manual segmentation, which is important for achieving end-to-end decision making.

The feature representation of the patient is the key to effective model performance, and the combination of effective features can achieve more accurate clinical decision making and prognosis prediction [29,30,31]. The clinical baseline and extracted radiomic features were rigorously screened to ensure the validity of the features in this study. The clinical data obtained first performed univariate regression to screen for potential correlations. However, there might have been problems with collinearity between these variables, so multivariate regression was performed. Eventually, some personal information, such as age, body mass index (BMI), blood test indicators (AST, GGT, etc.), cirrhosis, BCLC grade, and tumor markers (such as AFP and CEA) were included in the study. After the radiomic feature extraction, we first performed a Z-score transformation to standardize the data. The LASSO algorithm was then performed separately for each task to obtain the eigencoefficients, thereby filtering the variables. It is worth noting that, based on previous experience, only AP data were fed into the models in this study, and in the future, we will attempt to combine periods (e.g., AP, DP, in combination with PVP) for input. After expert review, it was confirmed that the included tumor features and clinical baseline features had typical analytical values for surgical plan decision making and prognosis prediction. During the modeling process, two feature representations, including only input tumor features and input tumor + clinical baseline features, were compared to reveal the performance of the models. The results show that the tumor + clinical baseline features representation was superior, which provided experimental support for subsequent development.

For the purpose of exploring the high-performance models, three ensemble learning architectures were designed for assisted decision making, OS prediction and RFS prediction. The types of base classifiers in each architecture were determined on the basis of multiple previous attempts. For each task, we tried different base classifiers, including SVM, RF, LR, K-nearest neighbor, DT, DNN, etc., in order to find the best base models suitable for different tasks. In addition, combinations of different models were tried in pre-experiments to determine the final architecture. The ensemble method was the soft-voting mechanism [32,33,34], which calculated the weighted average of the predicted probabilities in each category by setting the weights, and the category with the largest value was selected. This mechanism incorporates multiple models to reduce error and obtains the final probability by calculating the weighted average probability of each base model, which has been excellent in previous studies [35,36]. In this study, mainstream ensemble learning models, including GBDT, RF and XGBoost, were selected for comparison to verify the superiority of the proposed models. The results of the 5-fold cross validation show that our designed models achieved the highest average AUCs in all tasks, which was encouraging. The results of the external testing show that the proposed models had relatively good generalizations. They can accurately make clinical decisions and prognosis predictions, and have relatively high levels of net clinical benefits.

**Figure 7:**
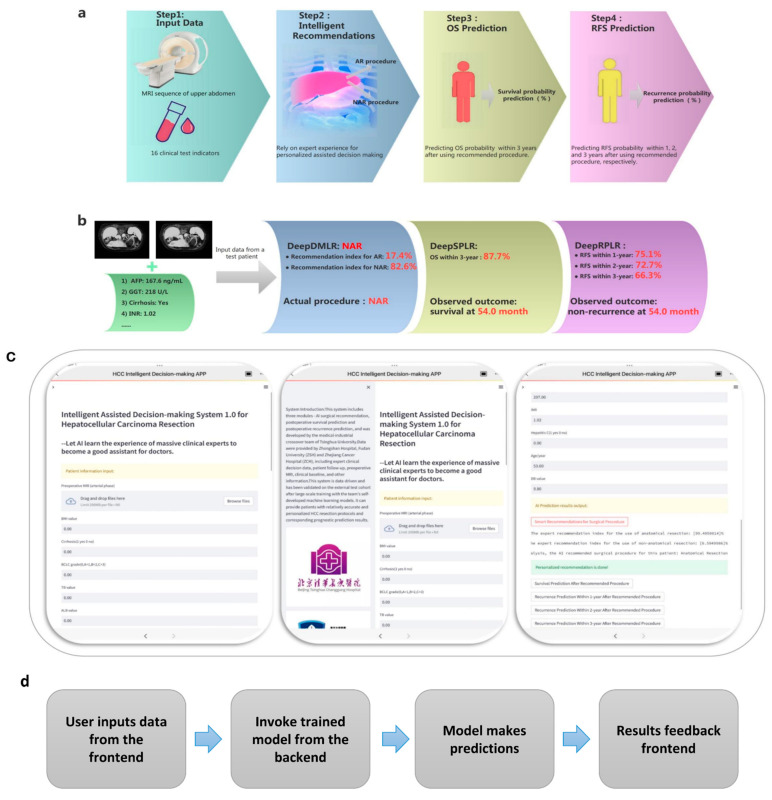
(**a**) is the workflow of the designed end-to-end surgical procedure assisted decision-making system; (**b**) is the test result of a randomly selected patient from the external validation cohort; (**c**) demonstrates the interface of the decision-making system logged in from the iOS client. The images on the left, middle, and right show the patient information input section, the system introduction section, and the result output section, respectively; (**d**) indicates the operational structure of the system.

The challenges encountered during implementation included the difficulty of training the models and optimizing the parameters. In addition, some skills were needed to get the models to converge quickly. Additionally, model deployment required a stable server, which is a challenge for cloud computing platforms. Based on the offline training and external validation described above, the surgical-procedure-assisted decision-making system for HCC was designed to automatically and end-to-end perform online procedure recommendations and prognosis (OS and RFS) predictions after adoption of the recommended option for each patient. The workflow of the system consists of four steps (e.g., Figure 7a). In Step 1, the physician simply enters the patient’s upper abdominal MRI sequence and the specified 16 clinical feature values. In Step 2, the patient’s procedure (AR or NAR) is recommended, and the recommendation factor is calculated. In Step 3, the probability of OS within 3 years after the adoption of this procedure is output. In the final step, the probability of RFS within 1, 2, and 3 years after the patient chooses this procedure is output. The recommended procedure can assist the doctor’s decision making, and the probability of prognosis prediction can reflect the effectiveness of this protocol. We randomly selected a patient from the ZCH cohort and fed it into an assisted decision-making system to assess its reliability, as shown in Figure 7b. Encouragingly, the recommended procedure was consistent with what was actually used, and the predicted prognostic outcomes was the same as that of follow-up records. Based on this, an application web server was deployed at http://43.143.246.77:8502, accessed on 1 March 2023, which can be accessed through the user’s computer or smartphone (the interface of the system is shown in Figure 7c). It enables end-to-end assisted surgical decision making and prognosis prediction and is user friendly, allowing surgeons to do so with just one Internet-connected device (Figure 7d shows the main structure of the proposed system). In the future, more clinical validation will be conducted to evaluate the accuracy and utility of the system.

Stability is a key indicator of a control system and can also be considered as robustness in this study. The robustness of a system is its ability to maintain its original characteristics after external or self-induced changes. In the proposed system, we have tested it for this purpose. Its predictive performance was evaluated using an external test set on the one hand, and the parameters of the system itself were changed on the other, both of which revealed that our system still performed relatively well, which demonstrated its excellent robustness.

Undeniably, the present study also has its limitations. For example, the inadequacy of the dataset, especially the validation data, may affect the vocalization of the model’s reliability and conclusions. Second, the decision models have gained a lot of expert experience, but the expert’s decision may not be the optimal solution in some cases, due to various factors. On the other hand, although the criteria for performing AR and NAR have been strictly defined, there may be differences in operational methods between physicians, which may interfere with the accuracy of the prognostic predictions. Due to data limitations, this work only included patients with normal liver functional reserve. Although this group of patients represents the vast majority of patients who can undergo hepatectomy, the applicability of the models is still somewhat compromised. Imperfect follow-up records of certain patients affect the prognosis prediction over a certain number of years after surgery, and more detailed and long-term follow-ups need to be carried out in the future. In addition, the lack of interpretability due to the black box nature of machine learning still needs to be addressed. Finally, it is worth noting that at present, the system can only assist doctors in their decision making and not replace it, as the judgements made by the intelligent system may differ from the real situation.

## 5. Conclusions

This study developed and validated data-driven surgical-procedure-assisted decision-making models for HCC. The SegFormer model was adopted to automatically segment the patient’s MRI-displayed lesions. The best feature representation that combined clinical indicators and radiomic features was explored. Three ensemble learning models demonstrated their superior performance in surgical decision making, OS, and RFS prediction tasks. Ultimately, the end-to-end system to aid physician decision making was deployed. In addition, this study has a good potential extension value and is expected to be extended to other surgical fields in the future. However, it is undeniable that the operational procedure of our method is relatively cumbersome. Simpler but at the same time more interpretable prediction methods need to be explored in the future.

## Figures and Tables

**Table 1:** Clinical baseline statistics of patients included in the decision-making models.

Characteristic	Training Cohort			External Validation Cohort		
	AR (n = 82)	NAR (n = 248)	*p*	AR (n = 13)	NAR (n = 18)	*p*
Cirrhosis (%)			0.021 *			0.275
No	53 (64.6%)	122 (49.2%)		3 (23.1%)	8 (44.4%)	
Yes	29 (35.4%)	126 (50.8%)		10 (76.9%)	10 (55.5%)	
BCLC grade (%)			0.115			0.208
0	11 (13.4%)	55 (22.2%)		0 (0%)	0 (0%)	
A	66 (80.5%)	170 (68.5%)		9 (69.2%)	16 (88.9%)	
B	5 (6.1%)	23 (9.3%)		4 (30.8%)	2 (11.1%)	
Hepatitis C (%)			0.460			0.419
No	81 (98.8%)	240 (96.8%)		12 (92.3%)	18 (100.0%)	
Yes	1 (1.2%)	8 (3.2%)		1 (7.7%)	0 (0%)	
BMI, median (IQR)	23.88 (21.99, 25.91)	23.88 (21.96, 26.12)	0.770	21.97 (20.4, 24.24)	21.5 (19.64, 26.46)	0.650
TB, median (IQR) or mean ± SD	11.85 (9.62, 15.78)	11.6 (8.57, 15.3)	0.516	12.6 ± 3.55	14.12 ± 5.96	0.419
ALB, median (IQR)	41 (38.25, 44)	42 (39, 44)	0.441	43 (41.2, 45.8)	43.2 (31.02, 46.77)	0.575
AST, median (IQR)	29 (23.25, 43.75)	26.5 (21, 34)	0.010 *	35 (32, 42)	28 (23, 36.5)	0.123
GGT, median (IQR)	52.5 (36, 96.5)	44 (28, 78.25)	0.019 *	68 (35, 117)	33.5 (21, 44.5)	0.041 *
PT, median (IQR)	11.5 (11.03, 12)	11.6 (11.07, 12.2)	0.487	12.4 (11.6, 12.8)	12.2 (11.6, 13)	0.733
AFP, median (IQR)	16.4 (5.4, 536.43)	32.7 (4.4, 395.55)	0.793	10.2 (2.8, 156)	42.4 (2.77, 315.1)	0.603
CEA, median (IQR)	2.2 (1.42, 3.1)	2.3 (1.6, 3.3)	0.482	1.36 (1.11, 2.42)	1.73 (1.01, 2.51)	0.423
WBC, median (IQR)	5.59 (4.57, 6.83)	5.11 (4.04, 6.24)	0.018 *	6.4 (4.5, 8.5)	6.25 (4.5, 7.57)	0.645
PLT, median (IQR) or mean ± SD	164.5 (130.5, 220)	148.5 (113, 184.25)	0.013 *	170.92 ± 75.84	146.11 ± 52.38	0.289
INR, median (IQR)	1 (0.96, 1.07)	1.02 (0.97, 1.08)	0.273	1.03 (0.97, 1.07)	1.04 (0.98, 1.09)	0.269
Age, median (IQR)	56.5 (47, 63.75)	59 (51, 65)	0.104	63 (57, 68)	63.5 (56.5, 68.5)	0.588
DB, median (IQR) or mean ± SD	4.9 (3.92, 6.27)	4.7 (3.7, 6.5)	0.391	5.1 ± 1.71	5.61 ± 2.34	0.514

Note: BCLC: Barcelona Clinic Liver Cancer; BMI: Body Mass Index; TB: Total Bilirubin; ALB: Albumin; AST: Aspartate Aminotransferase; GGT: γ-glutamyl Transferase; PT: Prothrombin Time; AFP: Alpha Fetoprotein; CEA: Carcinoembryonic Antigen; WBC: White Blood Cell; PLT: Platelet Count; INR: International Normalized Ratio; DB: Direct Bilirubin. * *p* < 0.05.

## Data Availability

For the privacy of the patients, the data related to the patients cannot be available for public access, but they can be obtained from the corresponding author upon reasonable request and upon approval from the institutional review board of all enrolled centers.

## Supplementary Materials

The following supporting information can be downloaded at https://www.mdpi.com/article/10.3390/cancers15061784/s1, Figure S1: The visualization effect of automatic segmentation, which shows the comparison of Segformer, U-Net, U-Net++ and manual annotation; Figure S2: The 5-fold cross-validation results of all models using the Least Absolute Shrinkage Selector Operator (LASSO) regression algorithm for feature screening; Figure S3: The convergence process of all Least Absolute Shrinkage Selector Operator (LASSO) regression models, where (a), (b), and (c) are the model convergence results of decision-making, overall survival (OS) prediction, and recurrence-free survival (RFS) prediction, respectively; Figure S4: The screening results of radiomics features in all models, where (a), (b) and (c) are the screening results of model features for decision-making, overall survival (OS) prediction and recurrence-free survival (RFS) prediction, respectively; Figure S5: The 5-fold cross-validation results of all decision-making models in the training cohort, where (a), (b), (c) and (d) represent the receiver operating characteristic (ROC) curves of the proposed model, Gradient Boosting Decision Tree (GBDT), Random Forest (RF) and Extreme Gradient Boosting (XGBoost), respectively (the curves on the left and right are the results of the models with radiomics and radiomics + clinical features as input features, respectively); Figure S6: The 5-fold cross-validation results of all OS prediction models within 3 years after anatomical liver resection (AR) in the training cohort, where (a), (b), (c) and (d) represent the ROC curves of the proposed model, GBDT, RF and XGBoost, respectively (the curves on the left and right are the results of the models with radiomics and radiomics + clinical features as input features, respectively); Figure S7: The 5-fold cross-validation results of all OS prediction models within 3 years after non-anatomical liver resection (NAR) in the training cohort, where (a), (b), (c) and (d) represent the ROC curves of the proposed model, GBDT, RF and XGBoost, respectively (the curves on the left and right are the results of the models with radiomics and radiomics + clinical features as input features, respectively); Figure S8: The 5-fold cross-validation results of all RFS prediction models within 1 year after AR in the training cohort, where (a), (b), (c) and (d) represent the ROC curves of the proposed model, GBDT, RF and XGBoost, respectively (the curves on the left and right are the results of the models with radiomics and radiomics + clinical features as input features, respectively); Figure S9: The 5-fold cross-validation results of all RFS prediction models within 2 years after AR in the training cohort, where (a), (b), (c) and (d) represent the ROC curves of the proposed model, GBDT, RF and XGBoost, respectively (the curves on the left and right are the results of the models with radiomics and radiomics + clinical features as input features, respectively); Figure S10: The 5-fold cross-validation results of all RFS prediction models within 3 years after AR in the training cohort, where (a), (b), (c) and (d) represent the ROC curves of the proposed model, GBDT, RF and XGBoost, respectively (the curves on the left and right are the results of the models with radiomics and radiomics + clinical features as input features, respectively); Figure S11: The 5-fold cross-validation results of all RFS prediction models within 1 year after NAR in the training cohort, where (a), (b), (c) and (d) represent the ROC curves of the proposed model, GBDT, RF and XGBoost, respectively (the curves on the left and right are the results of the models with radiomics and radiomics + clinical features as input features, respectively); Figure S12: The 5-fold cross-validation results of all RFS prediction models within 2 years after NAR in the training cohort, where (a), (b), (c) and (d) represent the ROC curves of the proposed model, GBDT, RF and XGBoost, respectively (the curves on the left and right are the results of the models with radiomics and radiomics + clinical features as input features, respectively); Figure S13: The 5-fold cross-validation results of all RFS prediction models within 3 years after NAR in the training cohort, where (a), (b), (c) and (d) represent the ROC curves of the proposed model, GBDT, RF and XGBoost, respectively (the curves on the left and right are the results of the models with radiomics and radiomics + clinical features as input features, respectively); Table S1: The specific model architecture and core parameters of each model; Table S2: Clinical baseline features related to overall survival (OS) after surgery screened by univariate and multivariate Cox regression methods; Table S3: Clinical baseline features related to recurrence-free survival (RFS) after surgery screened by univariate and multivariate Cox regression methods; Table S4: Summary of screened radiomic features in each model, including their number and names
