# Supplementary material for: Data-Driven Assisted Decision Making for Surgical Procedure of Hepatocellular Carcinoma Resection and Prognostic Prediction: Development and Validation of Machine Learning Models
Source: Cancers (Basel). 2023 Mar 15;15(6):1784. doi: 10.3390/cancers15061784 (PMC10046511; doi:10.3390/cancers15061784)
Supplement: Supplementary file 1 [file cancers-15-01784-s001.zip › Figure caption.pdf]

Supplementary Materials

Figure S1 The visualization effect of automatic segmentation, which shows the comparison of Segformer, U-Net, U-Net++ and manual annotation.

Figure S2 The 5-fold cross-validation results of all models using the Least Absolute Shrinkage Selector Operator (LASSO) regression algorithm for feature screening.

Figure S3 The convergence process of all Least Absolute Shrinkage Selector Operator (LASSO) regression models, where (**a**), (**b**), and (**c**) are the model convergence results of decision-making, overall survival (OS) prediction, and recurrence-free survival (RFS) prediction, respectively.

Figure S4 The screening results of radiomics features in all models, where (**a**), (**b**) and (**c**) are the screening results of model features for decision-making, overall survival (OS) prediction and recurrence-free survival (RFS) prediction, respectively.

Figure S5 The 5-fold cross-validation results of all decision-making models in the training cohort, where (**a**), (**b**), (**c**) and (**d**) represent the receiver operating characteristic (ROC) curves of the proposed model, Gradient Boosting Decision Tree (GBDT), Random Forest (RF) and Extreme Gradient Boosting (XGBoost), respectively (the curves on the left and right are the results of the models with radiomics and radiomics+clinical features as input features, respectively).

Figure S6 The 5-fold cross-validation results of all OS prediction models within 3 years after anatomical liver resection (AR) in the training cohort, where (**a**), (**b**), (**c**) and (**d**) represent the ROC curves of the proposed model, GBDT, RF and XGBoost, respectively (the curves on the left and right are the results of the models with radiomics and radiomics+clinical features as input features, respectively).

Figure S7 The 5-fold cross-validation results of all OS prediction models within 3 years after non-anatomical liver resection (NAR) in the training cohort, where (**a**), (**b**), (**c**) and (**d**) represent the ROC curves of the proposed model, GBDT, RF and XGBoost, respectively (the curves on the left and right are the results of the models with radiomics and radiomics+clinical features as input features, respectively).

Figure S8 The 5-fold cross-validation results of all RFS prediction models within 1 year after AR in the training cohort, where (**a**), (**b**), (**c**) and (**d**) represent the ROC curves of the proposed model, GBDT, RF and XGBoost, respectively (the curves on the left and right are the results of the models with radiomics and radiomics+clinical features as input features, respectively).

Figure S9 The 5-fold cross-validation results of all RFS prediction models within 2 years after AR in the training cohort, where (**a**), (**b**), (**c**) and (**d**) represent the ROC curves of the proposed model, GBDT, RF and XGBoost, respectively (the curves on the left and right are the results of the models with radiomics and radiomics+clinical features as input features, respectively).

Figure S10 The 5-fold cross-validation results of all RFS prediction models within 3 years after AR in the training cohort, where (**a**), (**b**), (**c**) and (**d**) represent the ROC curves of the proposed model, GBDT, RF and XGBoost, respectively (the curves on the left and right are the results of the models with radiomics and radiomics+clinical features as input features, respectively).

Figure S11 The 5-fold cross-validation results of all RFS prediction models within 1 year after NAR in the training cohort, where (**a**), (**b**), (**c**) and (**d**) represent the ROC curves of the proposed model, GBDT, RF and XGBoost, respectively (the curves on the left and right are the results of the models with radiomics and radiomics+clinical features as input features, respectively).

Figure S12 The 5-fold cross-validation results of all RFS prediction models within 2 years after NAR in the training cohort, where (**a**), (**b**), (**c**) and (**d**) represent the ROC curves of the proposed model, GBDT, RF and XGBoost, respectively (the curves on the left and right are the results of the models with radiomics and radiomics+clinical features as input features, respectively).

Figure S13 The 5-fold cross-validation results of all RFS prediction models within 3 years after NAR in the training cohort, where (**a**), (**b**), (**c**) and (**d**) represent the ROC curves of the proposed model, GBDT, RF and XGBoost, respectively (the curves on the left and right are the results of the models with radiomics and radiomics+clinical features as input features, respectively).
