# Supplementary material for: Data-Driven Assisted Decision Making for Surgical Procedure of Hepatocellular Carcinoma Resection and Prognostic Prediction: Development and Validation of Machine Learning Models
Source: Cancers (Basel). 2023 Mar 15;15(6):1784. doi: 10.3390/cancers15061784 (PMC10046511; doi:10.3390/cancers15061784)
Supplement: Supplementary file 1 [file cancers-15-01784-s001.zip › Table S1.pdf]

Table S1 The specific model architecture and core parameters of each model

Model name	Model Structure	Key Parameters
DeepDMLR	Base Classifier:<br>DNN 1<br>DNN 2<br>DNN 3<br>Ensemble way:<br>Soft-voting	Activation function: ReLU.<br>Optimizer: Adam.<br>Initial learning rate: 0.0001
DeepSPLR	Base Classifier:<br>DNN<br>SVM<br>LR<br>Ensemble way:<br>Soft-voting	kernel function: RBF.<br>Penalty factor C: 0.1
DeepRPLR	Base Classifier:<br>DNN<br>LR<br>DT<br>Ensemble way:<br>Soft-voting	Max_depth: 80
