# Supplementary material for: Data-Driven Assisted Decision Making for Surgical Procedure of Hepatocellular Carcinoma Resection and Prognostic Prediction: Development and Validation of Machine Learning Models
Source: Cancers (Basel). 2023 Mar 15;15(6):1784. doi: 10.3390/cancers15061784 (PMC10046511; doi:10.3390/cancers15061784)
Supplement: Supplementary file 1 [file cancers-15-01784-s001.zip › Table S2.pdf]

Table S2 Clinical baseline features related to overall survival (OS) after surgery screened by univariate and multivariate Cox regression methods.

	Univariate analysis		Multi-variable analysis	
Characteristics	HR (95% CI)	P	HR (95% CI)	P
Sex		0.999		
Male	Reference			
Female	1.000 (0.482-2.075)	0.999		
Age		0.236		
＜60	Reference			
≥60	0.692 (0.376-1.273)	0.236	**0.635 (0.320-1.259)**	**0.194**
BMI	0.990 (0.898-1.091)	0.835		
Cirrhosis		0.428		
Yes	Reference			
No	0.789 (0.439-1.417)	0.428		
Hepatitis B		0.636		
Yes	Reference			
No	0.849 (0.430-1.675)	0.636		
Hepatitis C		0.449		
Yes	Reference			
No	0.578 (0.140-2.388)	0.449	**0.229 (0.044-1.187)**	**0.079**
Smoking		0.790		
Yes	Reference			
No	0.896 (0.400-2.007)	0.790		
Drinking		0.657		
Yes	Reference			
No	1.234 (0.487-3.128)	0.657		
BCLC grade		<0.001		
0	Reference			
A	4.848 (1.163-20.206)	0.03	**6.031 (1.409-25.815)**	**0.015**

B	13.561 (2.970-61.920)	<0.001	**15.976 (3.402-75.020)**	**<0.001**
TB	0.995 (0.950-1.043)	0.848	**0.969 (0.913-1.028)**	**0.295**
DB		0.242		
Normal	Reference			
Abnormal	1.484 (0.766-2.873)	0.242	**2.113 (0.899-4.968)**	**0.086**
ALB	0.947 (0.870-1.031)	0.207	0.960 (0.872-1.058)	0.414
ALT	1.000 (0.993-1.007)	0.940		
AST	1.003 (0.997-1.010)	0.335	1.002 (0.994-1.010)	0.596
GGT	1.001 (0.999-1.002)	0.460		
PA	0.026 (0.000-7.282)	0.204	0.073 (0.000-120.518)	0.488
Urea	0.904 (0.728-1.124)	0.364	1.013 (0.794-1.292)	0.917
Creatinine	0.998 (0.979-1.017)	0.815		
Uric acid	1.000 (0.995-1.004)	0.869		
PT	1.162 (0.964-1.402)	0.116	**0.826 (0.628-1.087)**	**0.173**
INR		<0.001		
Normal	Reference			
Abnormal	10.162 (4.277-24.142)	<0.001	**23.119 (6.502-82.201)**	**<0.001**
AFP	1.000 (1.000-1.000)	0.247	1.000 (1.000-1.000)	0.592
CEA	1.106 (0.964-1.270)	0.152	**1.113 (0.957-1.294)**	**0.167**
Hb	1.002 (0.985-1.020)	0.786		
WBC	0.979 (0.834-1.149)	0.793		
PLT	1.001 (0.998-1.004)	0.506	**1.003 (0.999-1.007)**	**0.109**

Note: BMI: Body Mass Index; BCLC: Barcelona Clinic Liver Cancer; TB: Total Bilirubin; DB: Direct Bilirubin; ALB: Albumin; ALT: Alanine Transaminase; AST: Aspartate Aminotransferase; GGT: γ-glutamyl Transferase; PA: Prealbumin; PT: Prothrombin Time; INR: International Normalized Ratio; AFP: Alpha Fetoprotein; CEA: Carcinoembryonic Antigen; Hb: Hemoglobin; WBC: White Blood Cell; PLT: Platelet Count. When P<0.4, the factor was included in the multivariate regression model used to screen for OS-related factors. It is important to emphasize that among the screening OS-related factors, although certain indicators did not reach critical values

(P values) in this dataset, they were still included because they have been generally agreed upon by previous clinical studies.
