# Supplementary material for: Data-Driven Assisted Decision Making for Surgical Procedure of Hepatocellular Carcinoma Resection and Prognostic Prediction: Development and Validation of Machine Learning Models
Source: Cancers (Basel). 2023 Mar 15;15(6):1784. doi: 10.3390/cancers15061784 (PMC10046511; doi:10.3390/cancers15061784)
Supplement: Supplementary file 1 [file cancers-15-01784-s001.zip › Table S3.pdf]

Table S3 Clinical baseline features related to recurrence-free survival (RFS) after surgery screened by univariate and multivariate Cox regression methods.

	Univariate analysis		Multi-variable analysis	
Characteristics	HR(95% CI)	P	HR(95% CI)	P
Sex		0.979		
Male	Reference			
Female	1.005 (0.666-1.517)	0.979		
Age		0.566		
＜60	Reference			
≥60	0.904 (0.642-1.275)	0.566		
BMI	0.956 (0.903-1.011)	0.115	**0.962 (0.908-1.020)**	**0.197**
Cirrhosis		0.123		
Yes	Reference			
No	0.765 (0.545-1.075)	0.123	**0.784 (0.548-1.124)**	**0.185**
Hepatitis B		0.831		
Yes	Reference			
No	0.959 (0.652-1.410)	0.831		
Hepatitis C		0.317		
Yes	Reference			
No	0.633 (0.259-1.549)	0.317		
Smoking		0.668		
Yes	Reference			
No	0.901 (0.560-1.450)	0.668		
Drinking		0.826		
Yes	Reference			
No	0.946 (0.576-1.554)	0.826		
BCLC grade		<0.001		
0	Reference			
A	1.985 (1.170-3.368)	0.011	**2.285 (1.328-3.934)**	**0.003**

B	5.010 (2.591-9.686)	<0.001	**5.234 (2.662-10.291)**	**<0.001**
TB	1.028 (1.004-1.052)	0.022	1.008 (0.974-1.044)	0.643
DB		0.01		
Normal	Reference			
Abnormal	1.642 (1.123-2.400)	0.01	**1.438 (0.864-2.393)**	**0.162**
ALB	0.949 (0.903-0.997)	0.038	**0.959 (0.909-1.011)**	**0.123**
ALT	1.003 (0.999-1.006)	0.123	1.002 (0.997-1.006)	0.454
AST	1.005 (1.001-1.009)	0.007	**1.004 (1.000-1.009)**	**0.071**
GGT	1.001 (1.000-1.002)	0.002	**1.001 (1.000-1.002)**	**0.005**
PA	1.002 (0.994-1.011)	0.569		
Urea	0.955 (0.846-1.079)	0.463		
Creatinine	1.001 (0.991-1.011)	0.871		
Uric acid	0.999 (0.996-1.001)	0.244		
PT	1.155 (1.040-1.281)	0.007	**1.146 (0.987-1.330)**	**0.074**
INR		0.003		
Normal	Reference			
Abnormal	2.815 (1.425-5.559)	0.003	**1.831 (0.790-4.242)**	**0.159**
AFP	1.000 (1.000-1.000)	0.007	**1.000 (1.000-1.000)**	**0.045**
CEA	1.034 (0.942-1.135)	0.479		
Hb	0.997 (0.987-1.006)	0.49		
WBC	0.912 (0.828-1.005)	0.063	**0.885 (0.787-0.995)**	**0.04**
PLT	0.998 (0.996-1.001)	0.255		

Note: BMI: Body Mass Index; BCLC: Barcelona Clinic Liver Cancer; TB: Total Bilirubin; DB: Direct Bilirubin; ALB: Albumin; ALT: Alanine Transaminase; AST: Aspartate Aminotransferase; GGT: γ-glutamyl Transferase; PA: Prealbumin; PT: Prothrombin Time; INR: International Normalized Ratio; AFP: Alpha Fetoprotein; CEA: Carcinoembryonic Antigen; Hb: Hemoglobin; WBC: White Blood Cell; PLT: Platelet Count. When $P<0.2$, the factor was included in the multivariate regression model used to screen for RFS-related factors.
