# Supplementary material for: Data-Driven Assisted Decision Making for Surgical Procedure of Hepatocellular Carcinoma Resection and Prognostic Prediction: Development and Validation of Machine Learning Models
Source: Cancers (Basel). 2023 Mar 15;15(6):1784. doi: 10.3390/cancers15061784 (PMC10046511; doi:10.3390/cancers15061784)
Supplement: Supplementary file 1 [file cancers-15-01784-s001.zip › Table S4.pdf]

Table S4 Summary of screened radiomic features in each model, including their number and names.

Model	Radiomics Feature number	Feature names
DeepDMLR	6	original_shape_LeastAxisLength<br>original_shape_Maximum2DDiameterColumn<br>wavelet.LHL_glrlm_RunLengthNonUniformity<br>wavelet.HHL_glszm_SizeZoneNonUniformity<br>wavelet.HHH_glszm_SizeZoneNonUniformity<br>wavelet.LLL_gldm_DependenceNonUniformity
DeepSPLR-AR	7	original_shape_Elongation<br>wavelet.LHL_gldm_SmallDependenceHighGrayLevelEmphasis<br>wavelet.HLH_firstorder_InterquartileRange<br>wavelet.HLH_firstorder_Skewness<br>wavelet.HLH_firstorder_Uniformity<br>wavelet.HHL_glszm_LargeAreaHighGrayLevelEmphasis<br>wavelet.LLL_glszm_LargeAreaLowGrayLevelEmphasis
DeepSPLR-NAR	12	wavelet.LLH_firstorder_Mean<br>wavelet.LLH_firstorder_Median<br>wavelet.HLH_glszm_LowGrayLevelZoneEmphasis<br>wavelet.HHL_glszm_LargeAreaLowGrayLevelEmphasis<br>wavelet.HHL_ngtdm_Busyness<br>wavelet.LLL_firstorder_Minimum<br>wavelet.LLL_glszm_GrayLevelVariance<br>squareroot_ngtdm_Busyness<br>squareroot_ngtdm_Strength<br>logarithm_firstorder_Kurtosis<br>logarithm_gldm_SmallDependenceHighGrayLevelEmphasis<br>exponential_firstorder_90Percentile
DeepRPLR-AR-1	33	original_shape_Elongation<br>original_shape_Flatness<br>wavelet.LLH_glrlm_LongRunHighGrayLevelEmphasis<br>wavelet.LLH_glszm_LargeAreaHighGrayLevelEmphasis

		wavelet.LHL_firstorder_Minimum
		wavelet.LHH_firstorder_Skewness
		wavelet.HLL_firstorder_Minimum
		wavelet.HLL_glrlm_ShortRunHighGrayLevelEmphasis
		wavelet.HLL_glszm_LargeAreaHighGrayLevelEmphasis
		wavelet.HLL_gldm_DependenceVariance
		wavelet.HLH_firstorder_Uniformity
		wavelet.HLH_glrlm_LongRunLowGrayLevelEmphasis
		wavelet.HLH_glszm_SmallAreaLowGrayLevelEmphasis
		wavelet.HHL_glrlm_LongRunLowGrayLevelEmphasis
		wavelet.HHL_glszm_SizeZoneNonUniformityNormalized
		wavelet.HHL_gldm_GrayLevelVariance
		wavelet.HHL_ngtdm_Busyness
		wavelet.HHL_ngtdm_Contrast
		wavelet.HHH_glrlm_GrayLevelVariance
		wavelet.HHH_glszm_GrayLevelNonUniformityNormalized
		wavelet.HHH_glszm_ZonePercentage
		wavelet.HHH_gldm_SmallDependenceHighGrayLevelEmphasis
		wavelet.LLL_ngtdm_Strength
		square_glszm_GrayLevelVariance
		square_gldm_DependenceNonUniformity
		squareroot_firstorder_Kurtosis
		squareroot_firstorder_Skewness
		squareroot_gldm_DependenceVariance
		squareroot_gldm_LargeDependenceHighGrayLevelEmphasis
		logarithm_glszm_SmallAreaEmphasis
		logarithm_ngtdm_Contrast
		logarithm_ngtdm_Strength
		gradient_gldm_DependenceNonUniformity
DeepRPLR-AR-2	41	original_shape_Elongation
		original_shape_Flatness
		original_glszm_SmallAreaLowGrayLevelEmphasis
		wavelet.LLH_glrlm_LongRunHighGrayLevelEmphasis
		wavelet.LLH_glszm_LargeAreaHighGrayLevelEmphasis

wavelet.LLH_gldm_DependenceNonUniformityNormalized
wavelet.LHL_firstorder_Skewness
wavelet.LHH_firstorder_Median
wavelet.LHH_firstorder_Skewness
wavelet.LHH_glszm_GrayLevelNonUniformityNormalized
wavelet.LHH_glszm_SmallAreaEmphasis
wavelet.LHH_ngtdm_Busyness
wavelet.HLL_glrlm_LongRunHighGrayLevelEmphasis
wavelet.HLL_ngtdm_Busyness
wavelet.HLH_firstorder_Uniformity
wavelet.HLH_gldm_LargeDependenceLowGrayLevelEmphasis
wavelet.HLH_gldm_SmallDependenceHighGrayLevelEmphasis
wavelet.HHL_glrlm_LongRunLowGrayLevelEmphasis
wavelet.HHL_glszm_GrayLevelNonUniformityNormalized
wavelet.HHL_glszm_ZoneEntropy
wavelet.HHL_glszm_ZonePercentage
wavelet.HHL_gldm_GrayLevelVariance
wavelet.HHL_ngtdm_Busyness
wavelet.HHH_glrlm_GrayLevelVariance
wavelet.HHH_glszm_GrayLevelNonUniformityNormalized
wavelet.HHH_gldm_DependenceVariance
wavelet.LLL_glszm_SmallAreaLowGrayLevelEmphasis
square_glszm_GrayLevelVariance
square_glszm_SmallAreaEmphasis
squareroot_glszm_SmallAreaEmphasis
squareroot_glszm_SmallAreaLowGrayLevelEmphasis
squareroot_gldm_DependenceVariance
logarithm_ngtdm_Busyness
logarithm_ngtdm_Contrast
logarithm_ngtdm_Strength
exponential_glrlm_LongRunEmphasis
exponential_glrlm_RunVariance
exponential_glszm_SmallAreaEmphasis
exponential_ngtdm_Complexity

		exponential_ngtdm_Strength<br>gradient_gldm_DependenceNonUniformity
DeepRPLR-AR-3	23	original_shape_Elongation<br>original_shape_Flatness<br>original_shape_MajorAxisLength<br>original_shape_Maximum2DDiameterSlice<br>original_gldm_DependenceNonUniformity<br>wavelet.LHH_firstorder_Kurtosis<br>wavelet.LHH_glszm_SmallAreaEmphasis<br>wavelet.HLL_glrlm_LongRunHighGrayLevelEmphasis<br>wavelet.HLL_glrlm_RunLengthNonUniformity<br>wavelet.HLH_firstorder_Skewness<br>wavelet.HHL_glszm_ZonePercentage<br>wavelet.HHL_ngtdm_Busyness<br>wavelet.HHL_ngtdm_Contrast<br>wavelet.HHH_gldm_DependenceVariance<br>wavelet.LLL_firstorder_Median<br>wavelet.LLL_ngtdm_Strength<br>square_gldm_GrayLevelNonUniformity<br>squareroot_glszm_SmallAreaLowGrayLevelEmphasis<br>squareroot_gldm_DependenceNonUniformity<br>logarithm_ngtdm_Busyness<br>logarithm_ngtdm_Contrast<br>exponential_glrlm_ShortRunLowGrayLevelEmphasis<br>exponential_glszm_SmallAreaEmphasis
DeepRPLR-NAR-1	9	original_shape_Sphericity<br>wavelet.HLL_glszm_SizeZoneNonUniformityNormalized<br>wavelet.HHL_glrlm_RunVariance<br>wavelet.HHL_ngtdm_Busyness<br>wavelet.HHL_ngtdm_Strength<br>wavelet.HHH_glrlm_RunVariance<br>wavelet.LLL_glrlm_GrayLevelNonUniformity<br>squareroot_ngtdm_Busyness<br>logarithm_glszm_ZoneVariance

DeepRPLR-NAR-2	13	original_shape_Sphericity<br>wavelet.LHL_glszm_LargeAreaLowGrayLevelEmphasis<br>wavelet.HLL_glszm_SizeZoneNonUniformityNormalized<br>wavelet.HLH_ngtdm_Busyness<br>wavelet.HHL_glrlm_RunVariance<br>wavelet.HHL_gldm_SmallDependenceEmphasis<br>wavelet.HHL_gldm_SmallDependenceHighGrayLevelEmphasis<br>wavelet.HHL_ngtdm_Busyness<br>wavelet.HHL_ngtdm_Strength<br>wavelet.HHH_glrlm_RunVariance<br>square_glrlm_GrayLevelNonUniformity<br>logarithm_glszm_ZoneVariance<br>gradient_glszm_SmallAreaLowGrayLevelEmphasis
DeepRPLR-NAR-3	6	wavelet.LHH_glrlm_ShortRunEmphasis<br>wavelet.HLH_ngtdm_Busyness<br>wavelet.HHL_ngtdm_Busyness<br>wavelet.HHH_glrlm_RunVariance<br>wavelet.LLL_glrlm_GrayLevelNonUniformity<br>square_glrlm_GrayLevelNonUniformity
