# Supplementary figures and images for: Data-Driven Assisted Decision Making for Surgical Procedure of Hepatocellular Carcinoma Resection and Prognostic Prediction: Development and Validation of Machine Learning Models
Source: Cancers (Basel). 2023 Mar 15;15(6):1784. doi: 10.3390/cancers15061784 (PMC10046511; doi:10.3390/cancers15061784)

**a**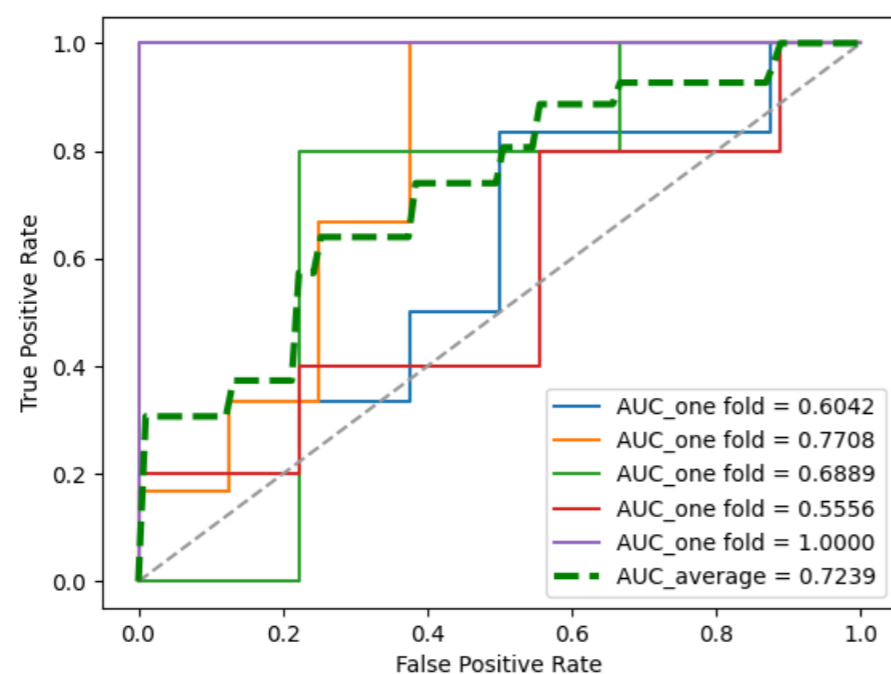**b**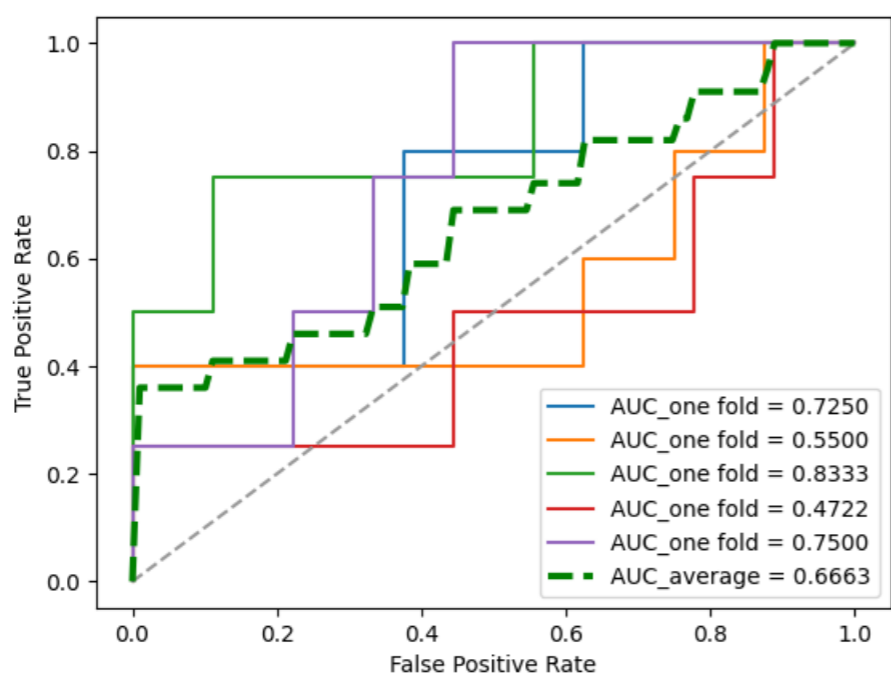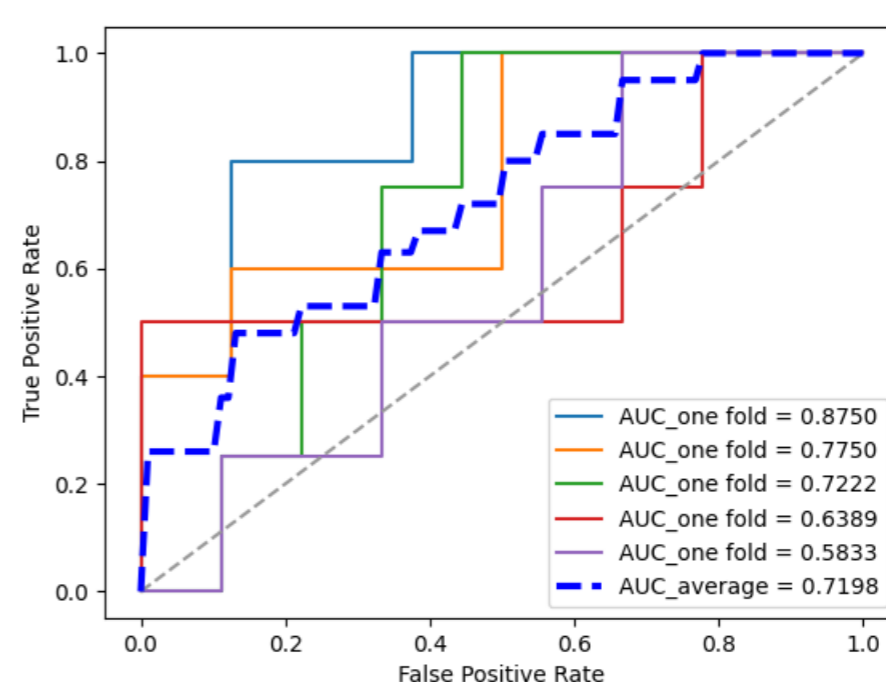**c**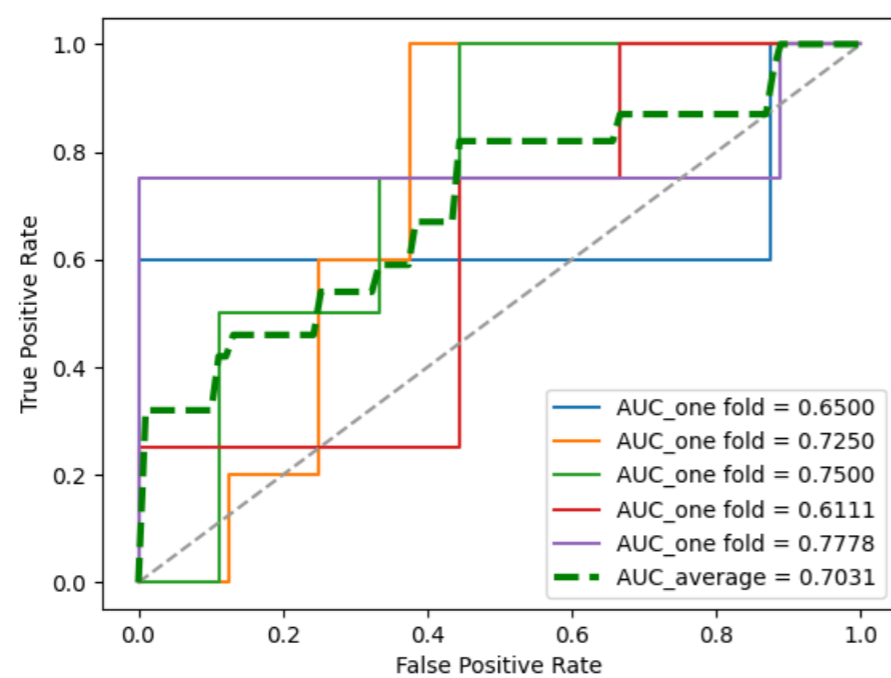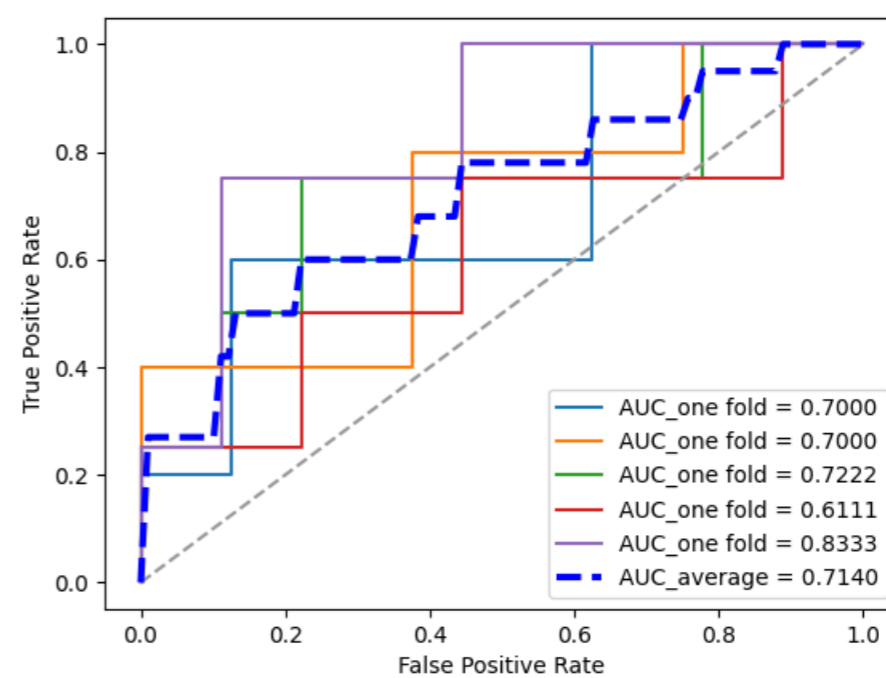**d**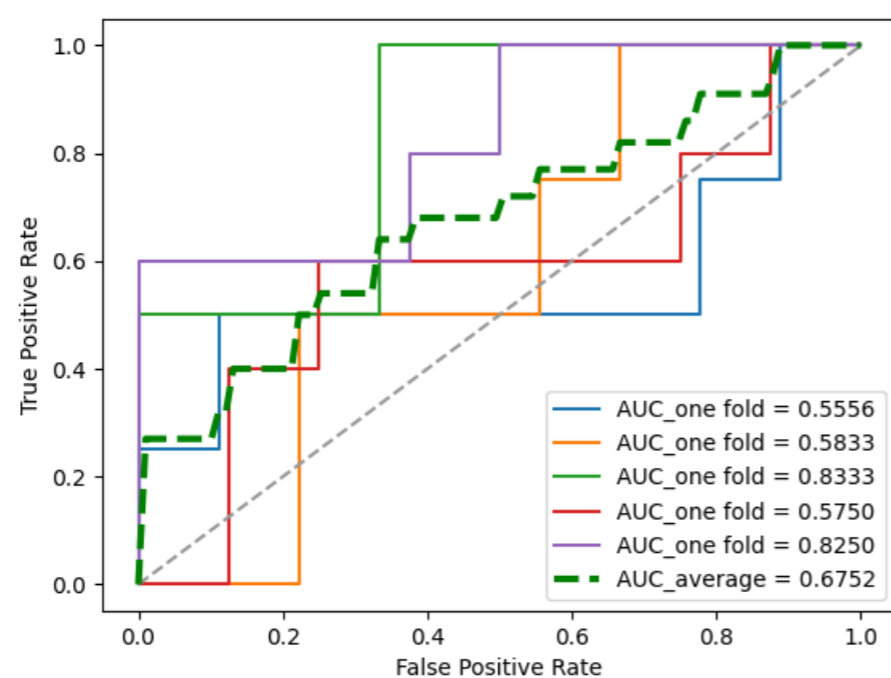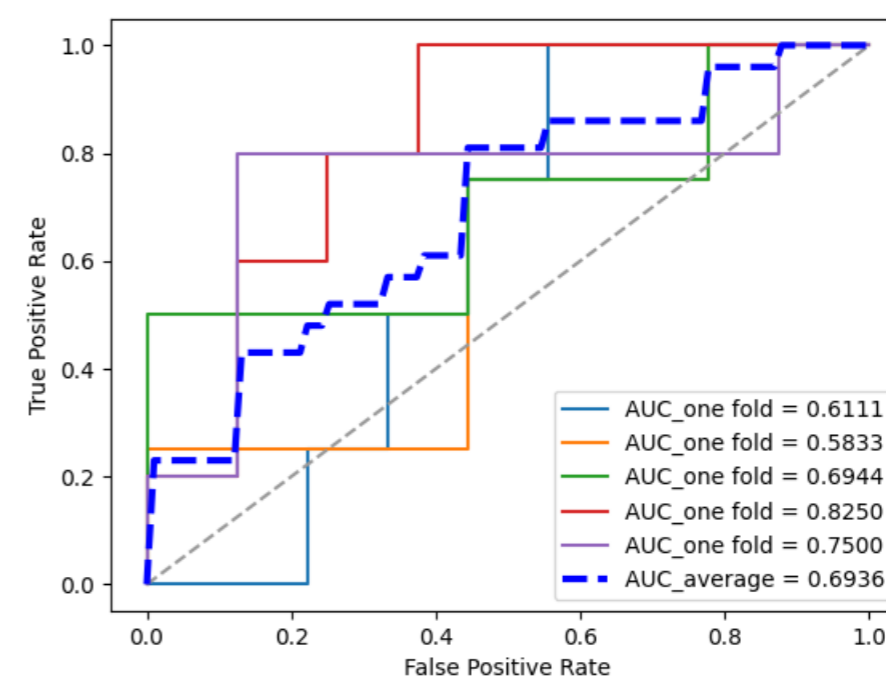

Supplement: Supplementary file 1 [file cancers-15-01784-s001.zip › Figure S10.pdf]

**a**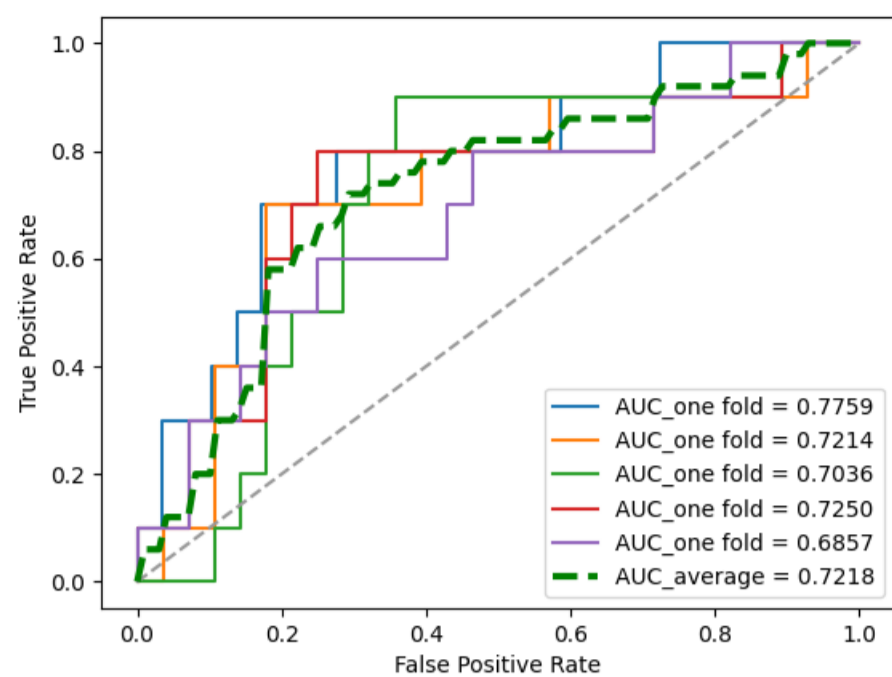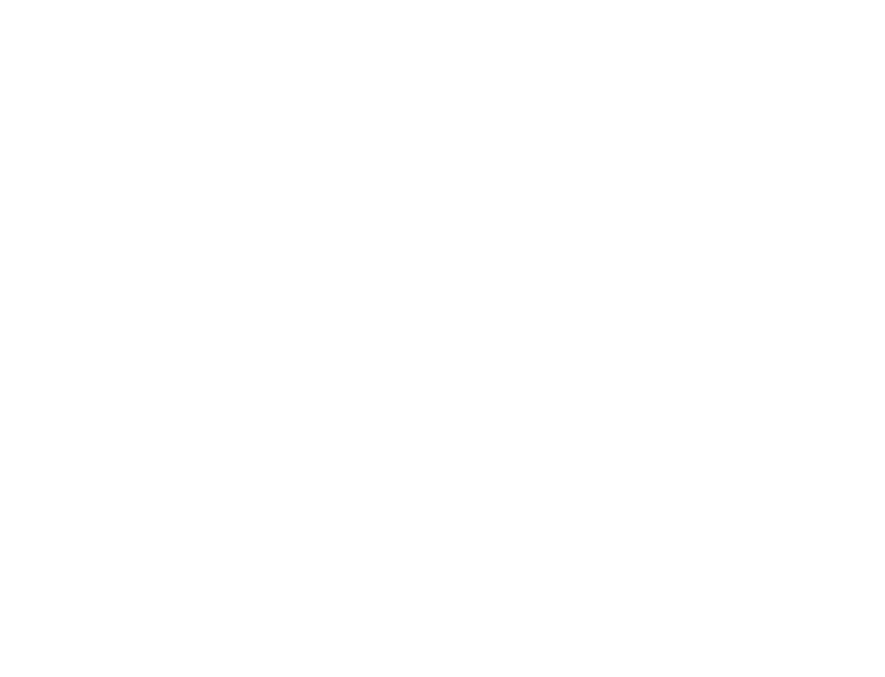**b**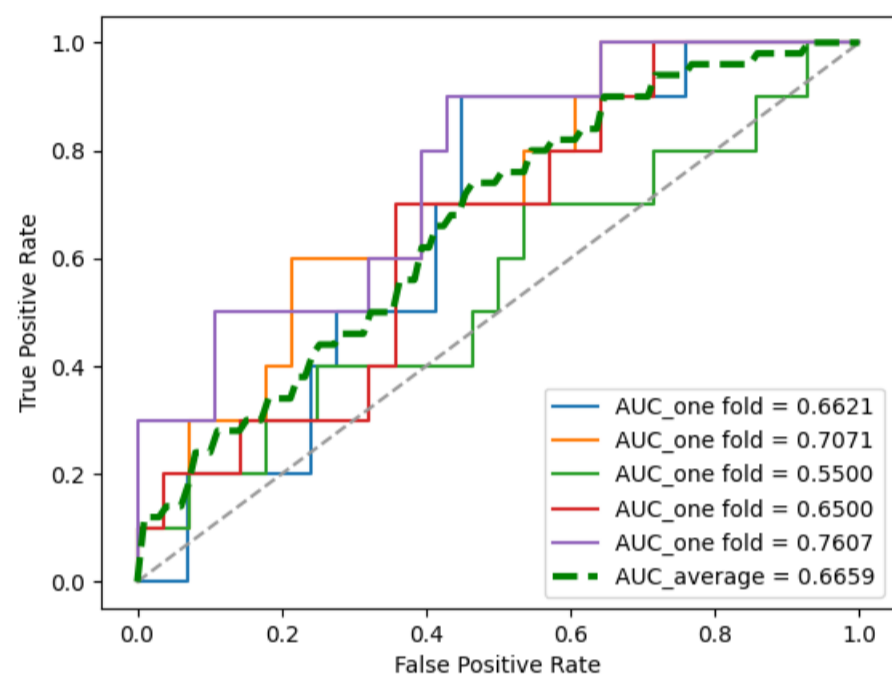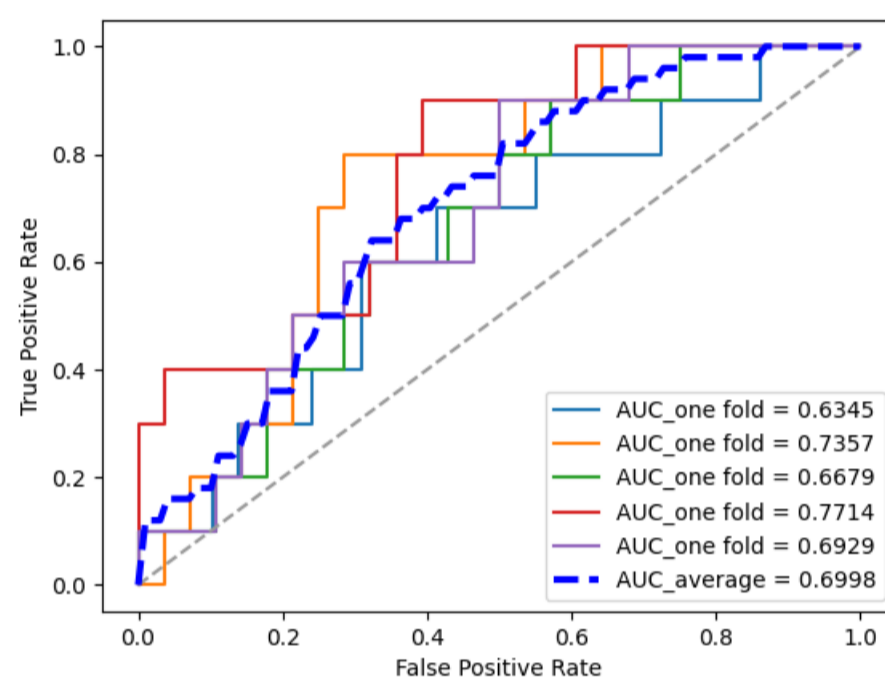**c**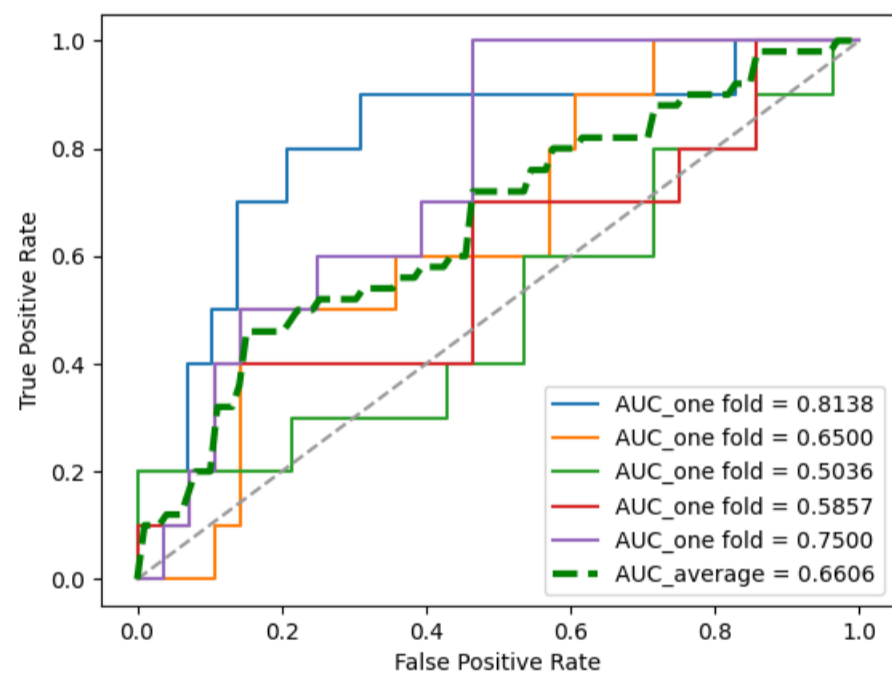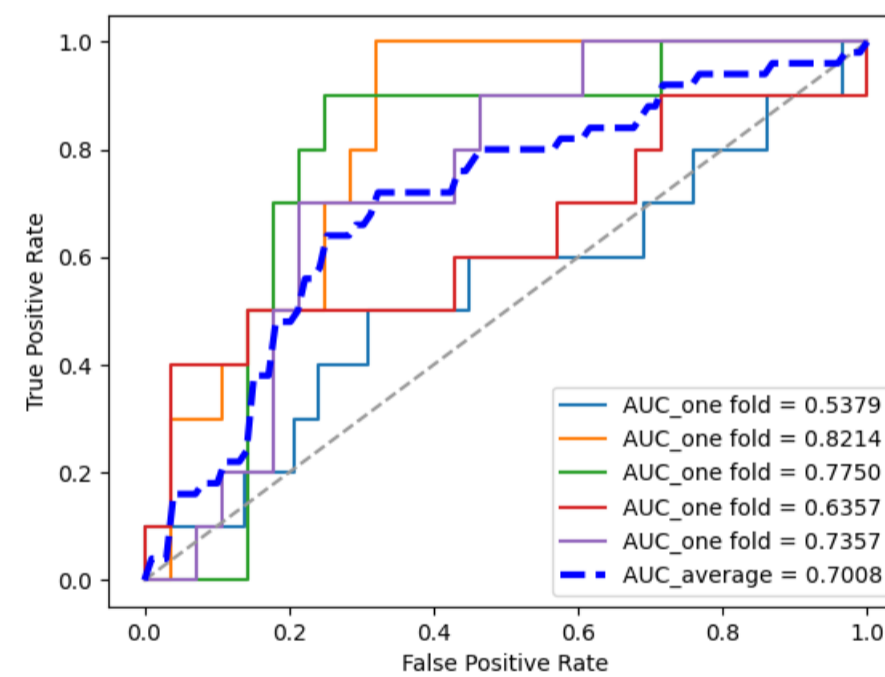**d**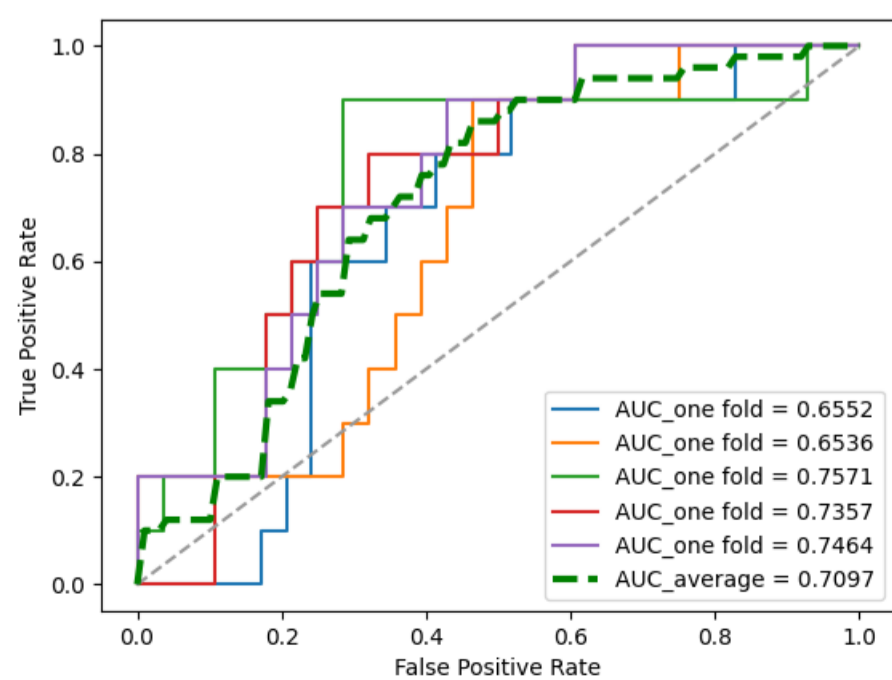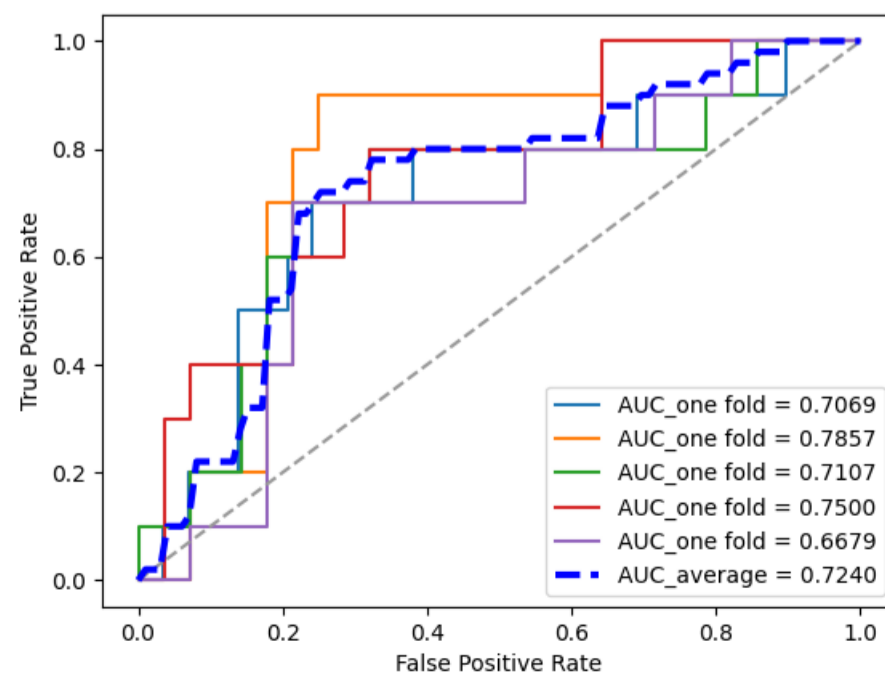

Supplement: Supplementary file 1 [file cancers-15-01784-s001.zip › Figure S11.pdf]

**a**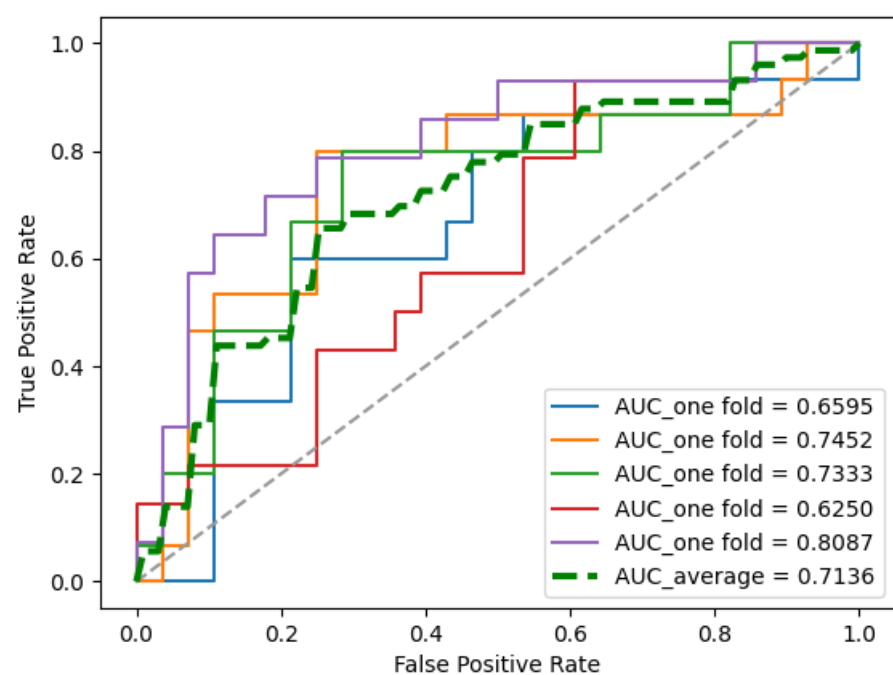**b**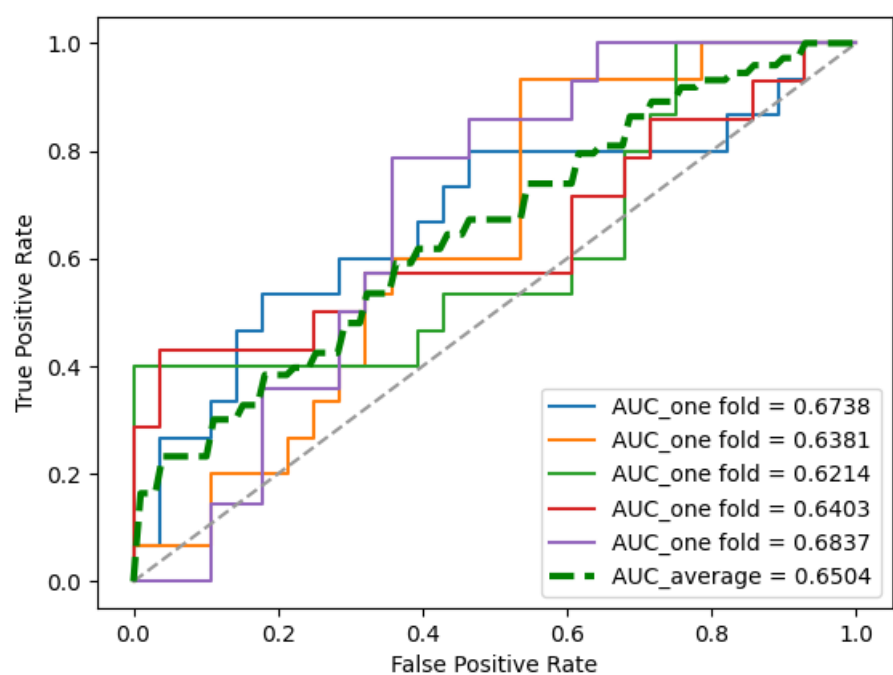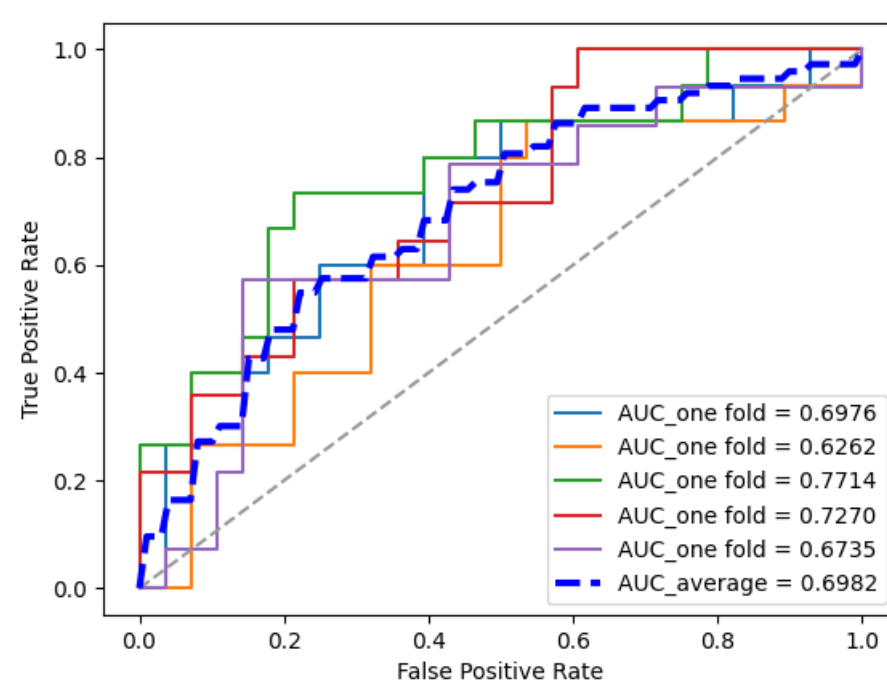**c**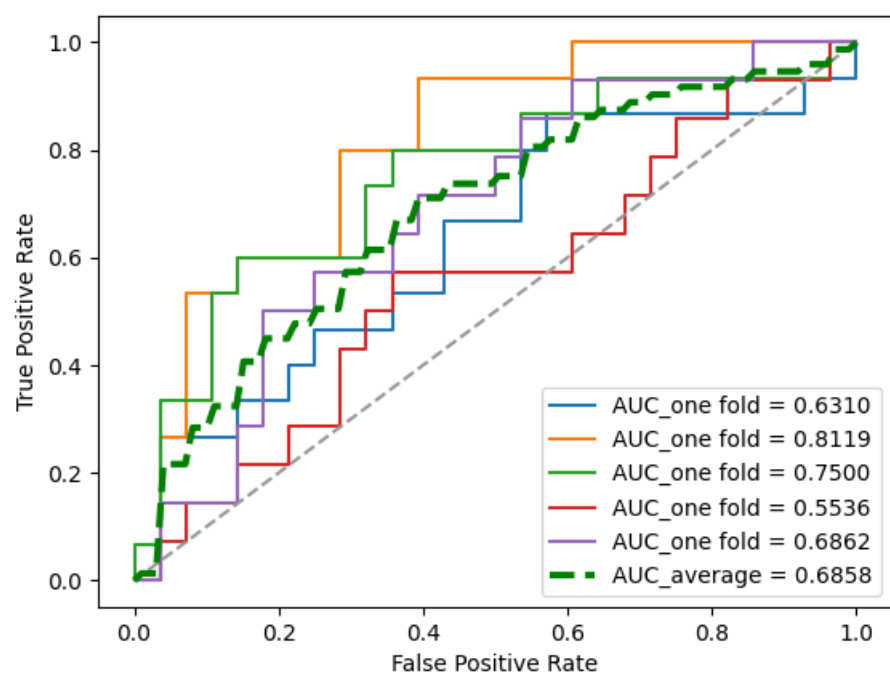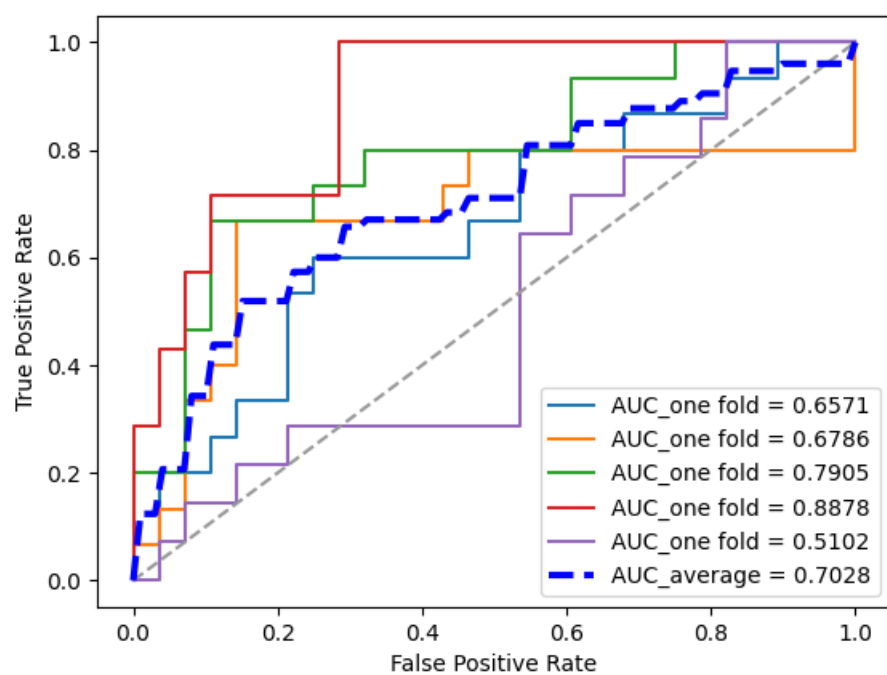**d**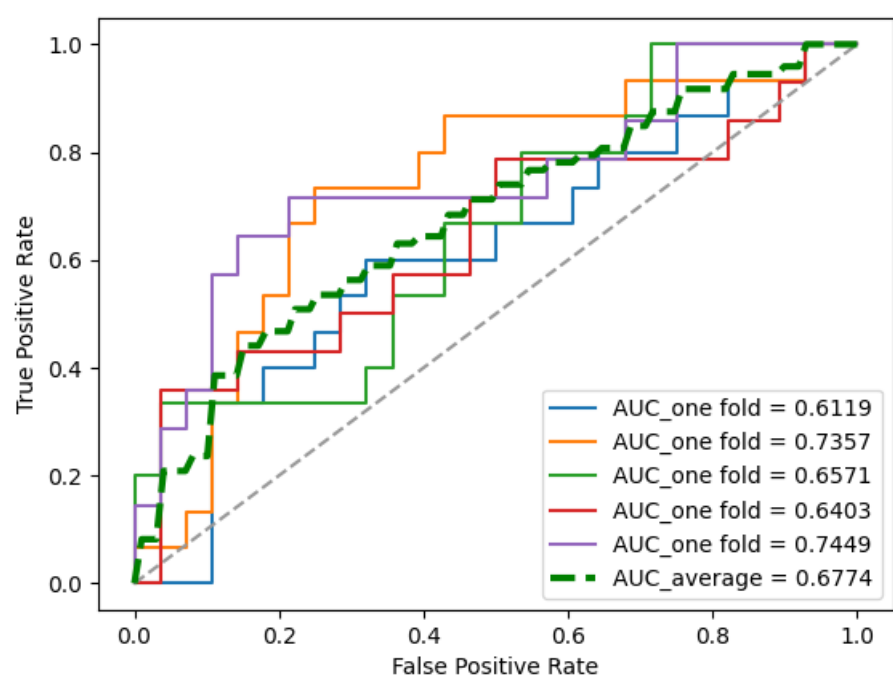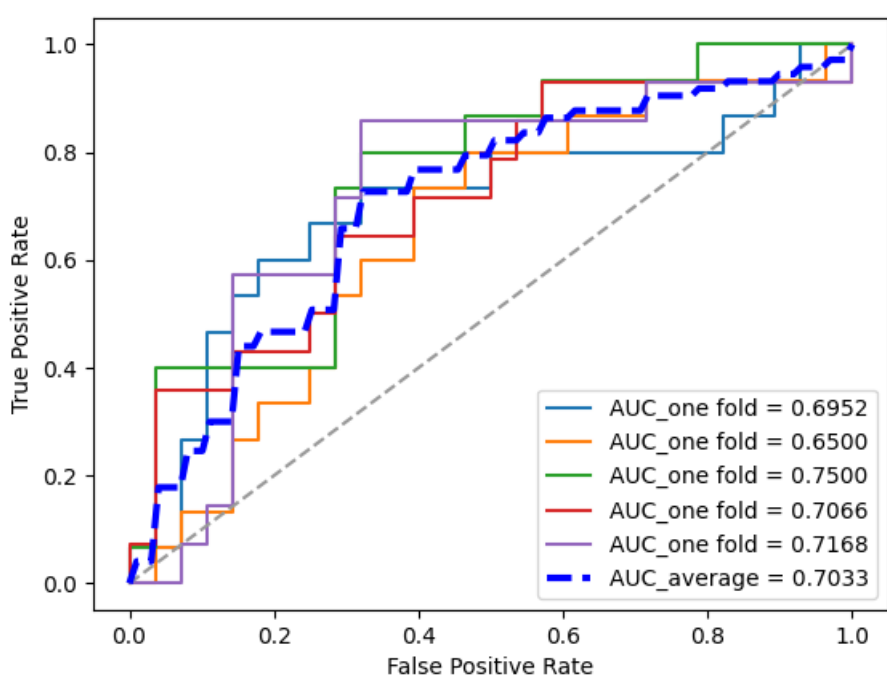

Supplement: Supplementary file 1 [file cancers-15-01784-s001.zip › Figure S12.pdf]

**a**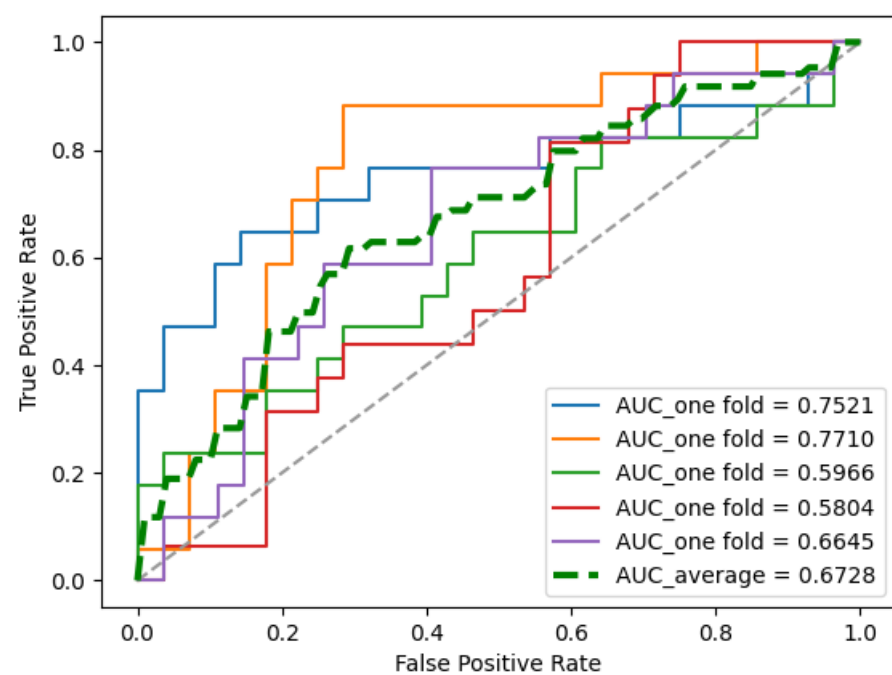**b**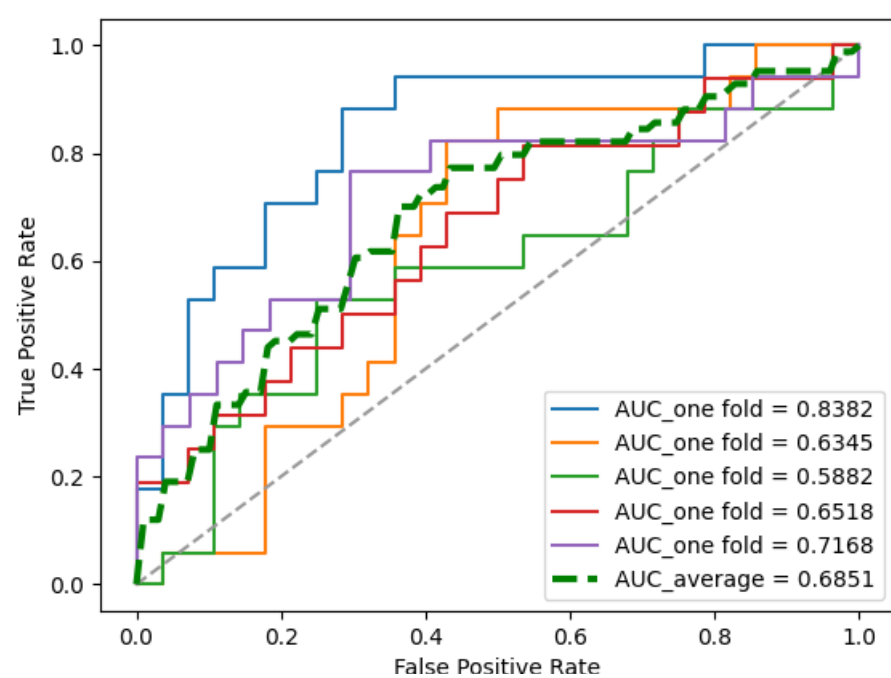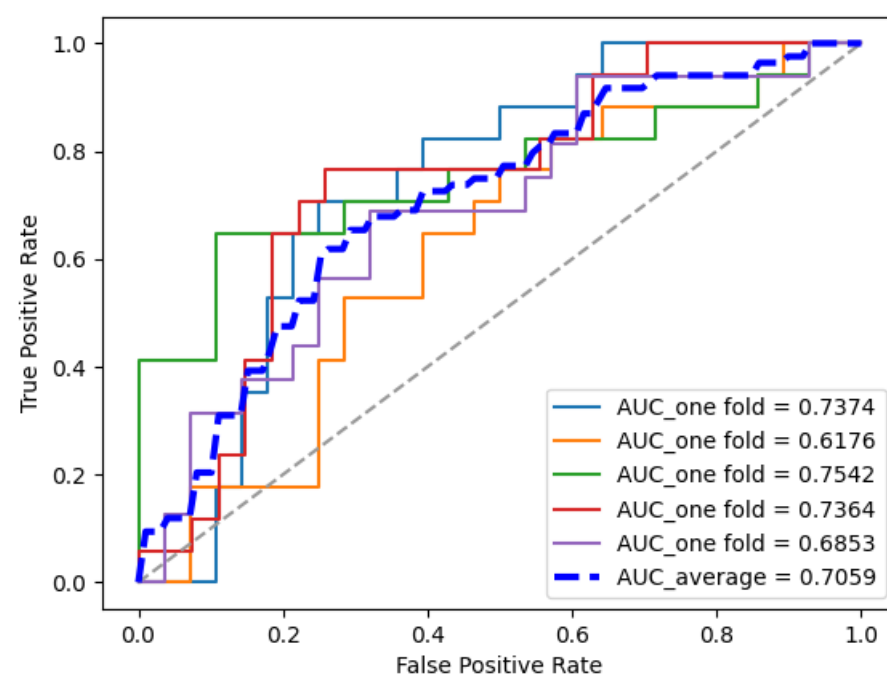**c**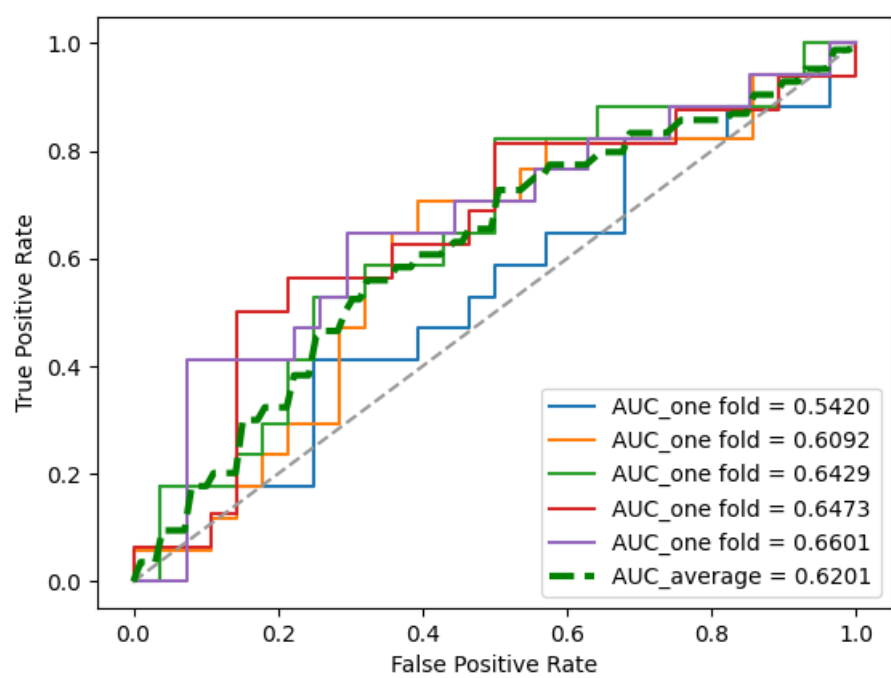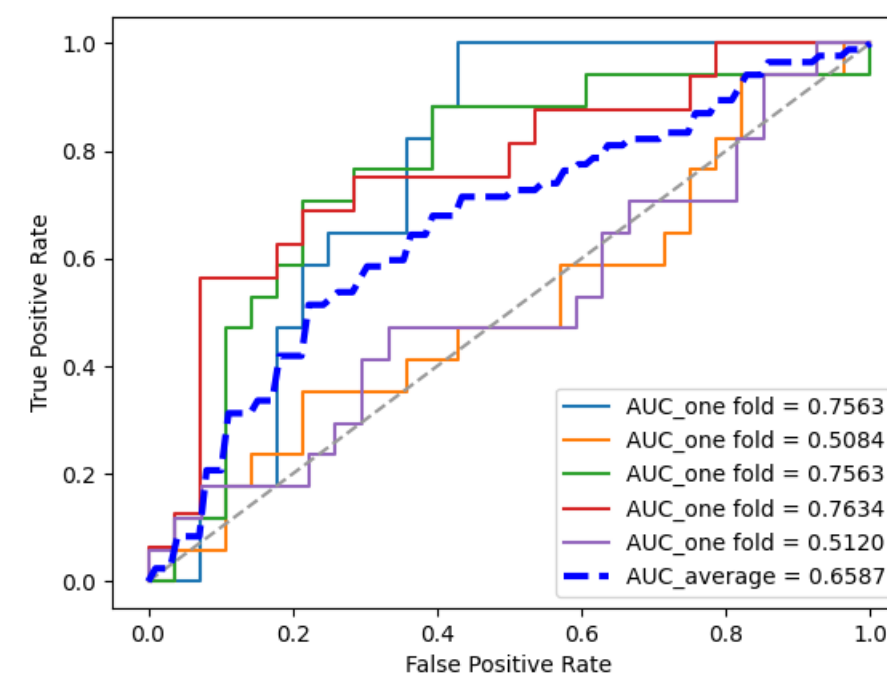**d**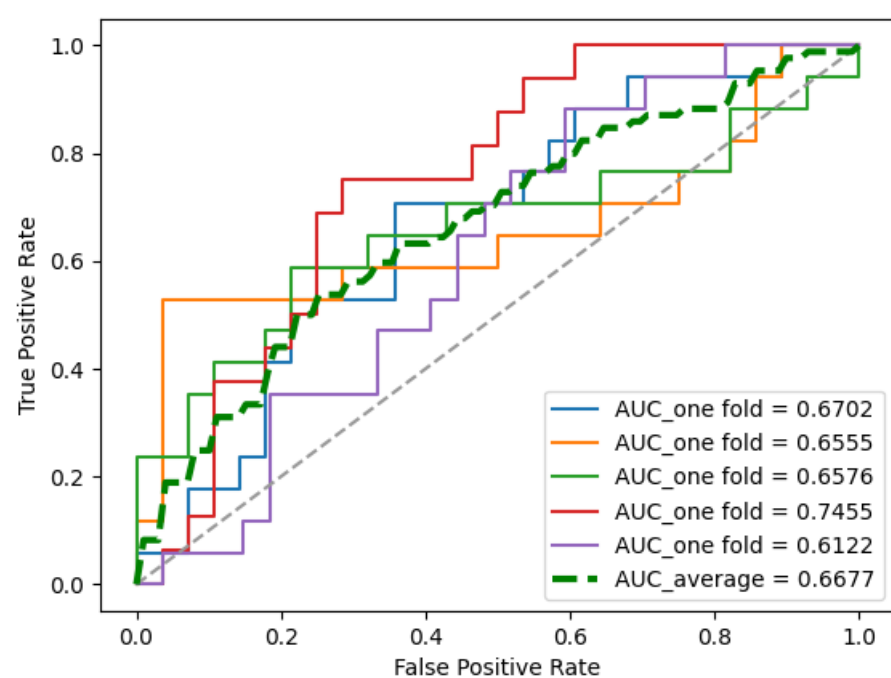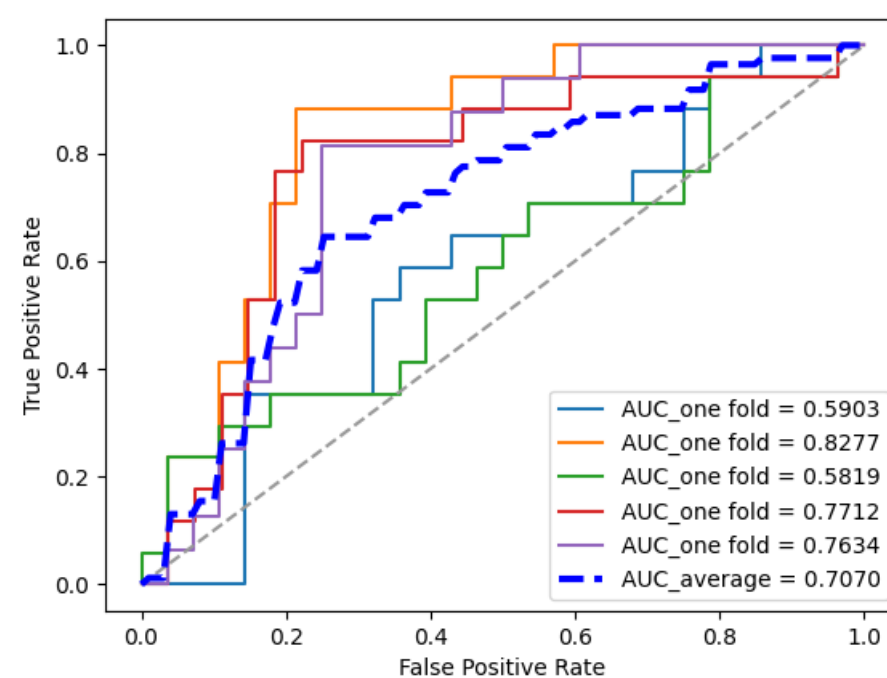

Supplement: Supplementary file 1 [file cancers-15-01784-s001.zip › Figure S13.pdf]

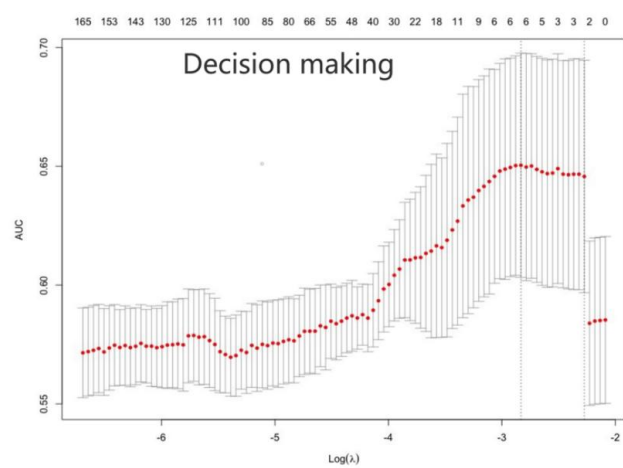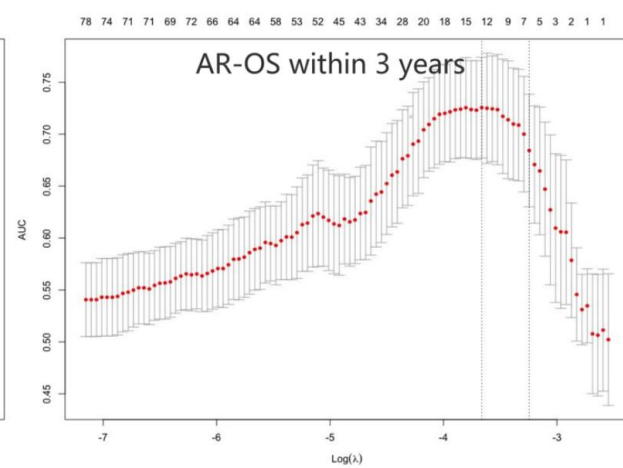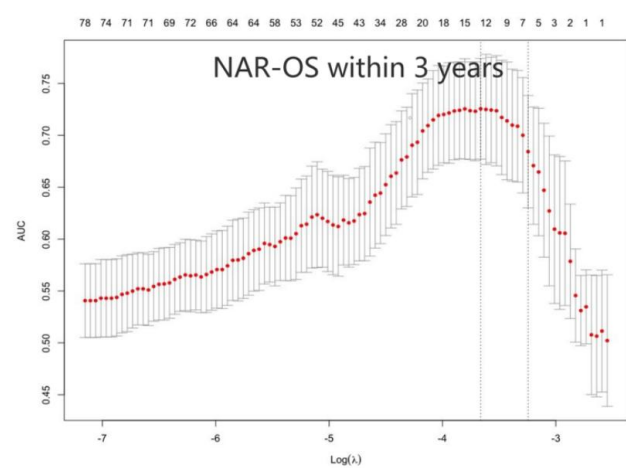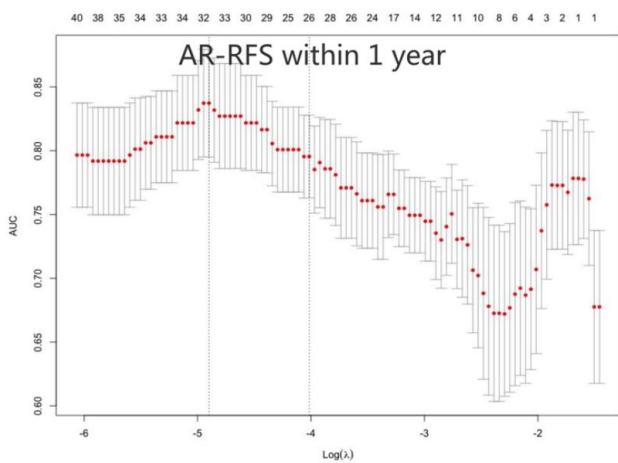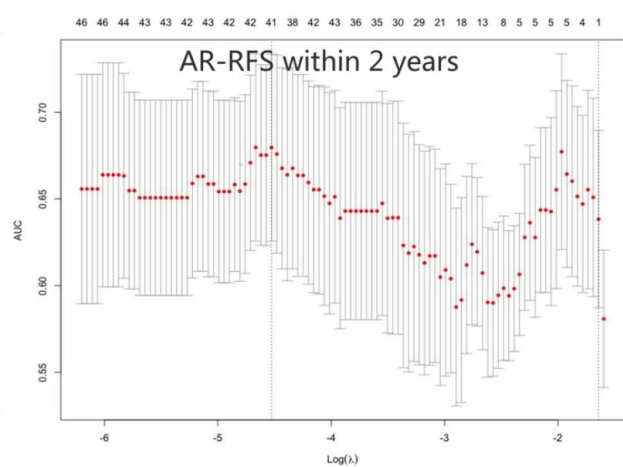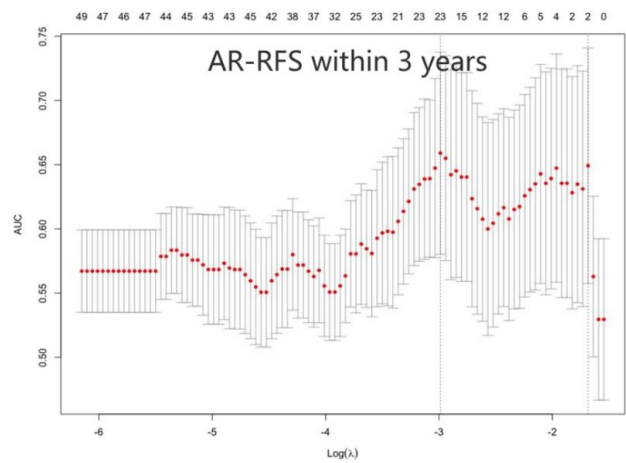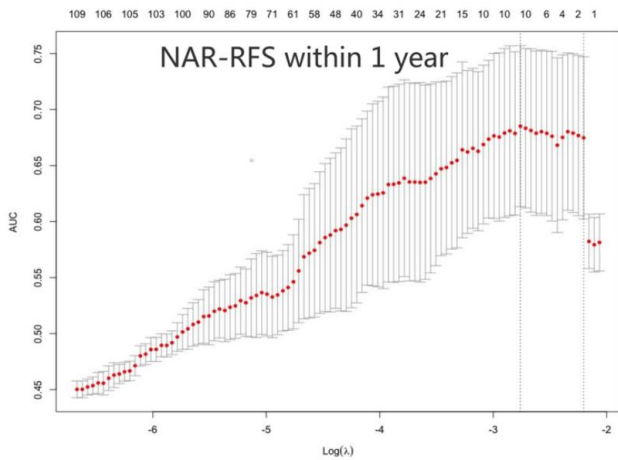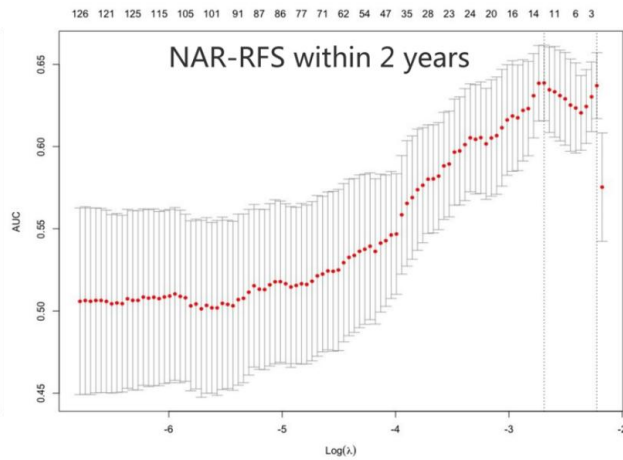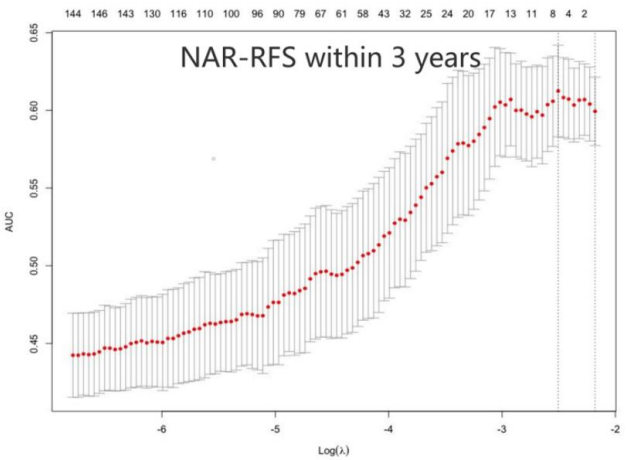

Supplement: Supplementary file 1 [file cancers-15-01784-s001.zip › Figure S2.pdf]

**a**

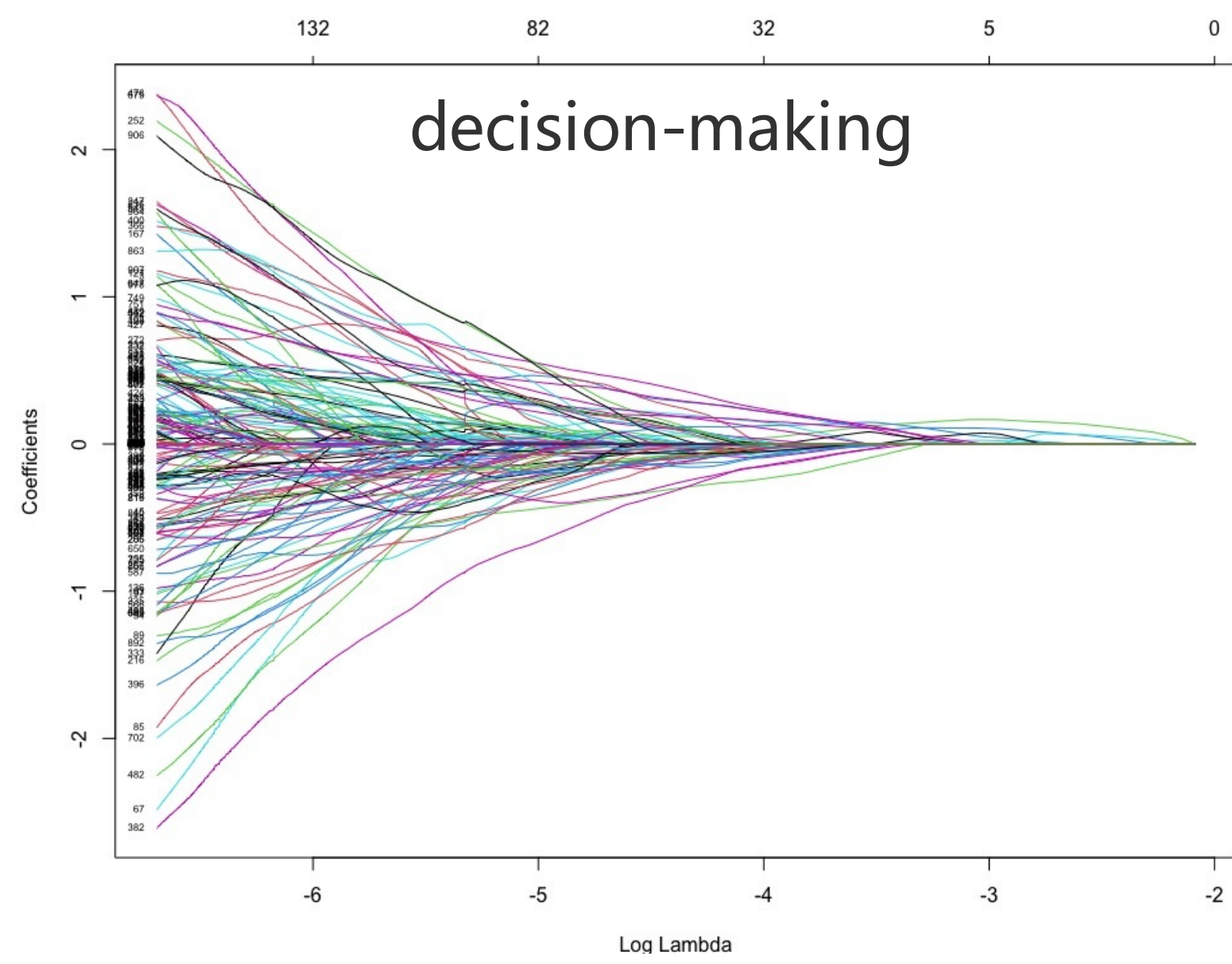

b

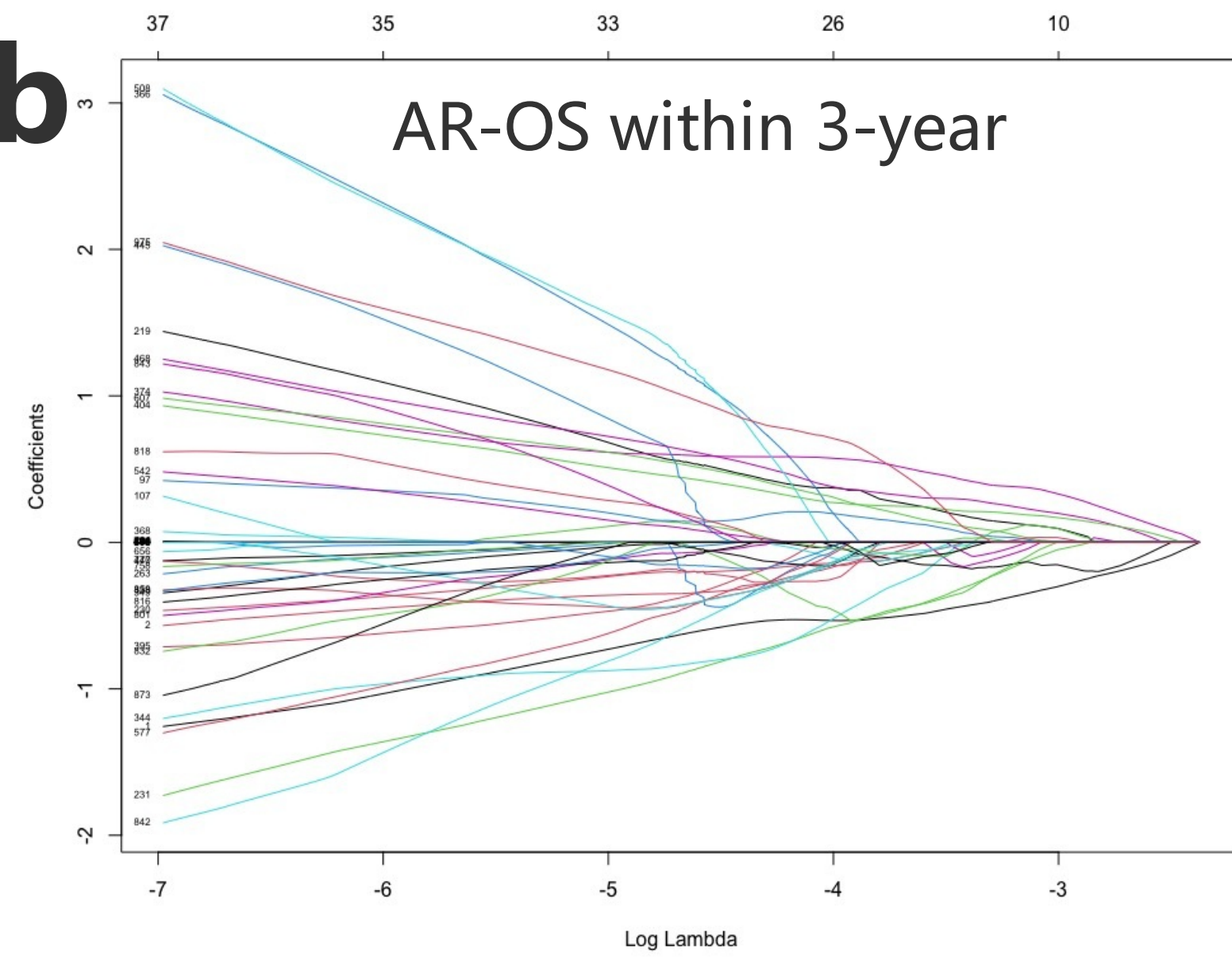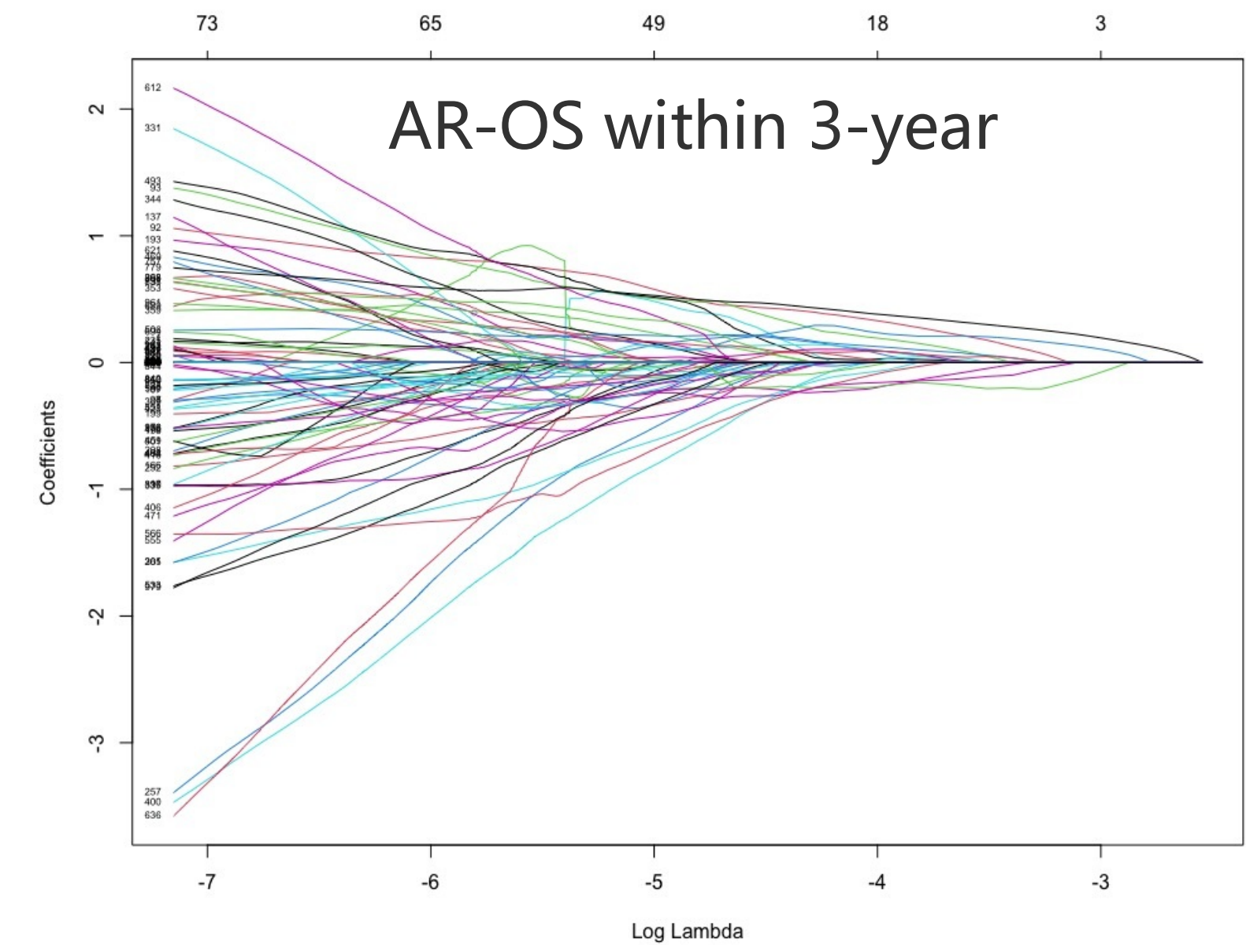

C

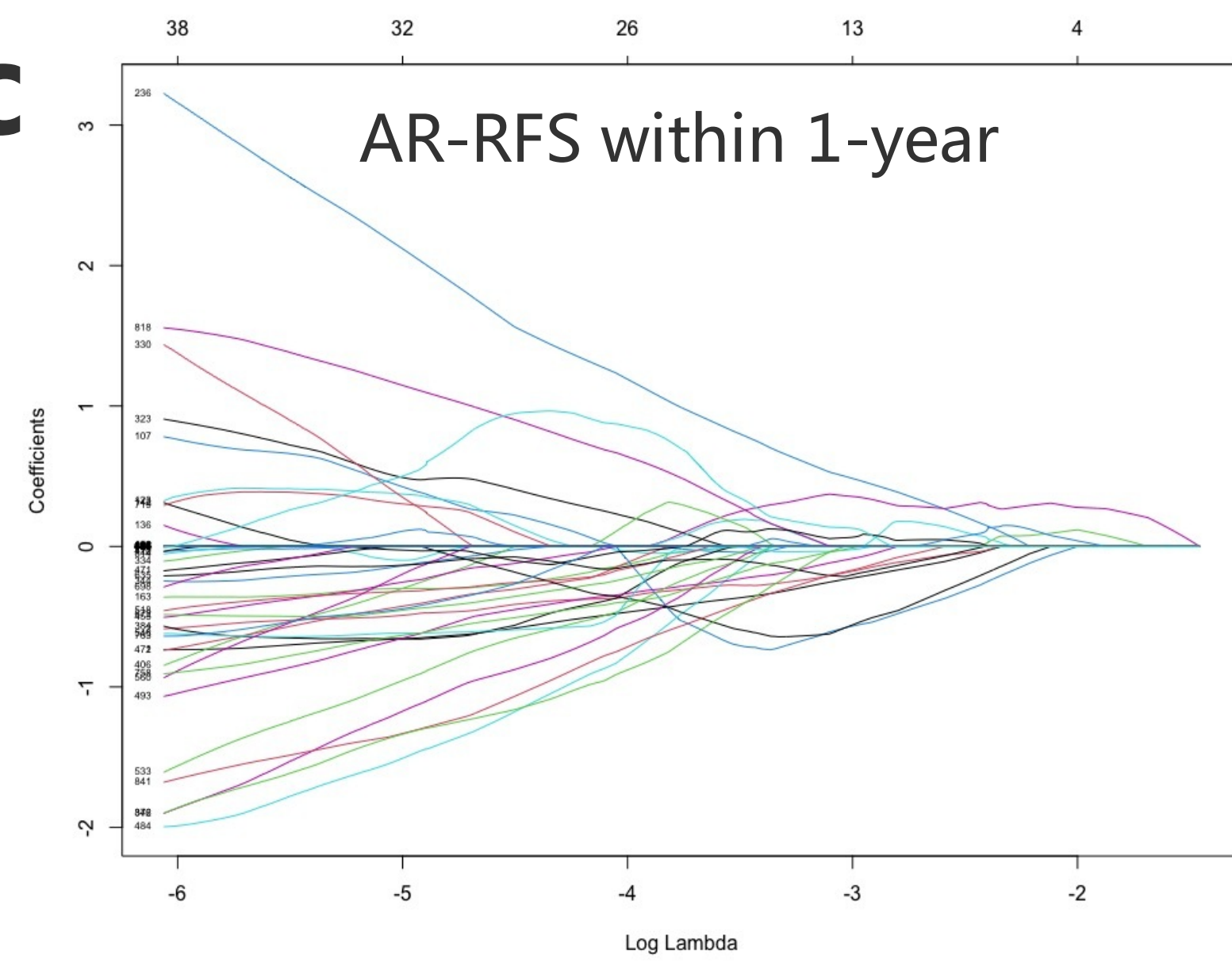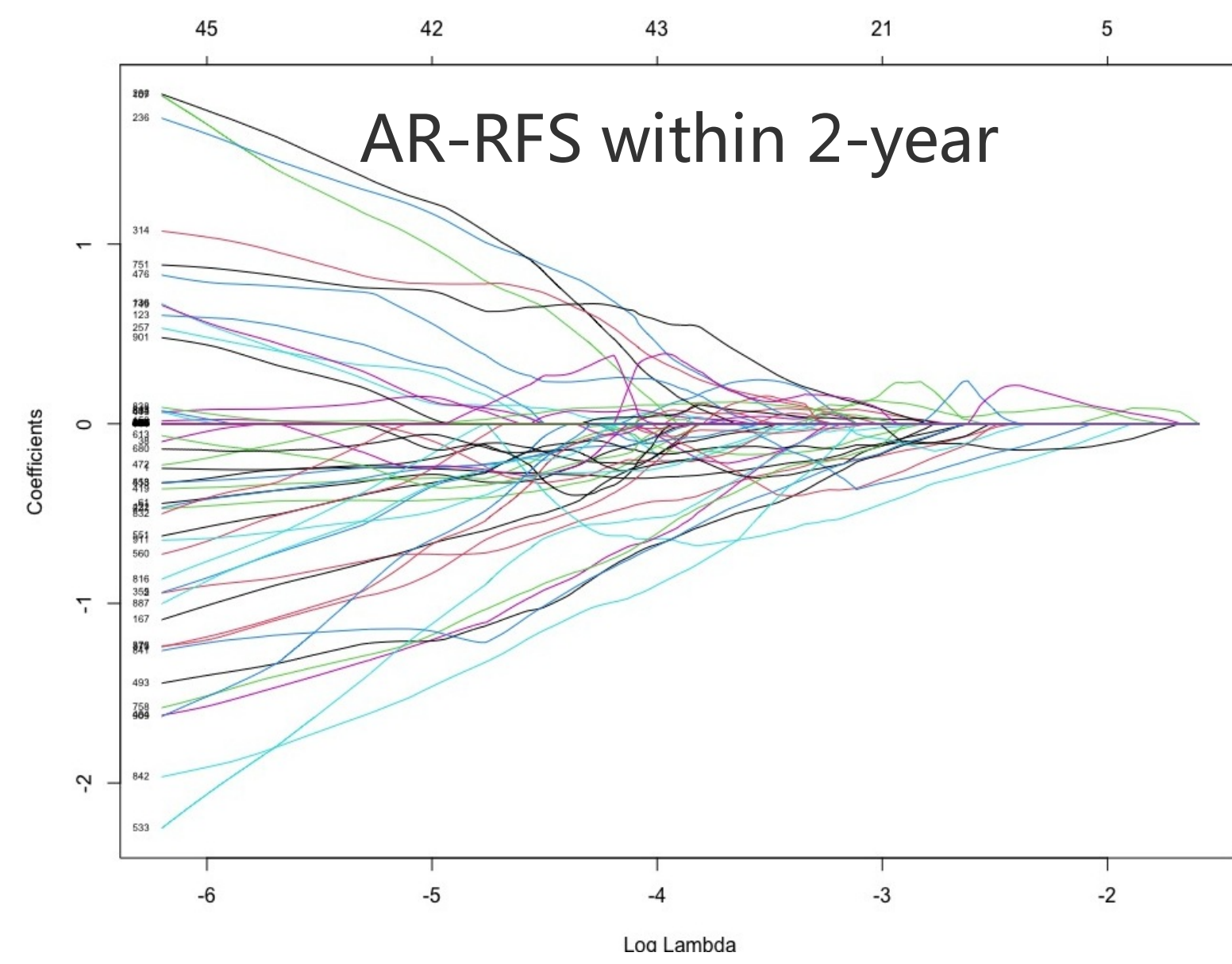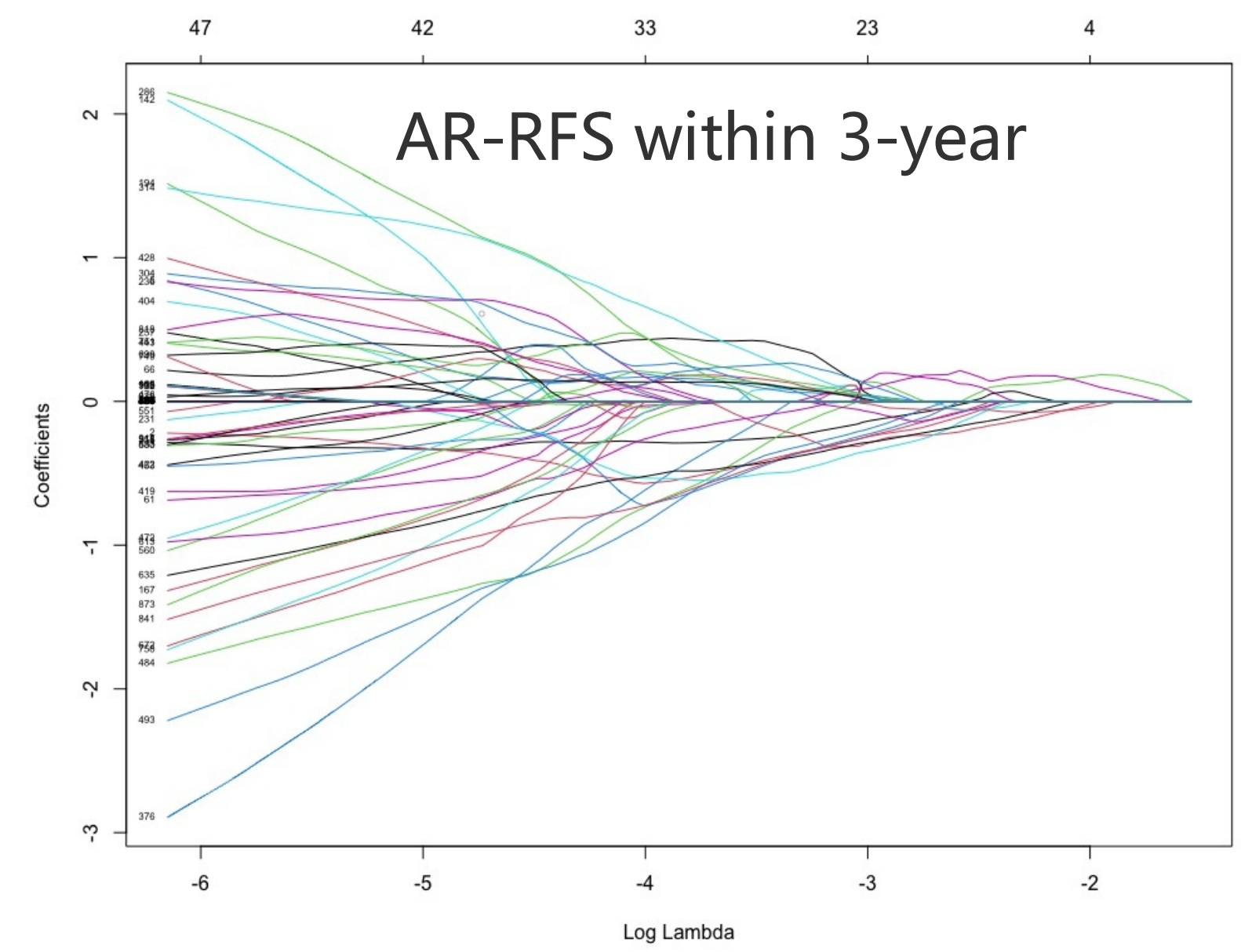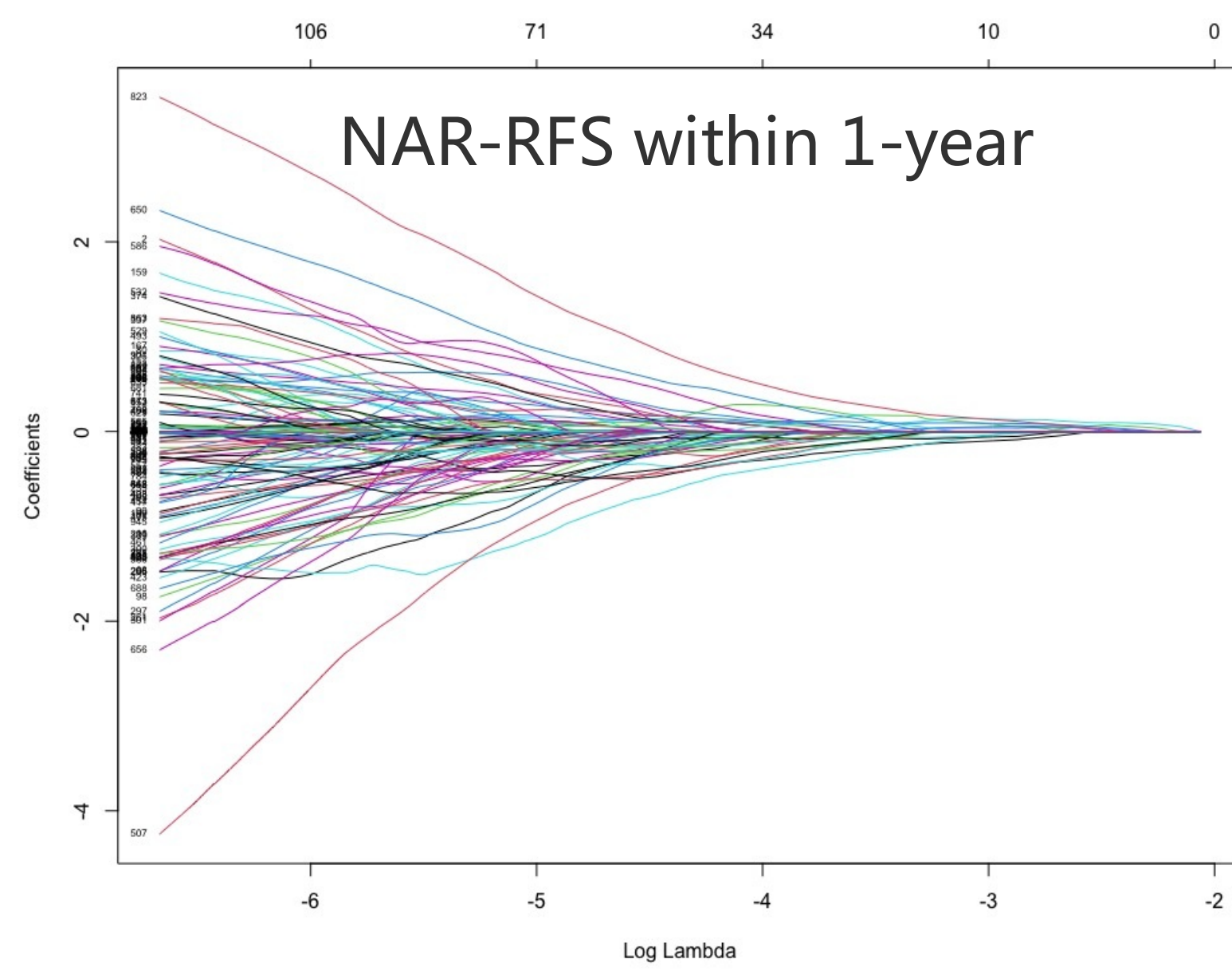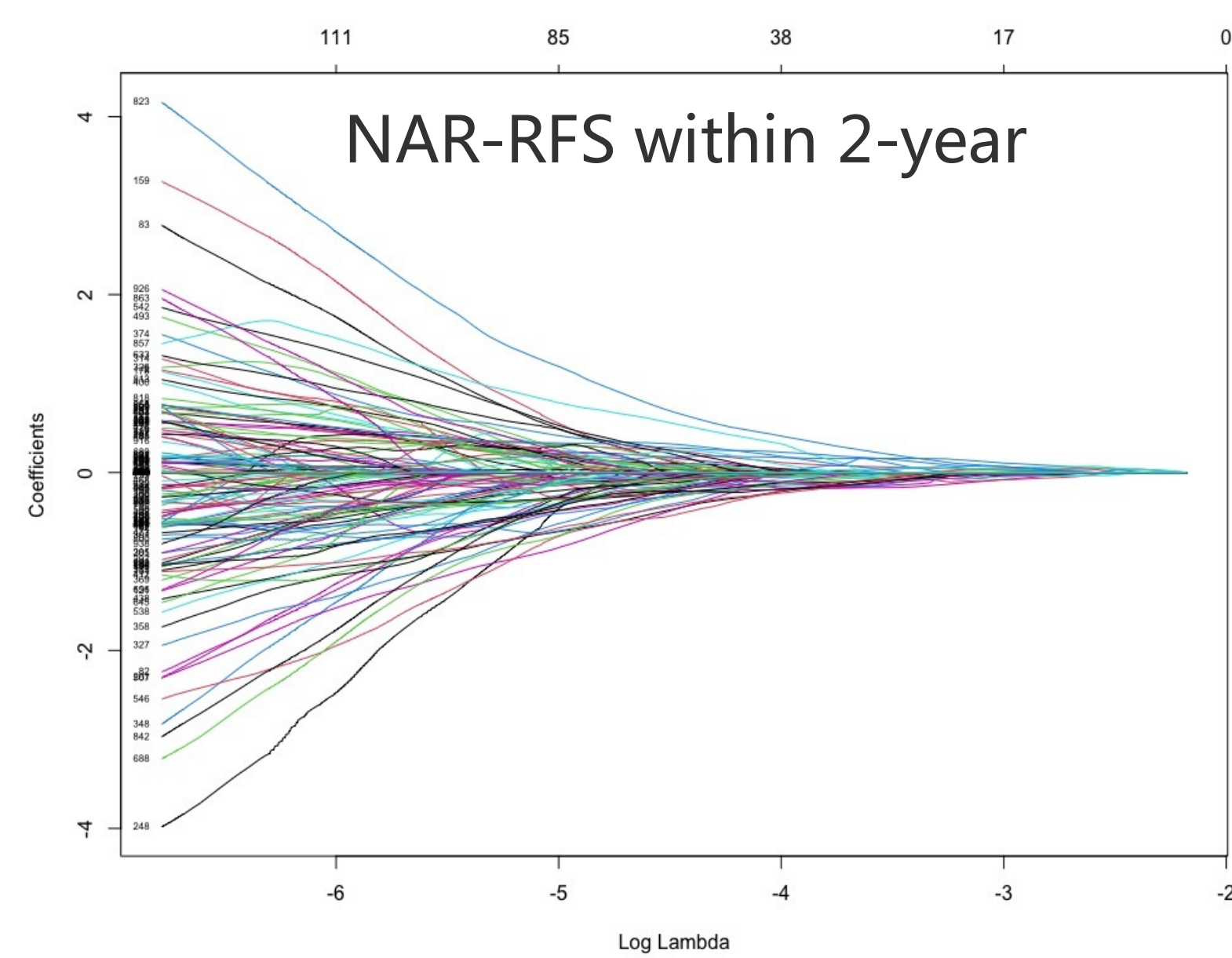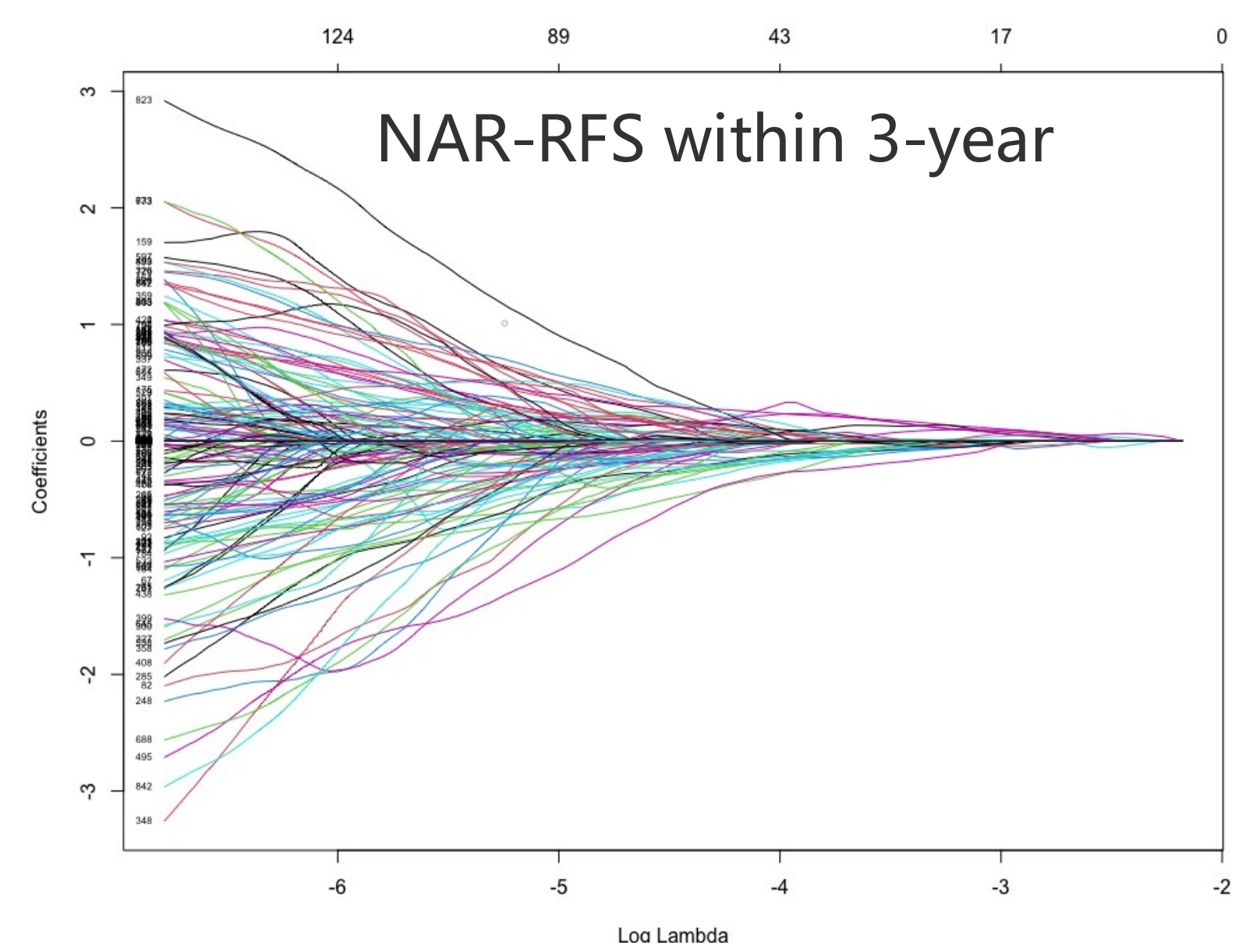

Supplement: Supplementary file 1 [file cancers-15-01784-s001.zip › Figure S3.pdf]

**a**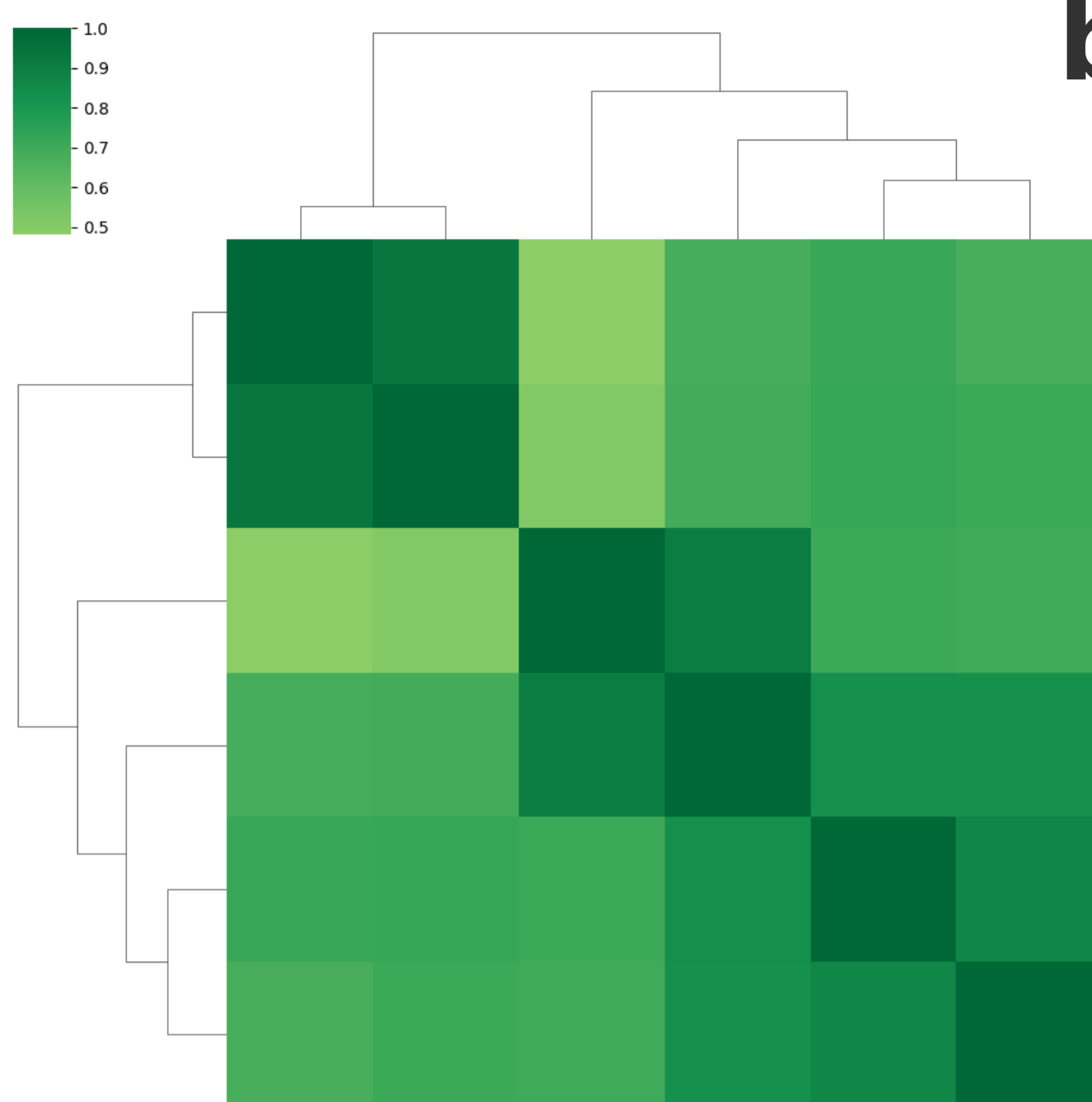**b**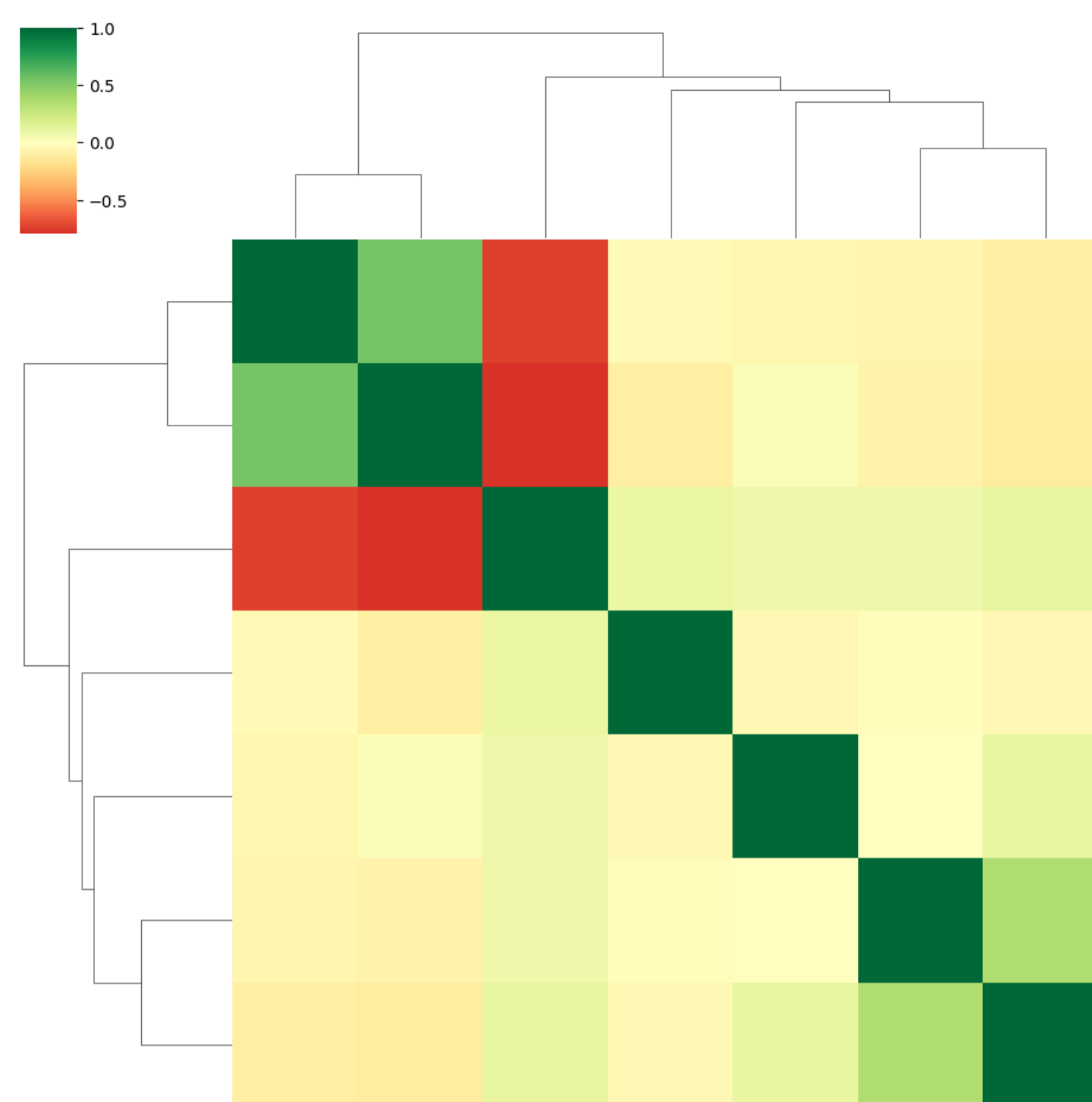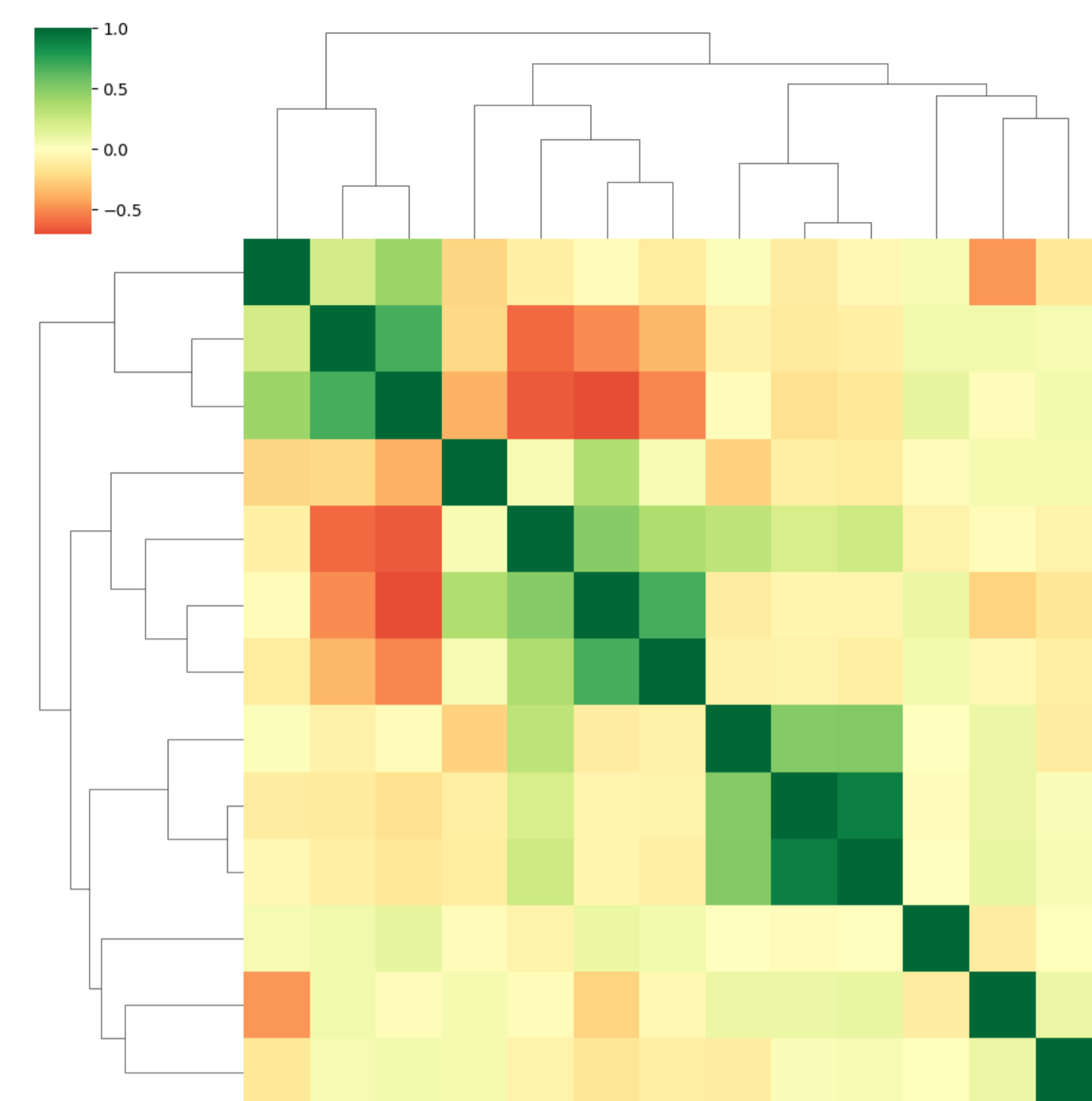**c**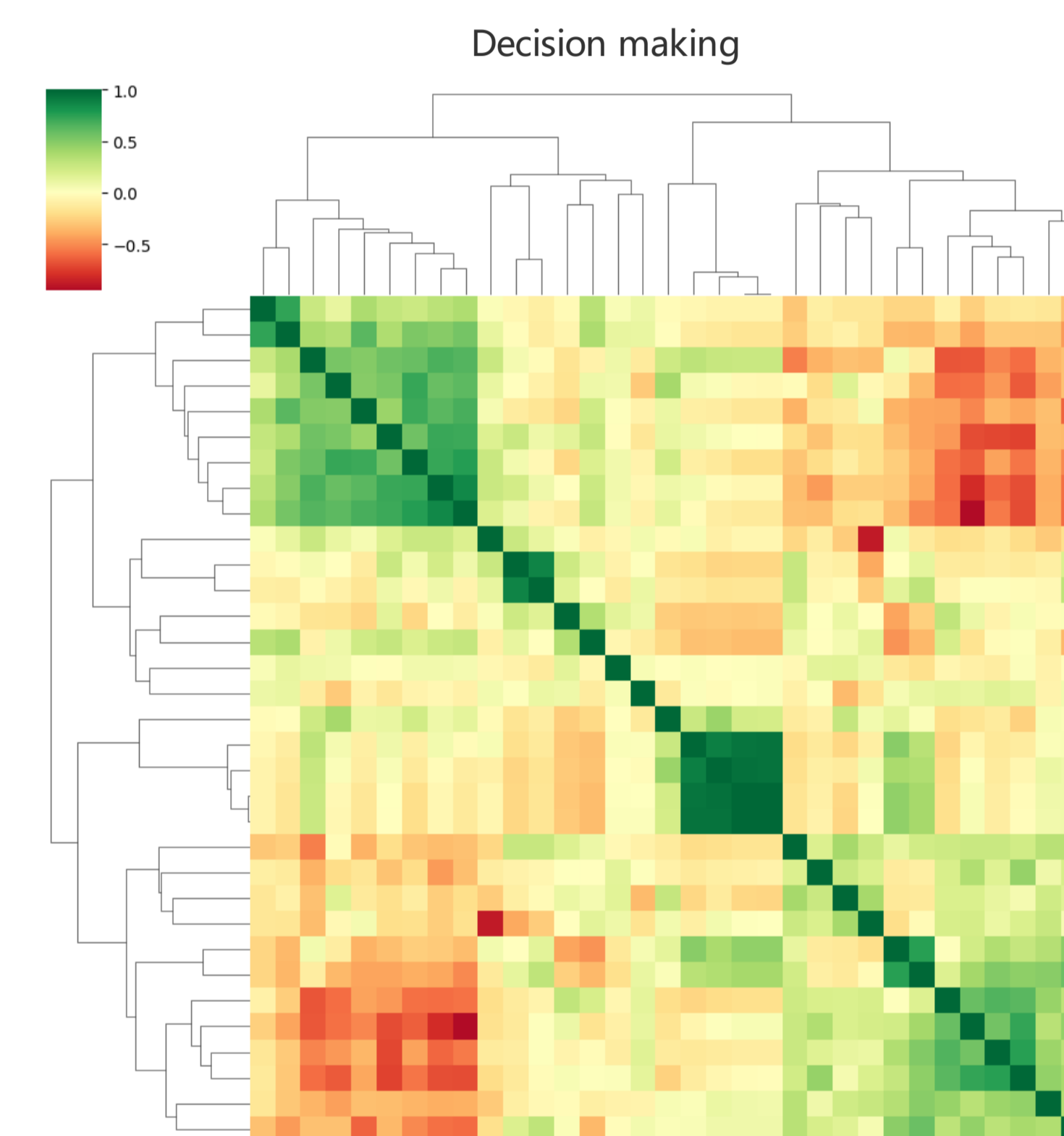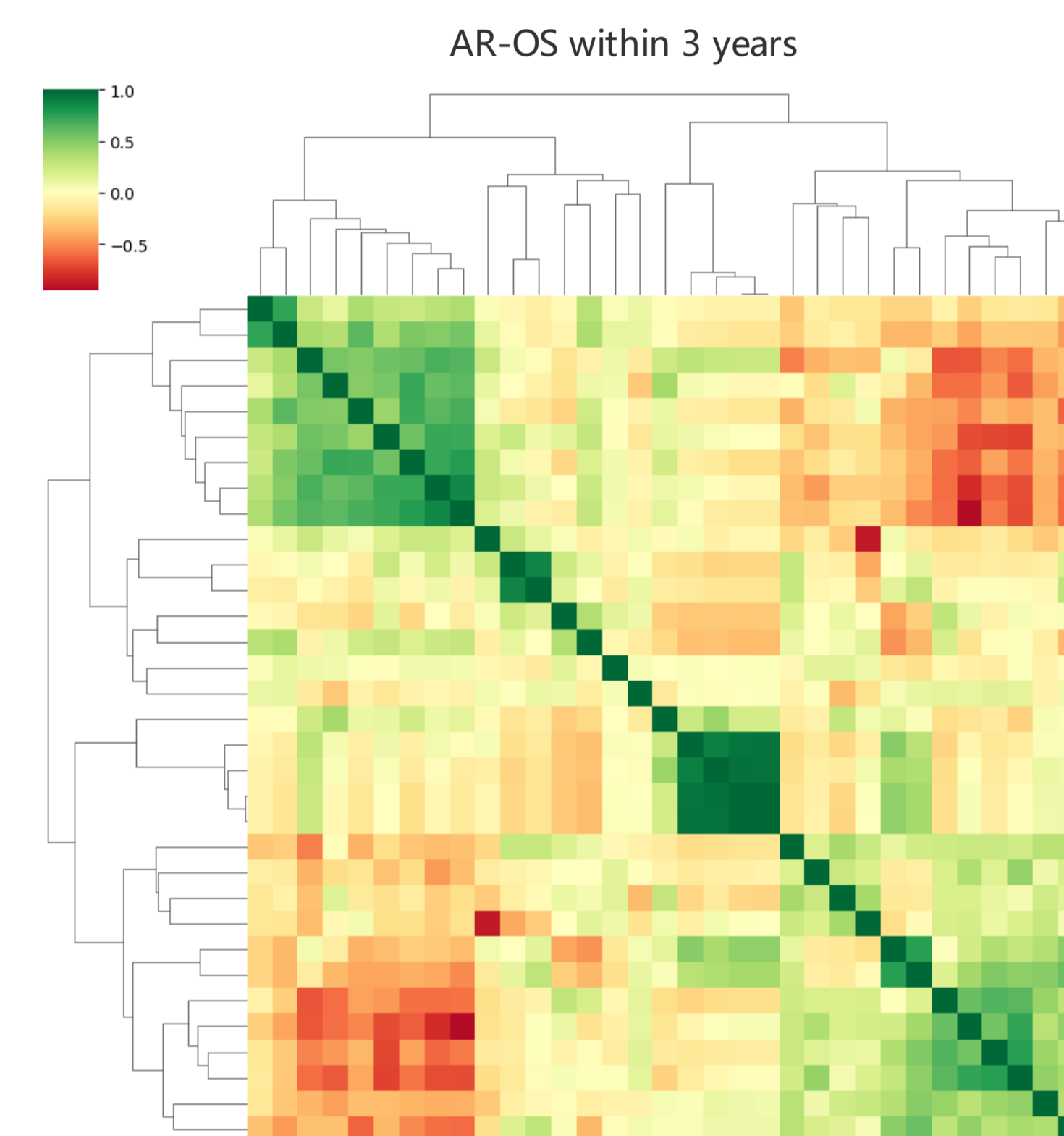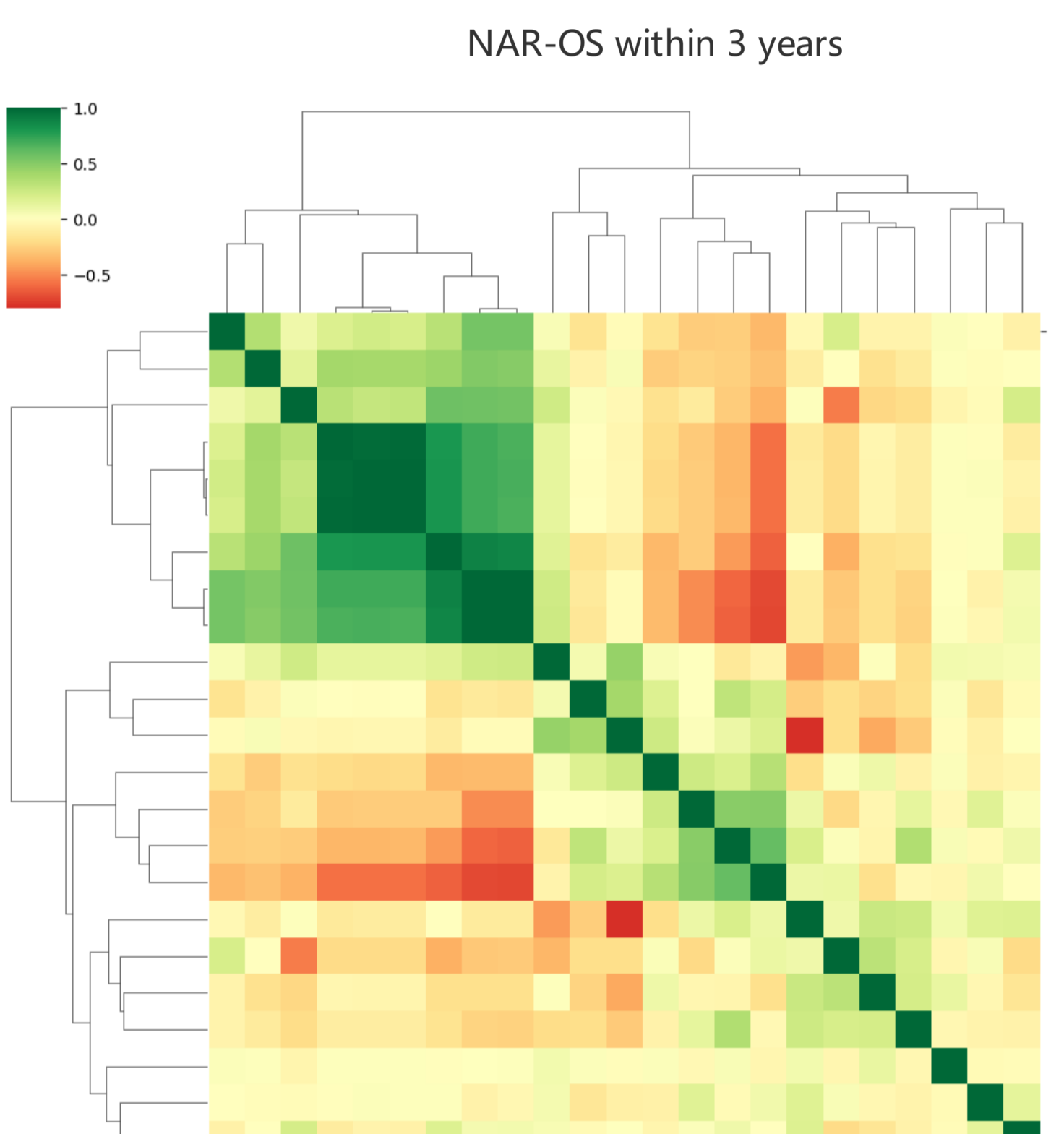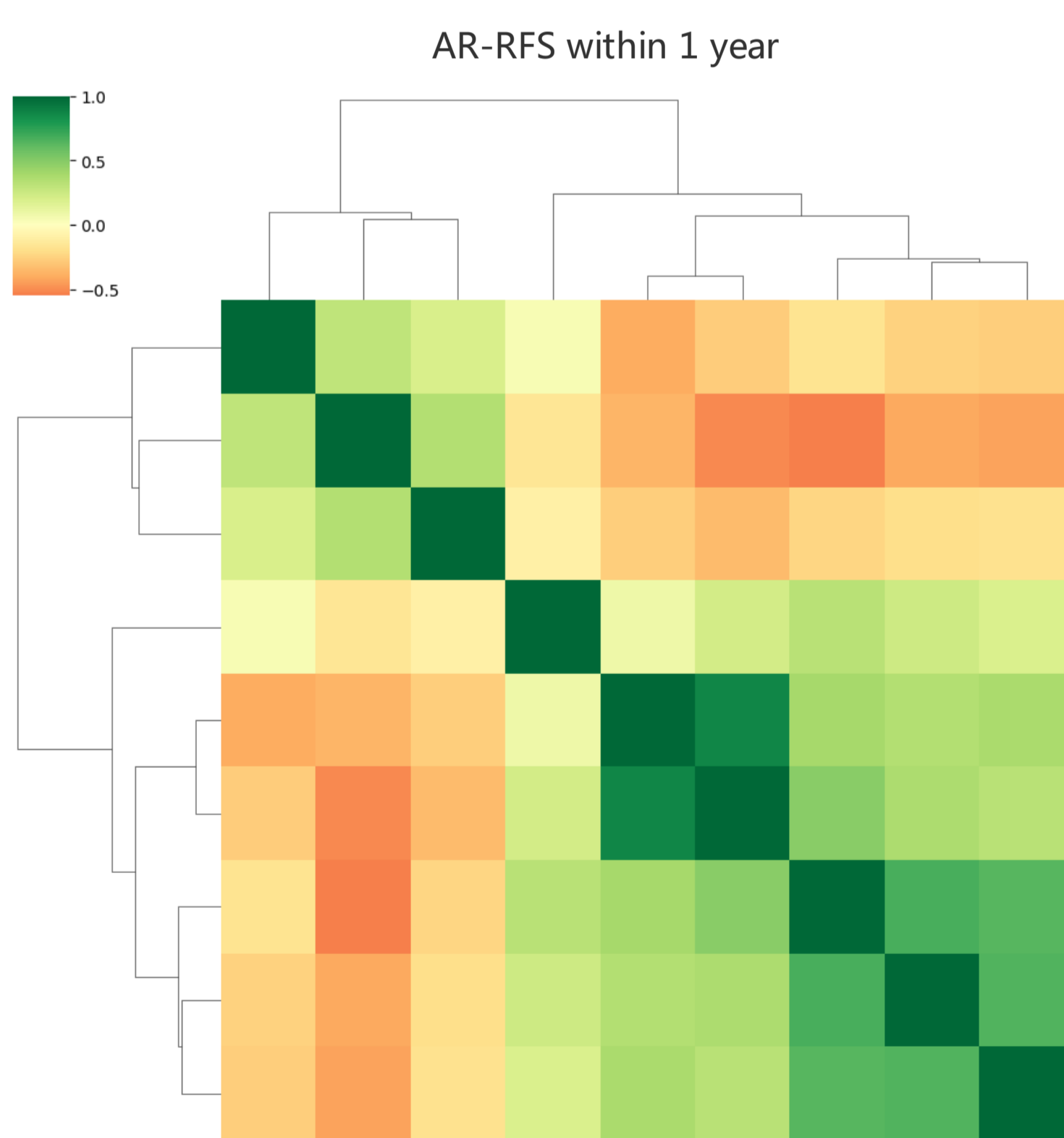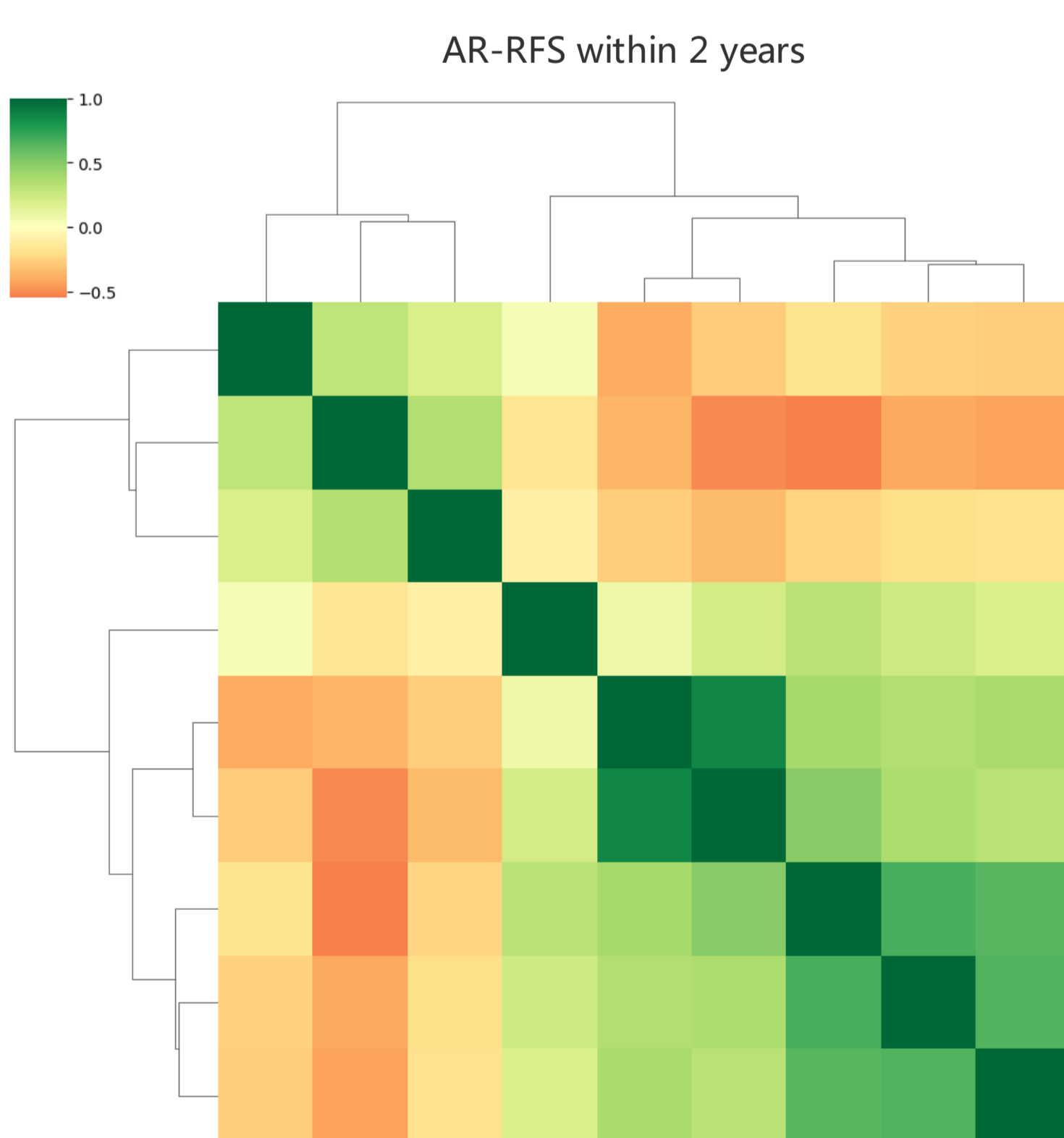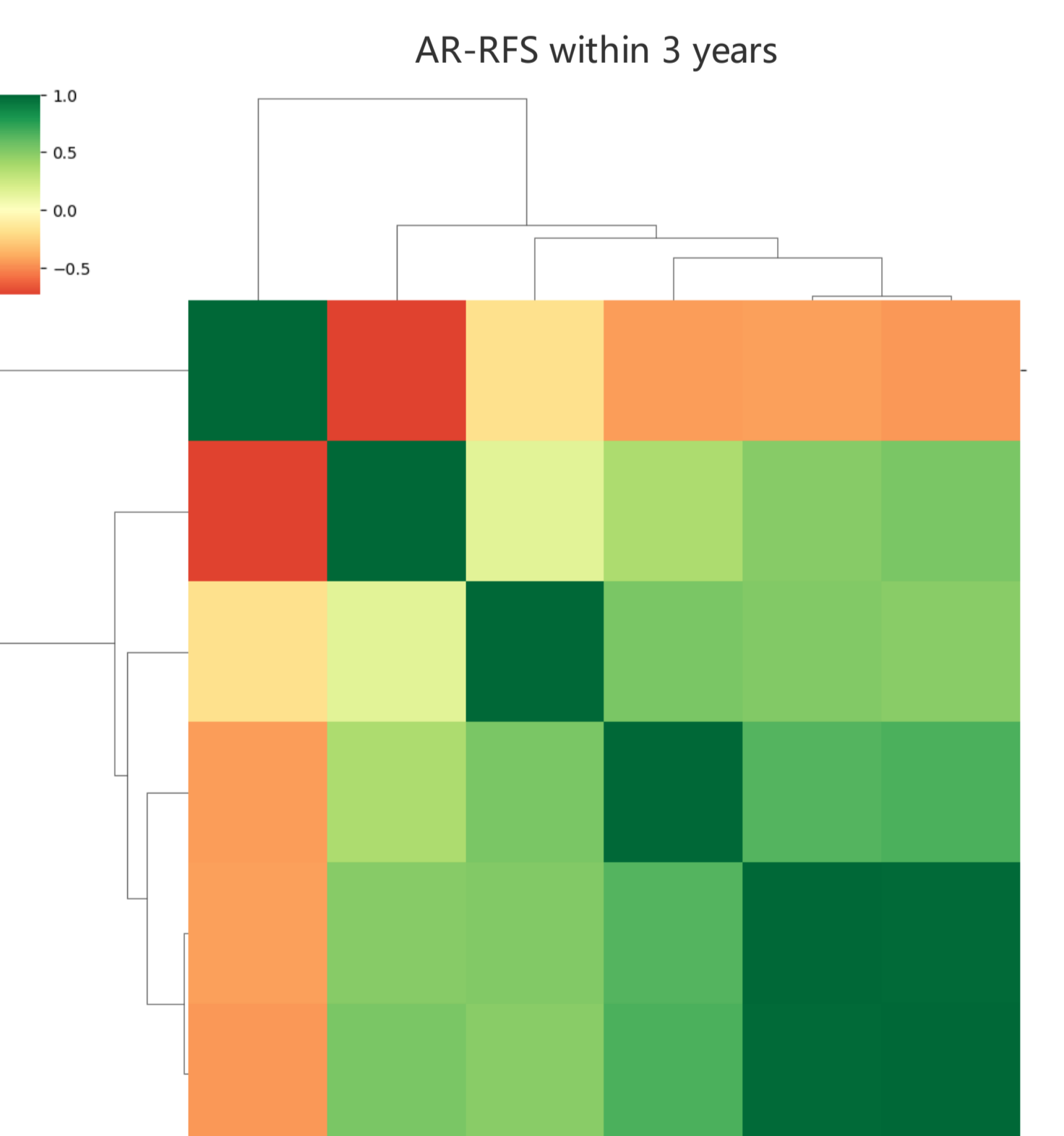

NAR-RFS within 1 year

NAR-RFS within 2 years

NAR-RFS within 3 years

Supplement: Supplementary file 1 [file cancers-15-01784-s001.zip › Figure S4.pdf]

**a**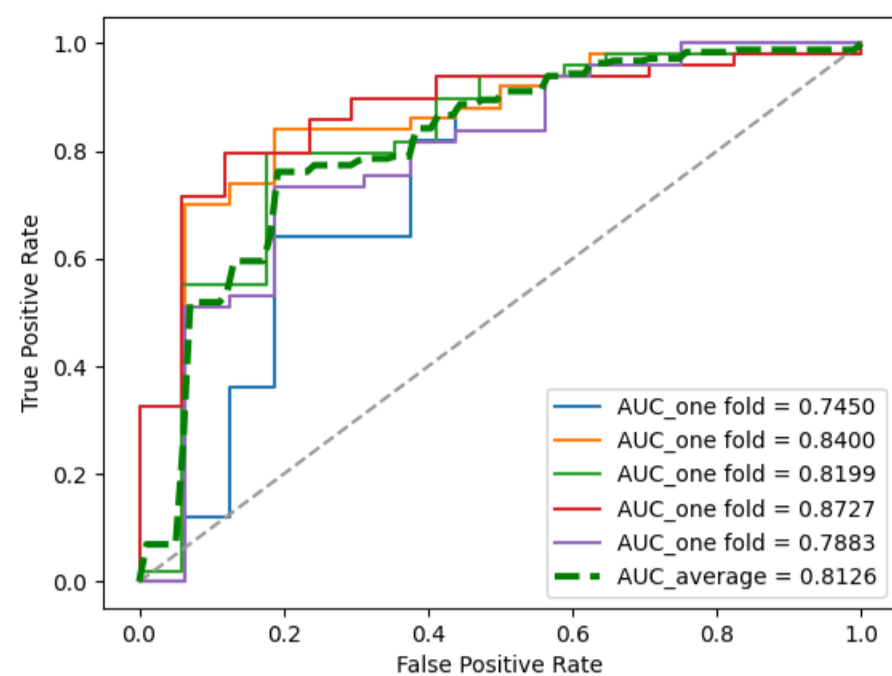**b**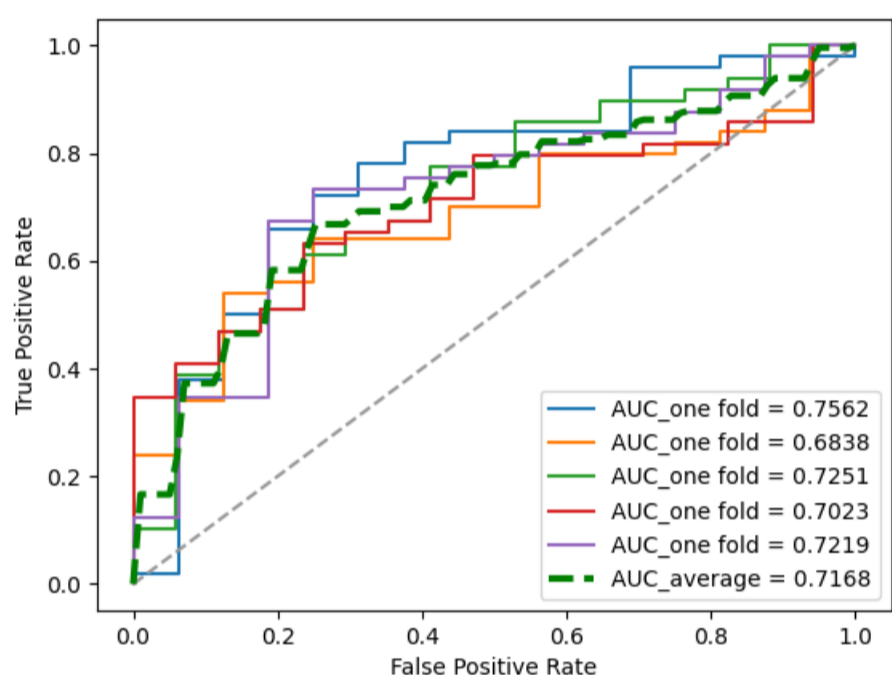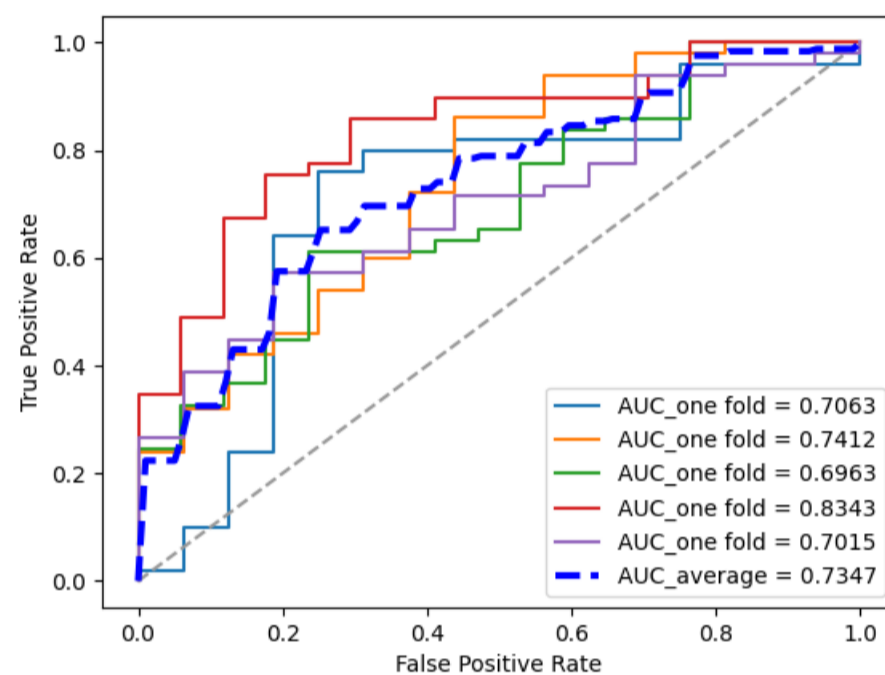**c**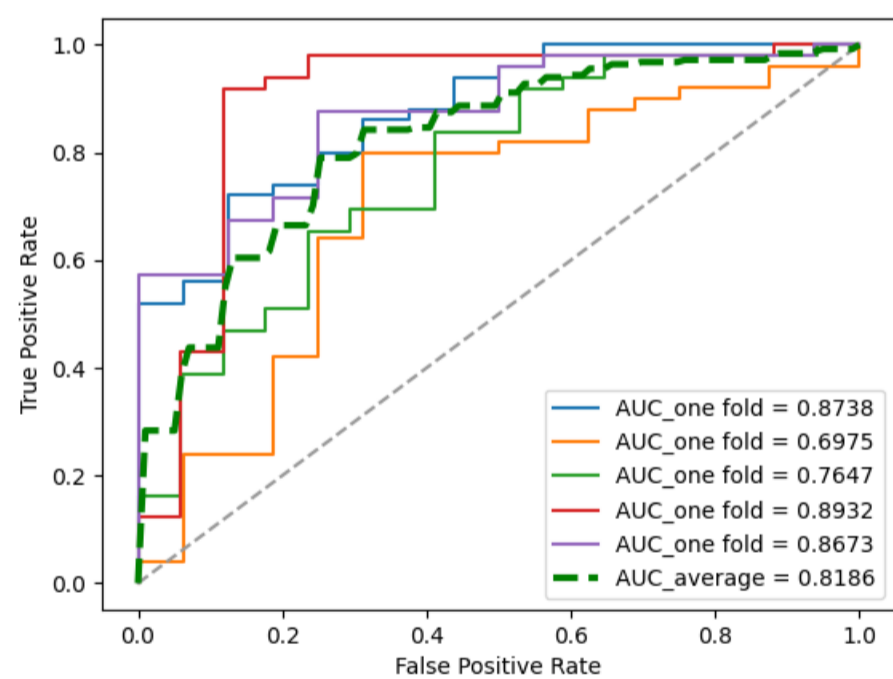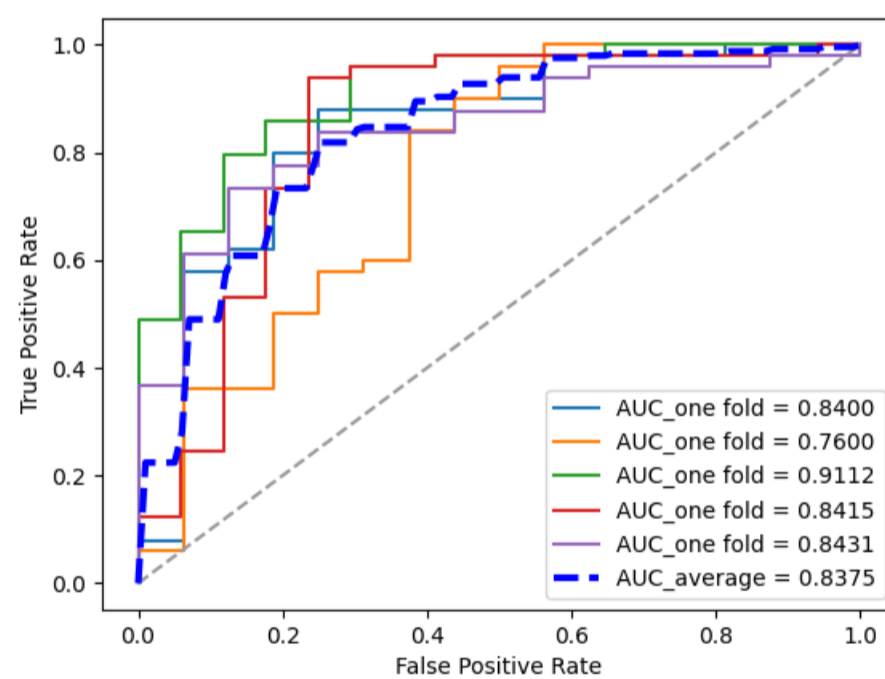**d**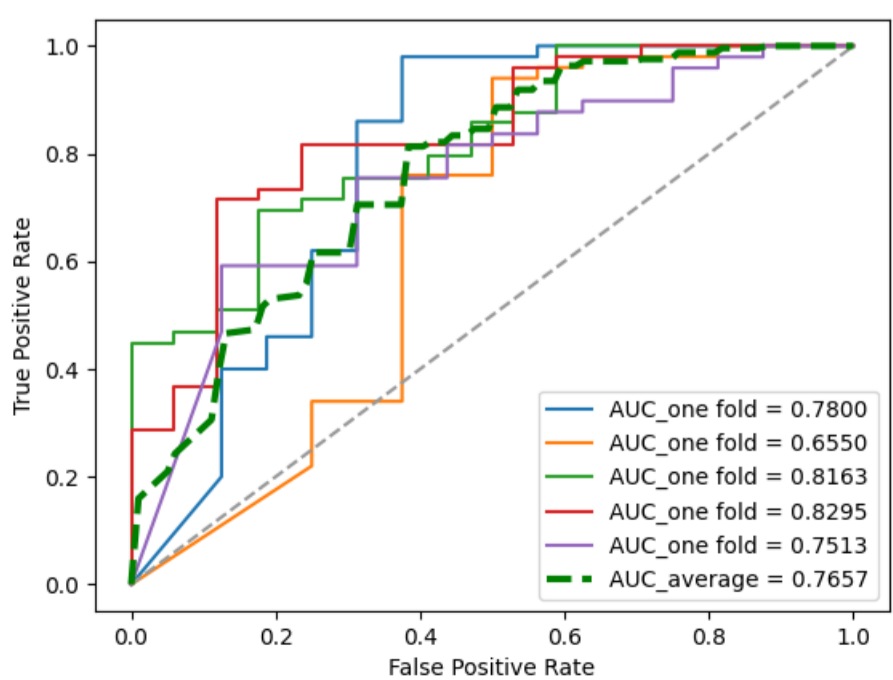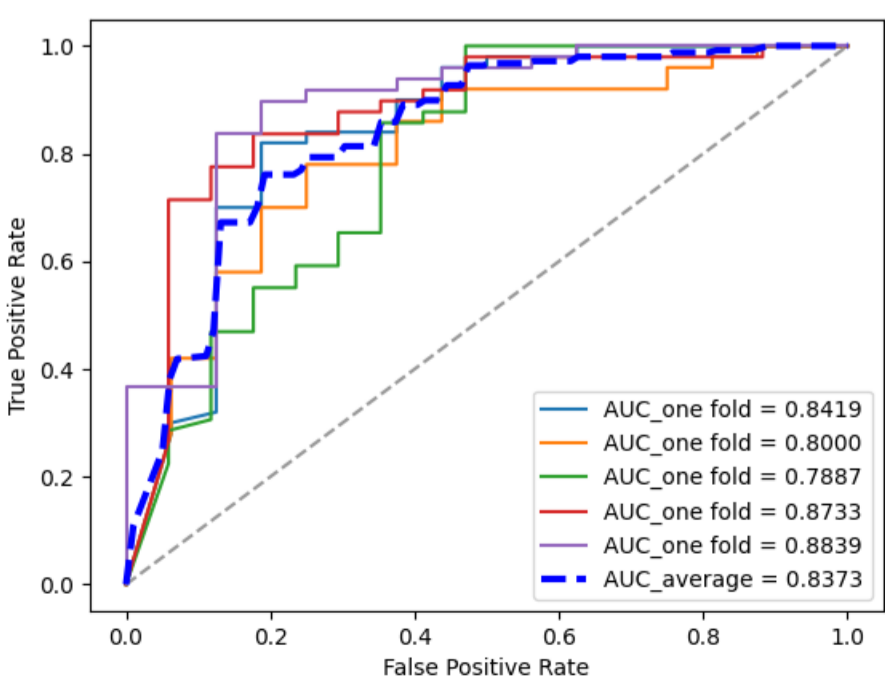

Supplement: Supplementary file 1 [file cancers-15-01784-s001.zip › Figure S5.pdf]

**a**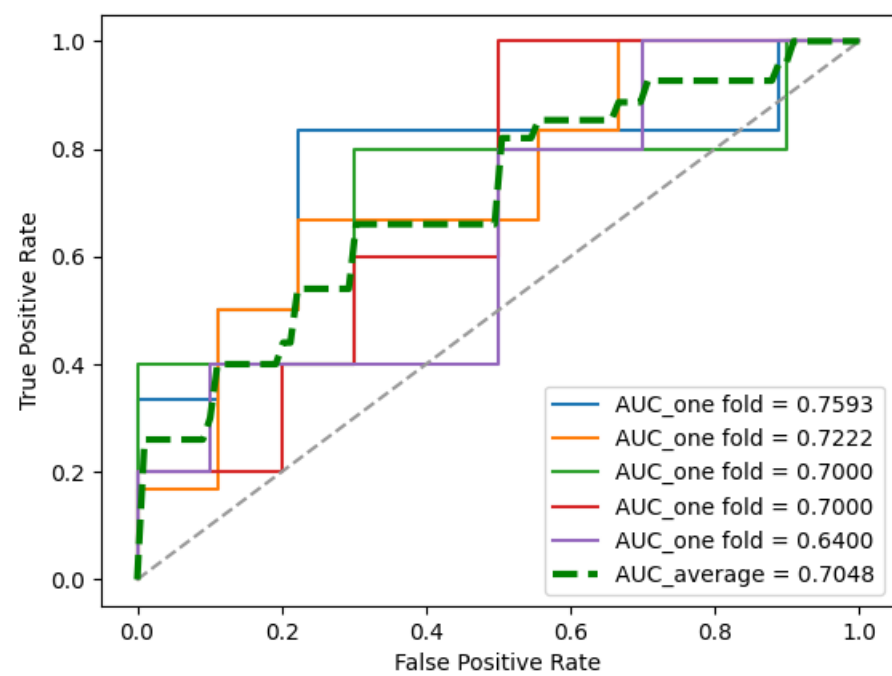**b**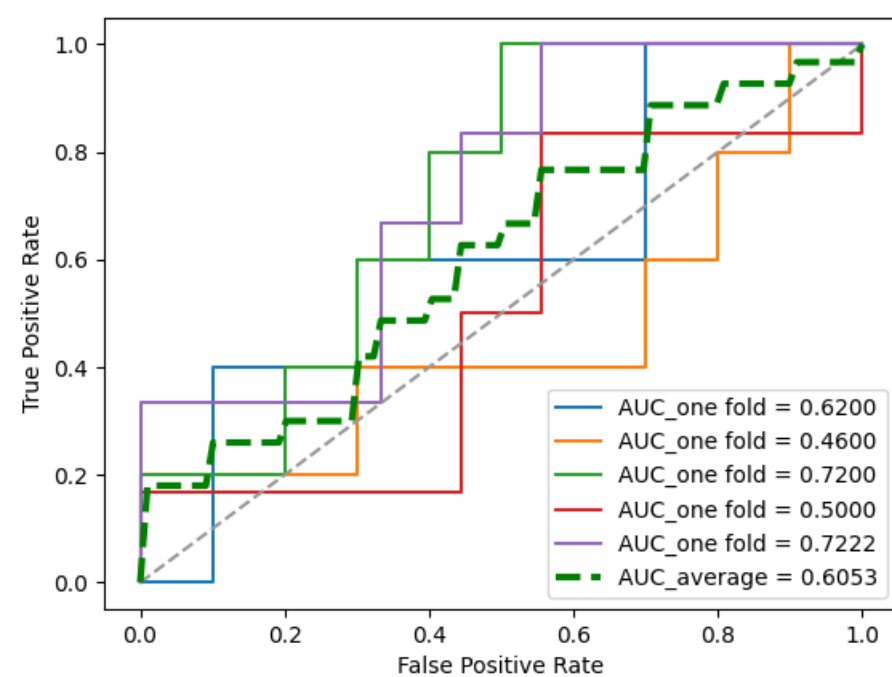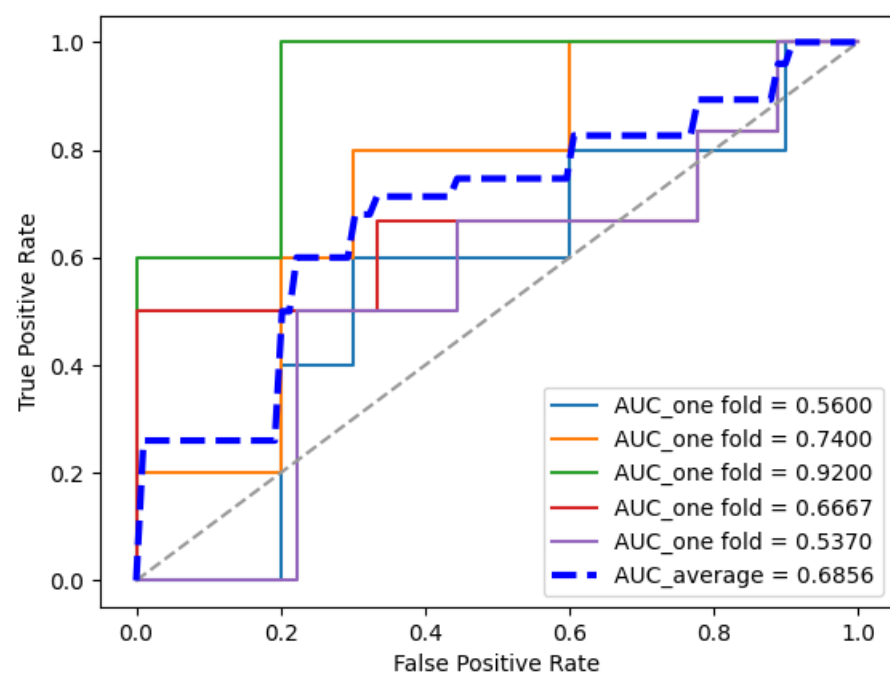**c**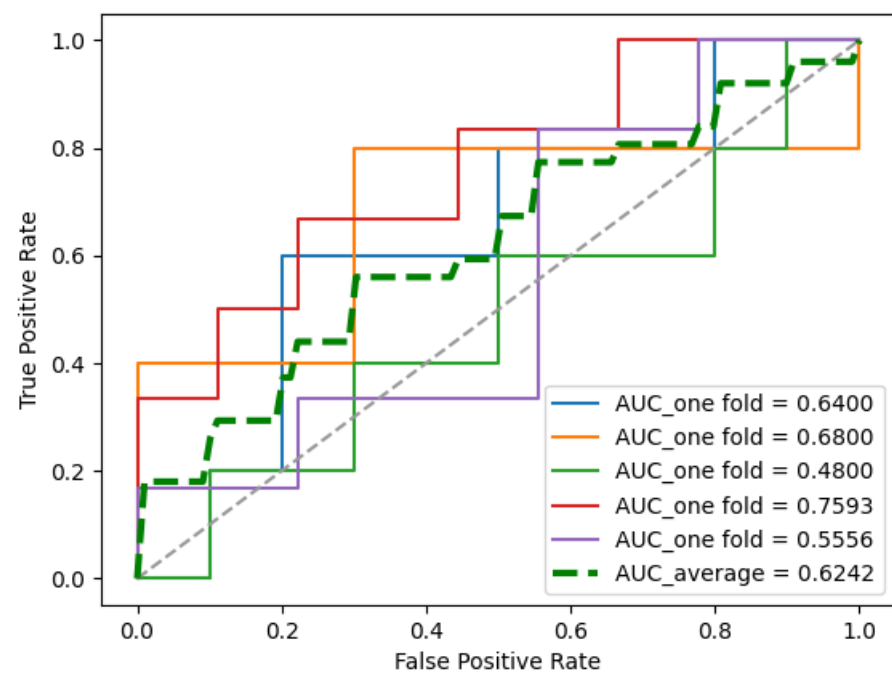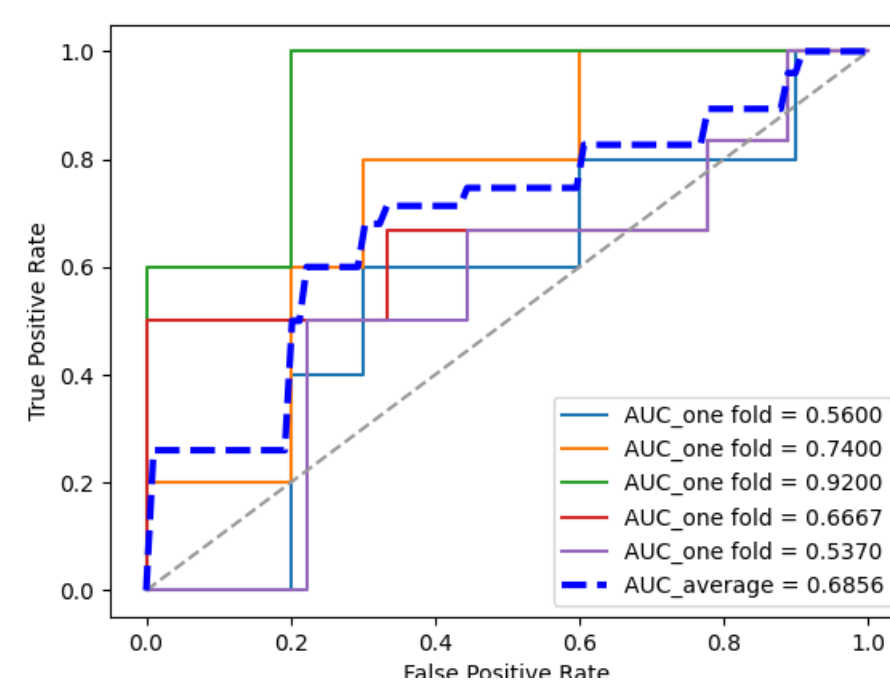**d**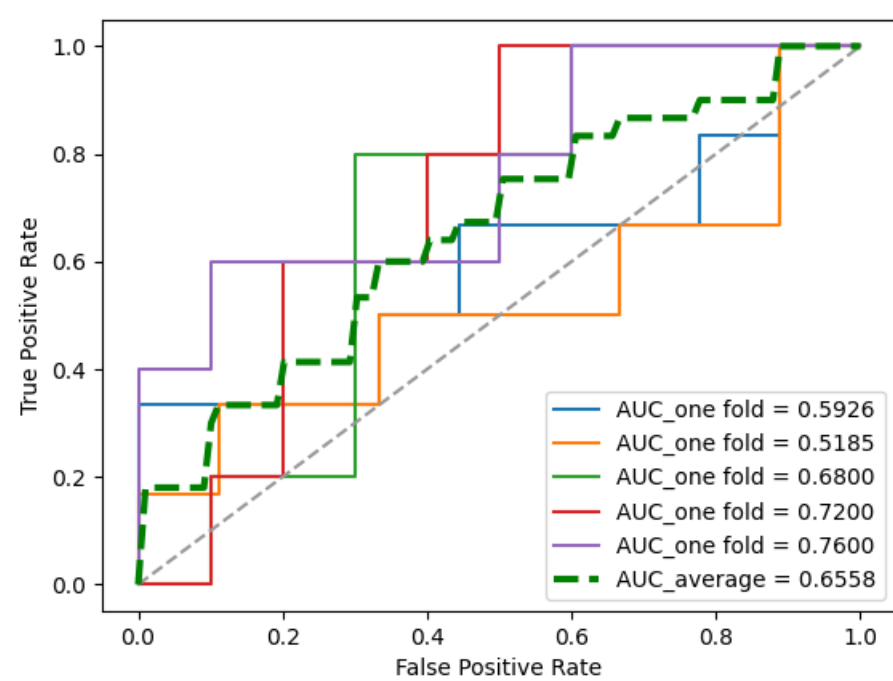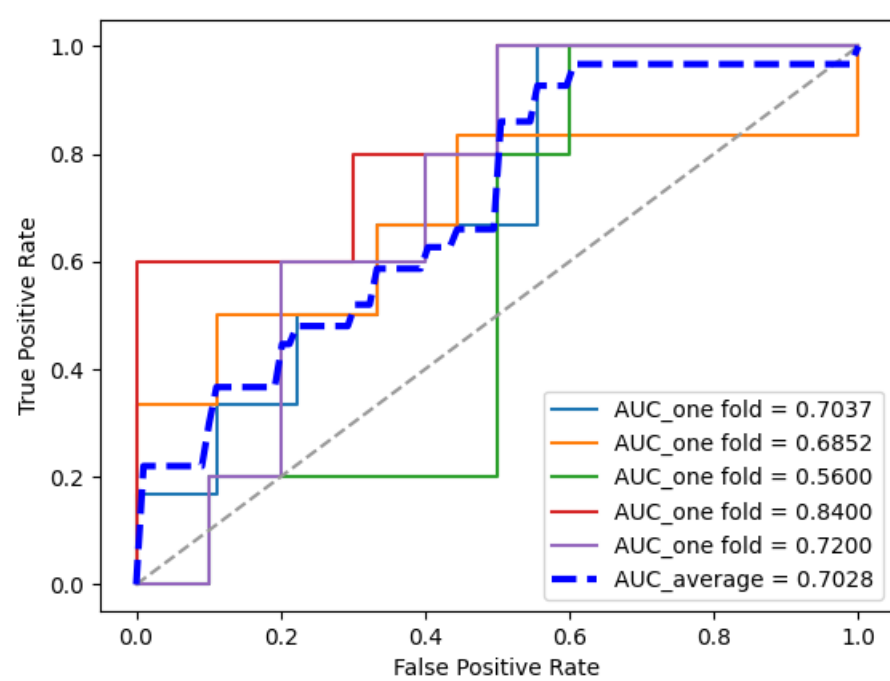

Supplement: Supplementary file 1 [file cancers-15-01784-s001.zip › Figure S6.pdf]

**a**

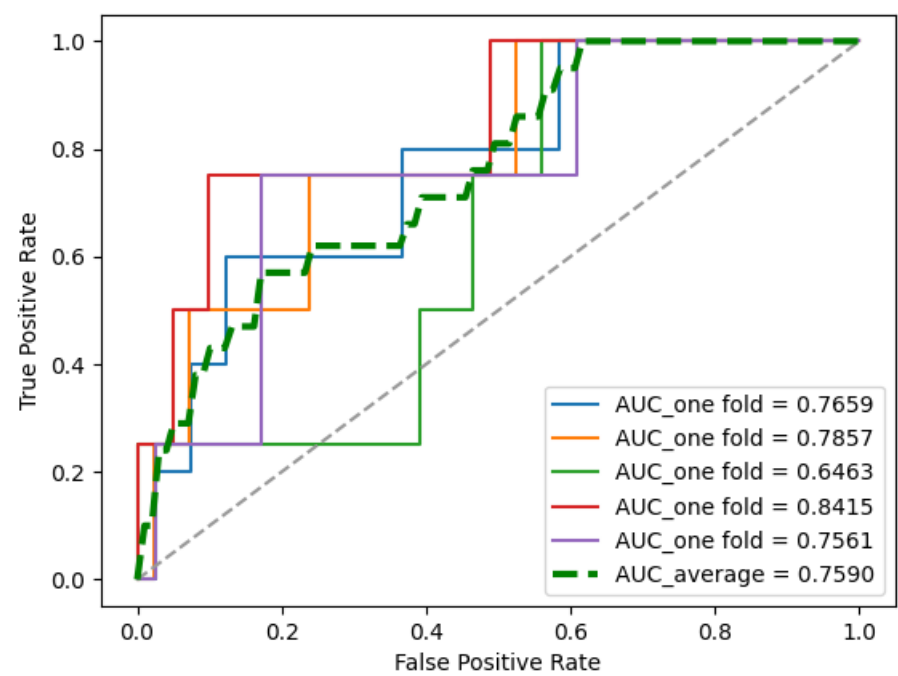

**b**

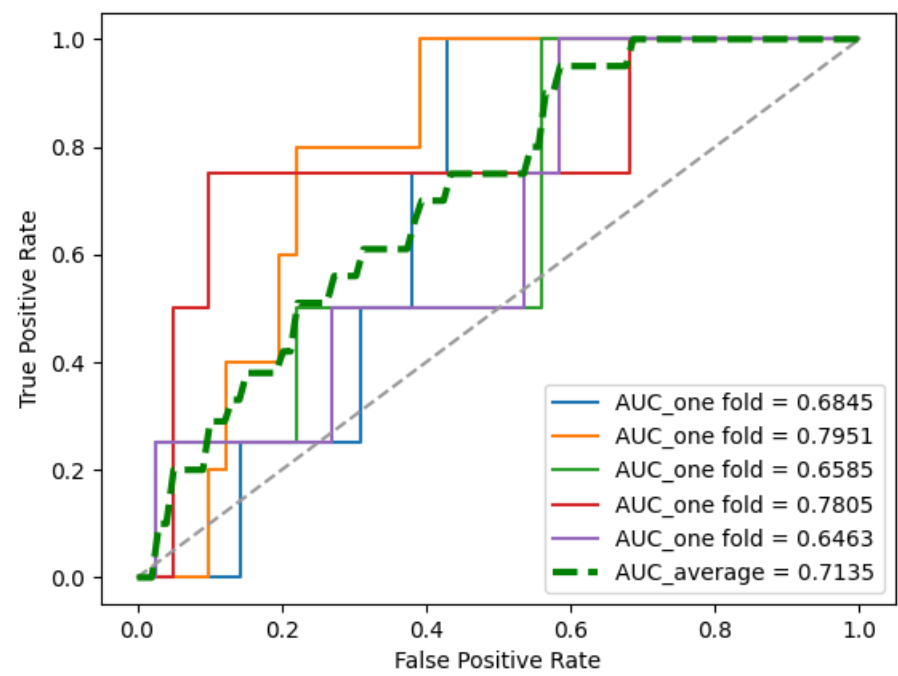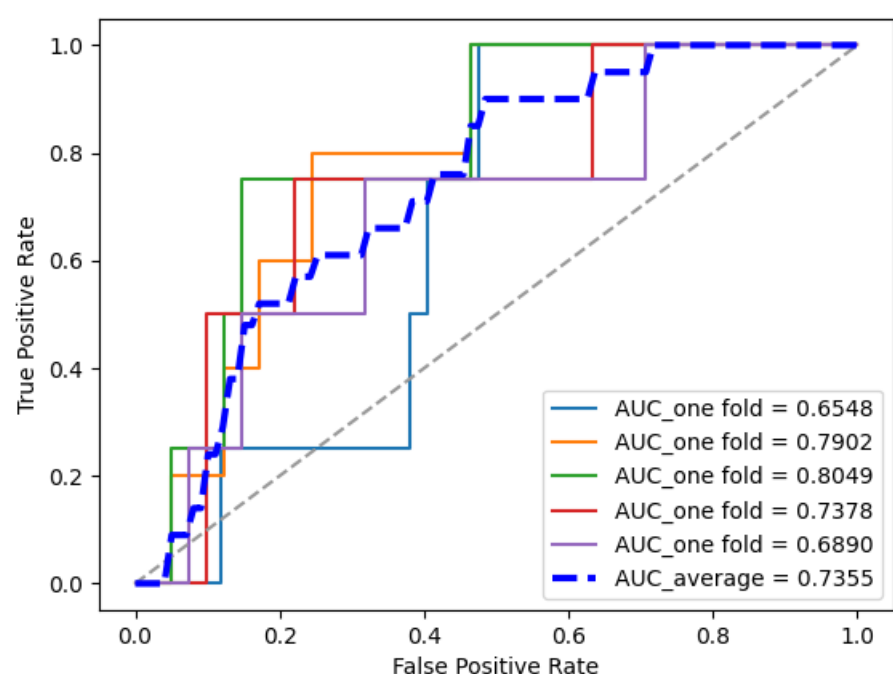

**c**

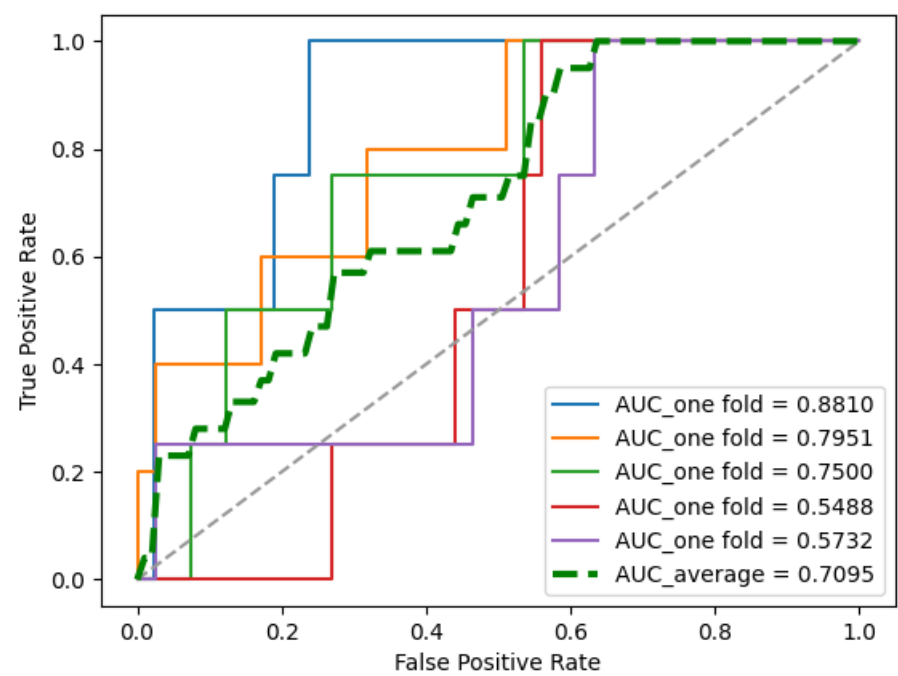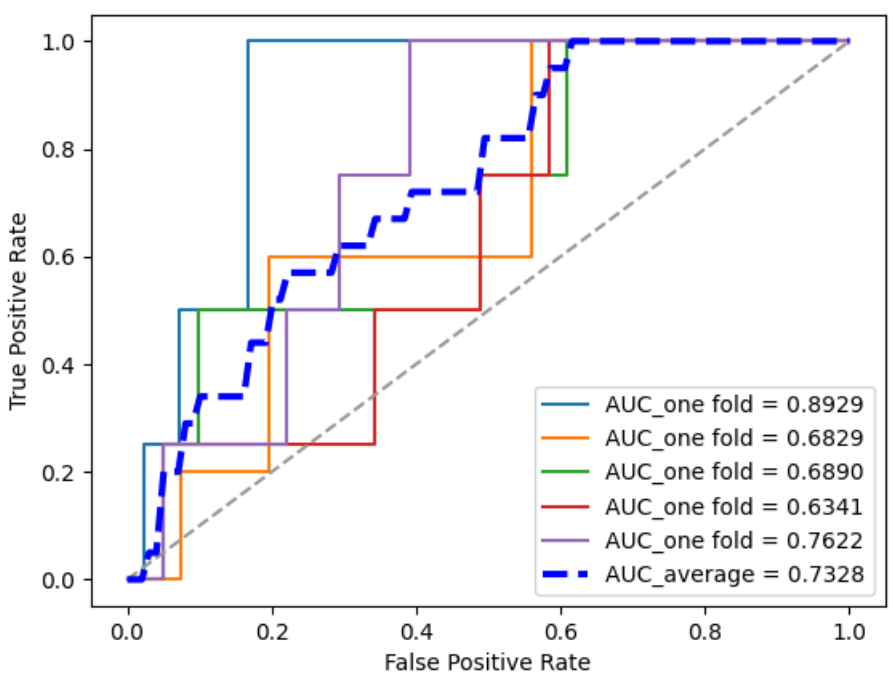

**d**

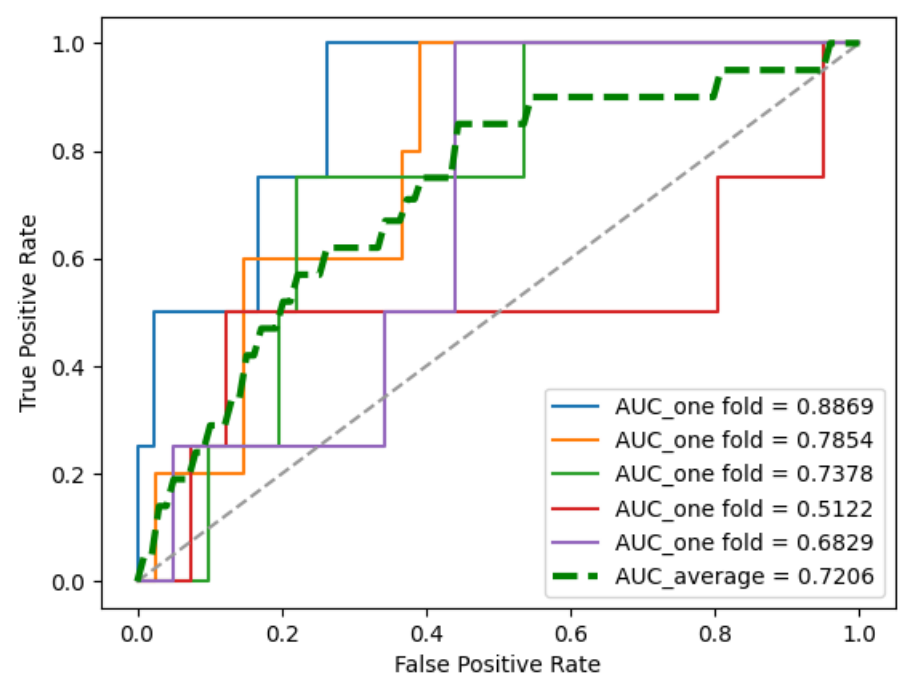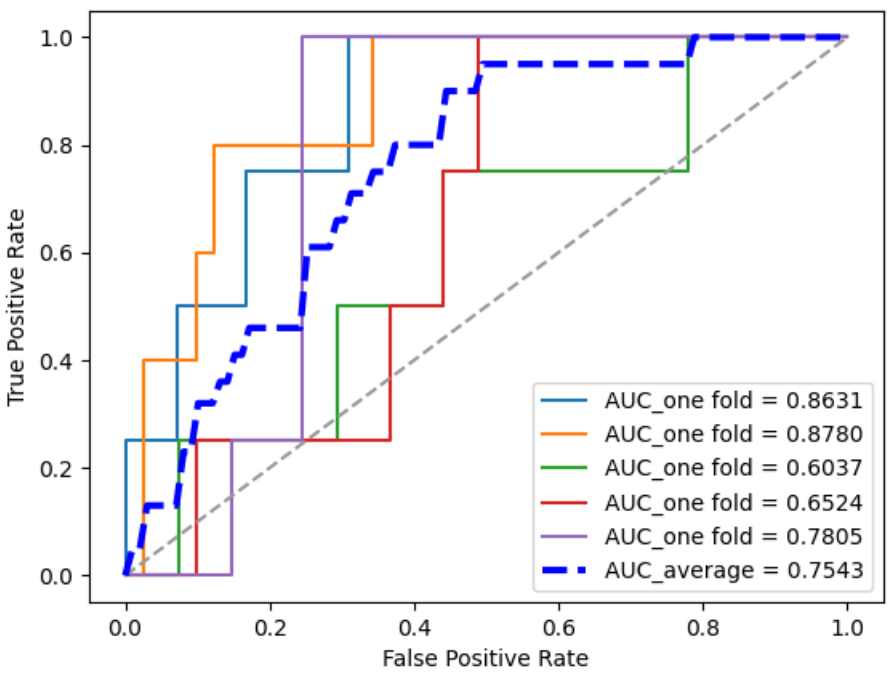

Supplement: Supplementary file 1 [file cancers-15-01784-s001.zip › Figure S7.pdf]

**a**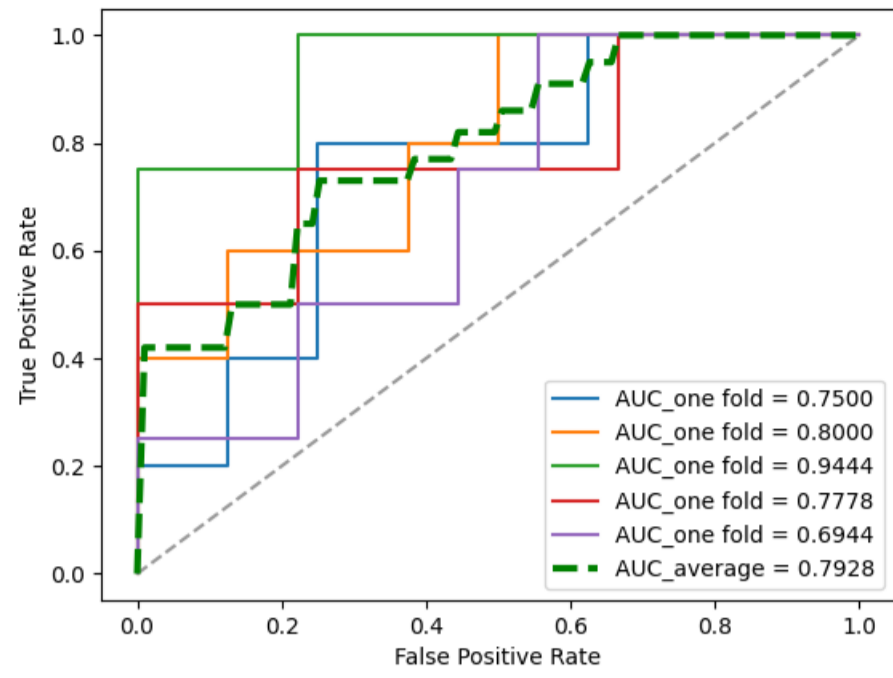**b**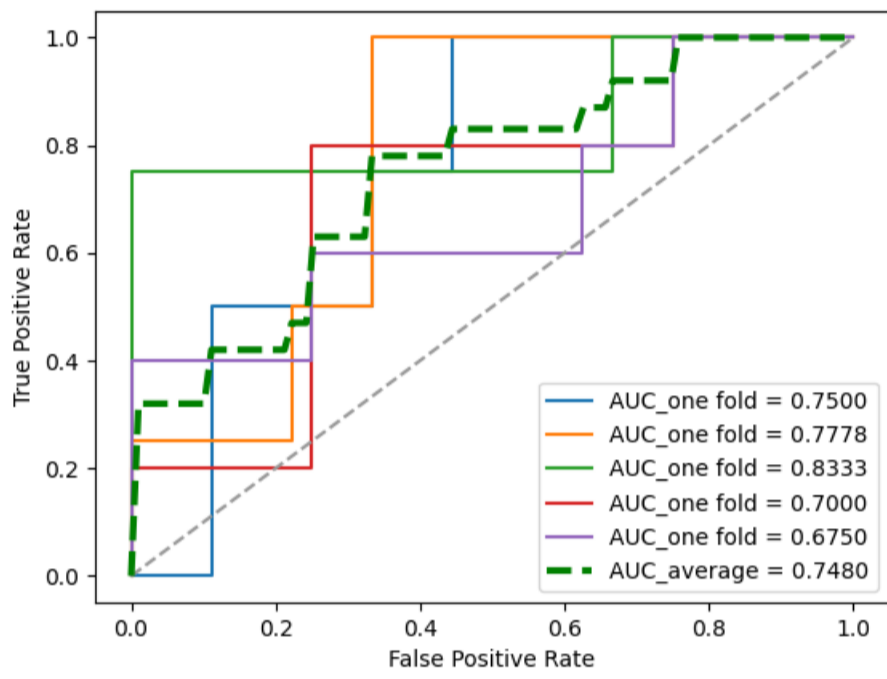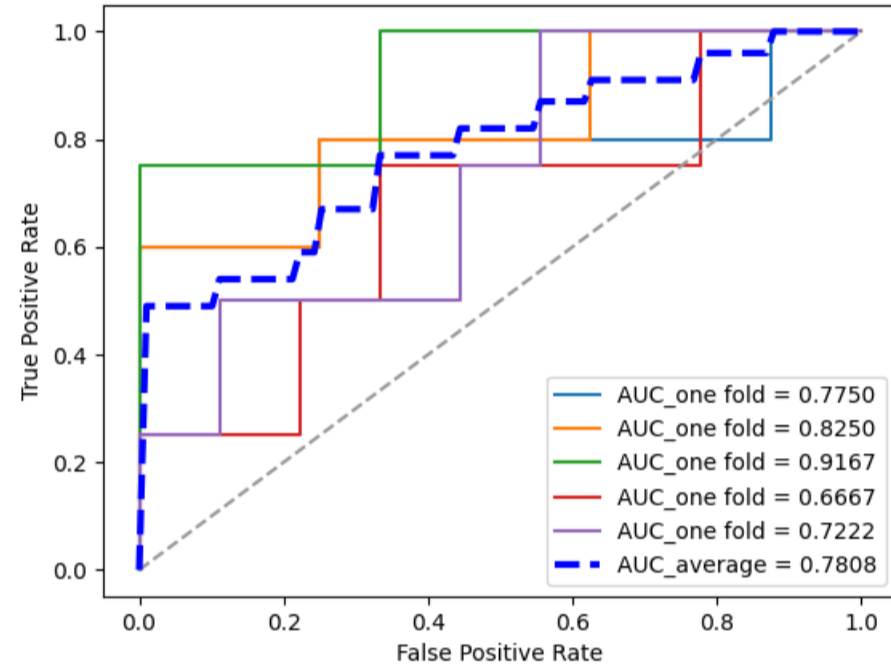**c**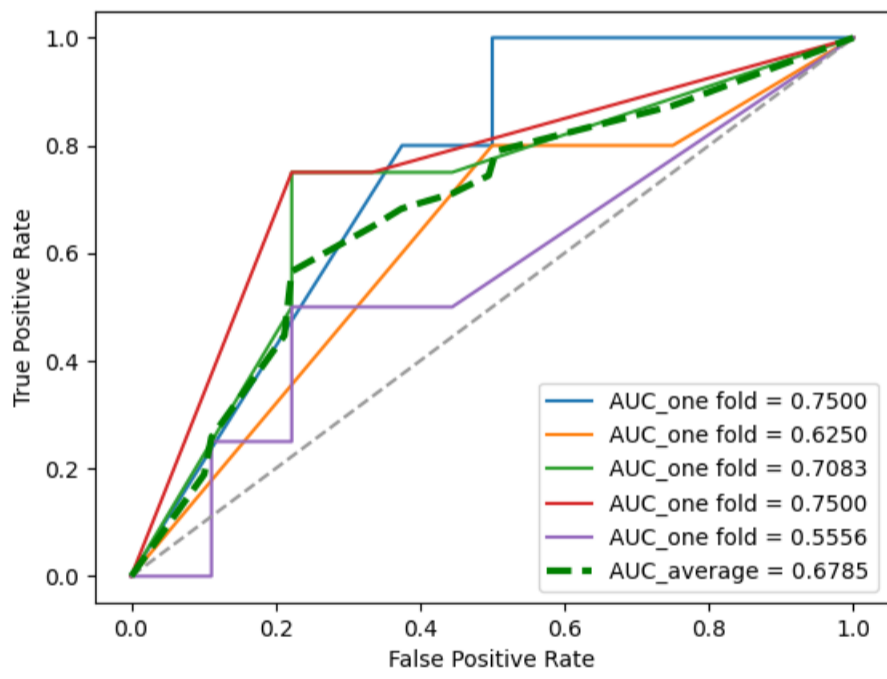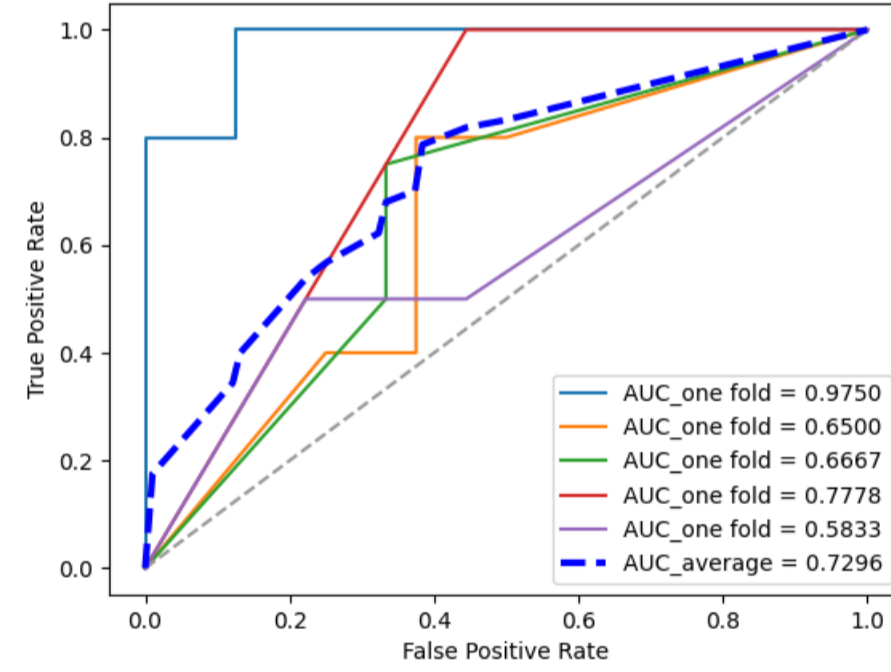**d**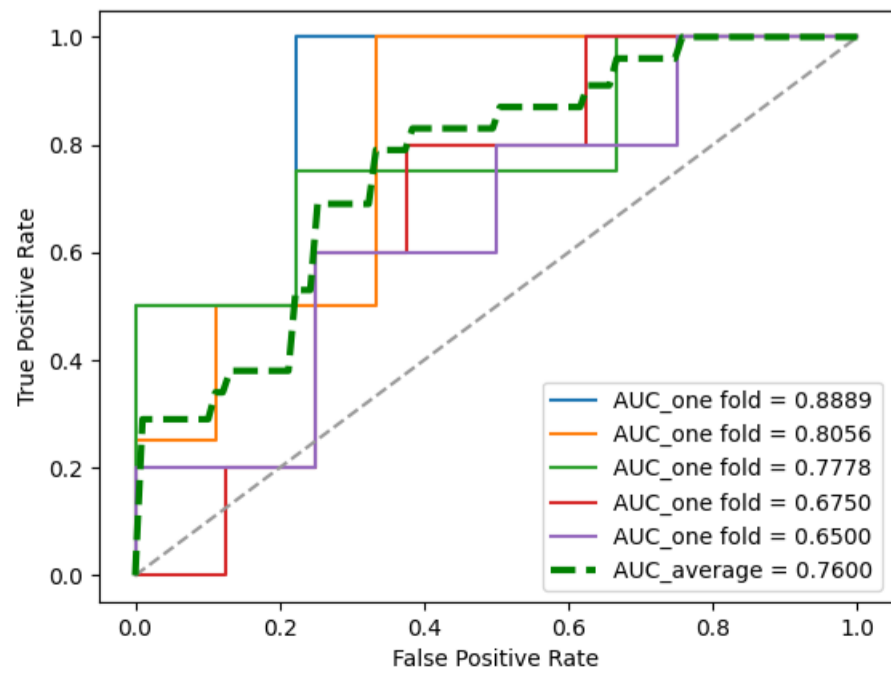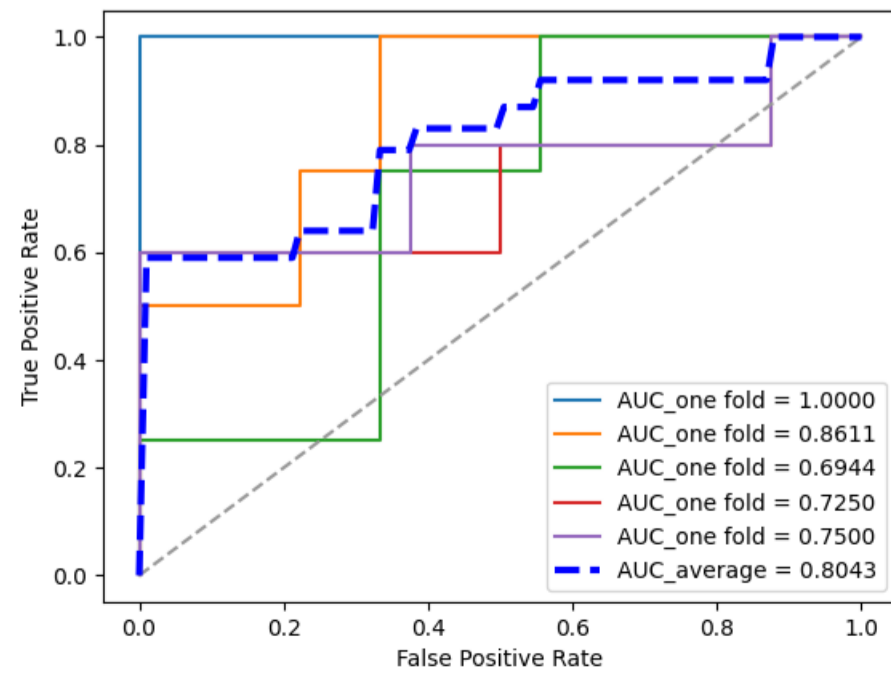

Supplement: Supplementary file 1 [file cancers-15-01784-s001.zip › Figure S8.pdf]

**a**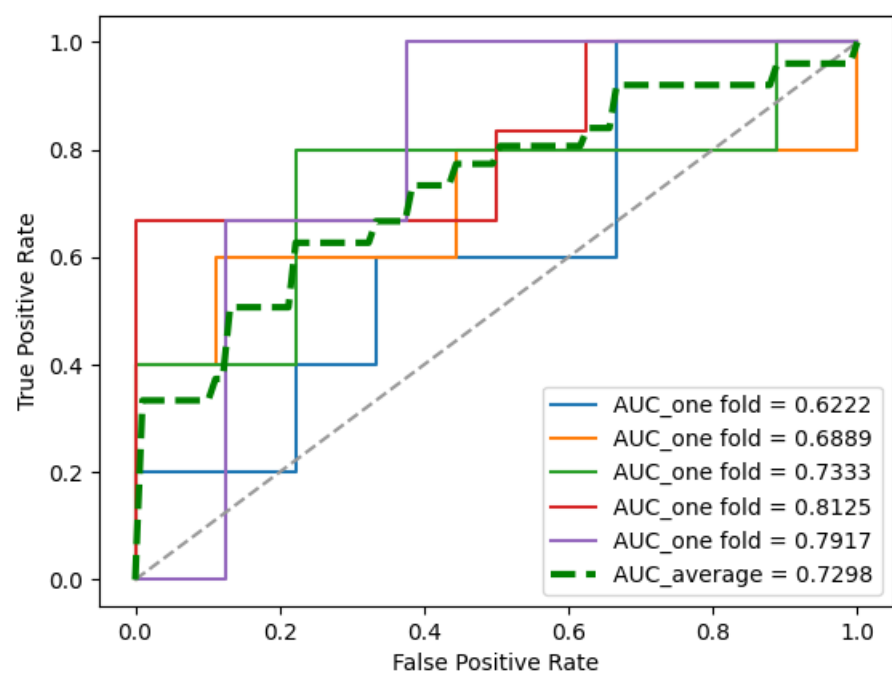**b**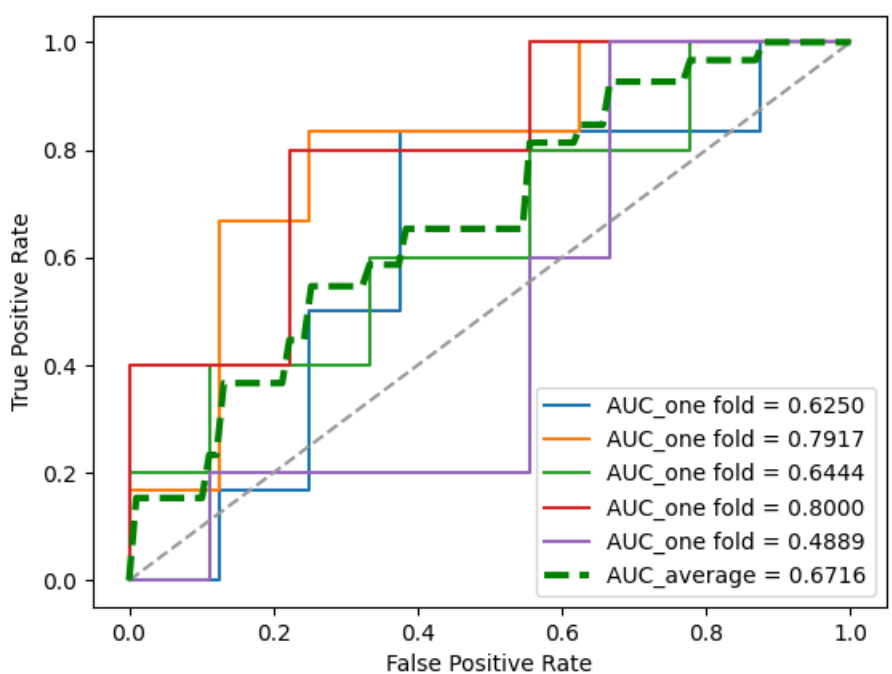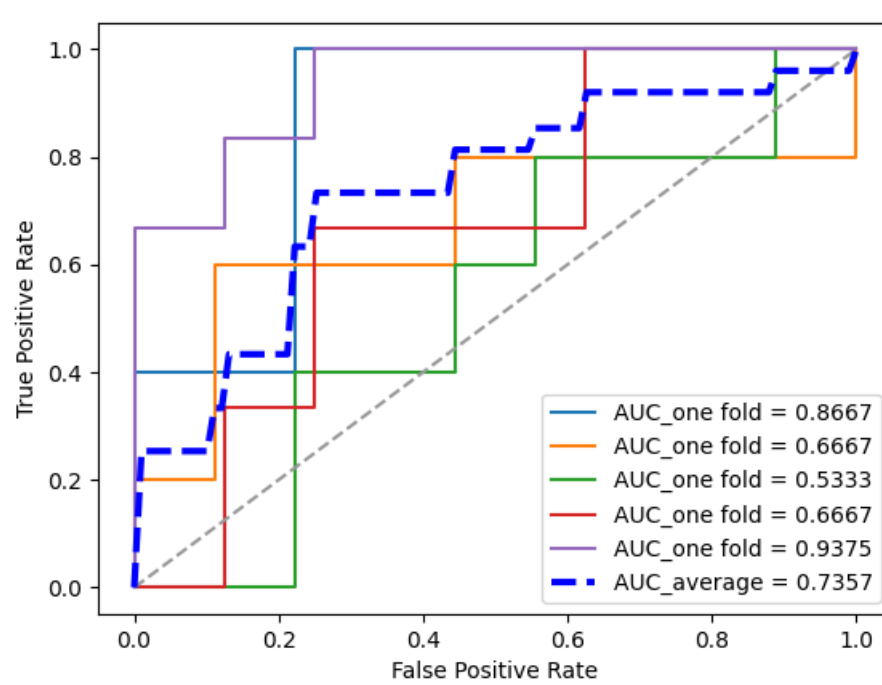**c**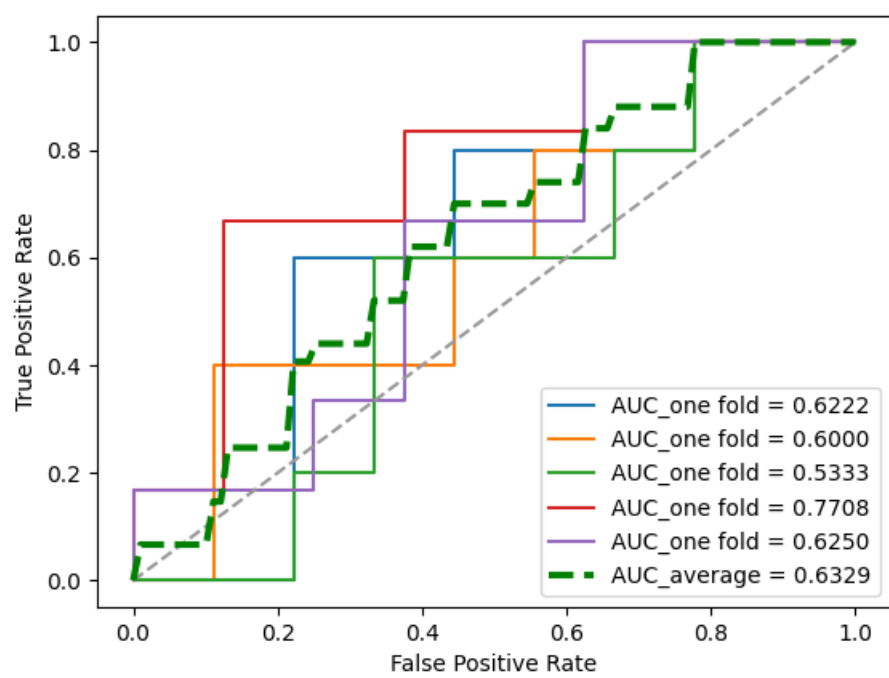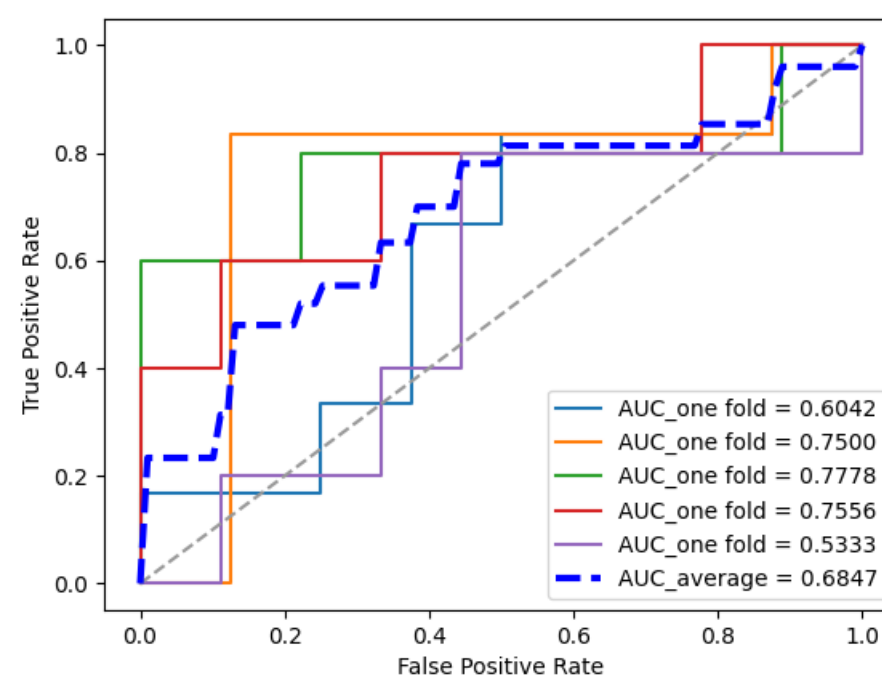**d**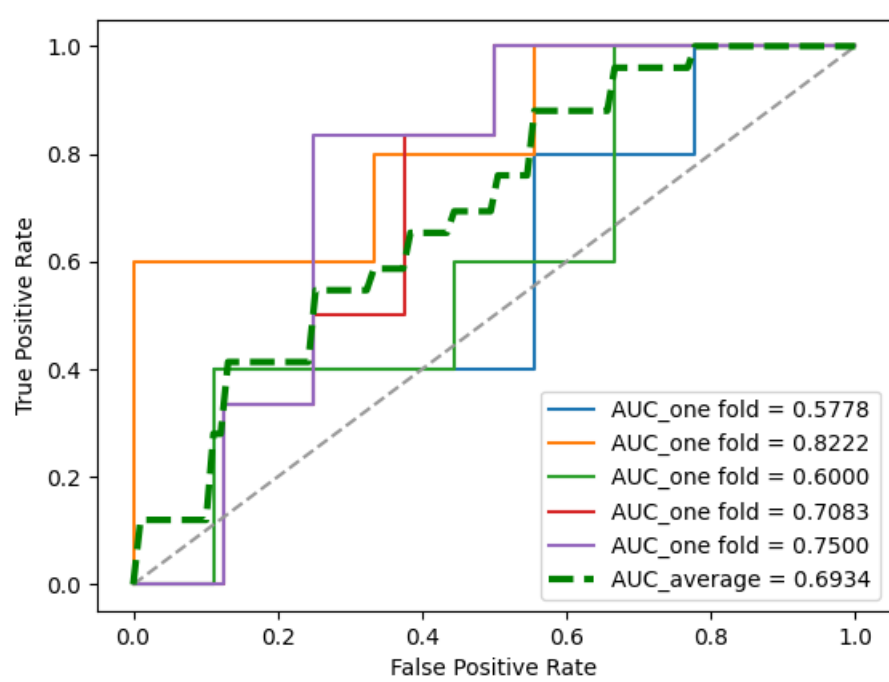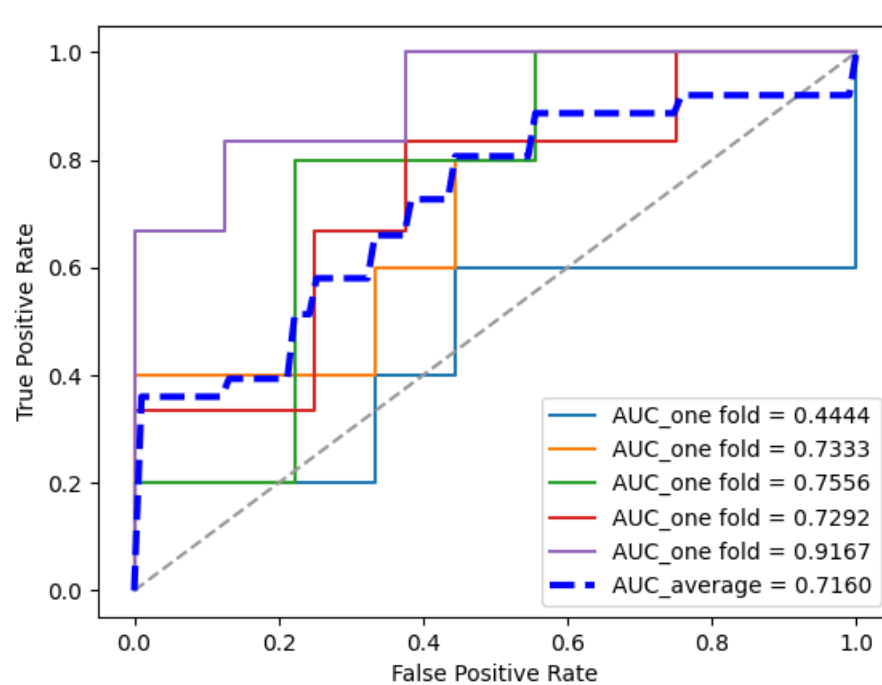

Supplement: Supplementary file 1 [file cancers-15-01784-s001.zip › Figure S9.pdf]
